# Genome-wide identification, evolutionary relationship and expression analysis of *AGO*, *DCL* and *RDR* family genes in tea

**DOI:** 10.1038/s41598-021-87991-5

**Published:** 2021-04-21

**Authors:** Debasish B. Krishnatreya, Pooja Moni Baruah, Bhaskar Dowarah, Soni Chowrasia, Tapan Kumar Mondal, Niraj Agarwala

**Affiliations:** 1 Department of Botany, Gauhati University, Jalukbari, Guwahati, Assam 781014 India; 2 ICAR-National Institute for Plant Biotechnology, IARI, LBS Building, Pusa, New Delhi, 110012 India

**Keywords:** Abiotic, Biotic, Drought, Computational biology and bioinformatics

## Abstract

Three gene families in plants viz. Argonaute (*AGOs*), Dicer-like (*DCLs*) and RNA dependent RNA polymerase (*RDRs*) constitute the core components of small RNA mediated gene silencing machinery. The present study endeavours to identify members of these gene families in tea and to investigate their expression patterns in different tissues and various stress regimes. Using genome-wide analysis, we have identified 18 *AGO*s, 5 *DCL*s and 9 *RDR*s in tea, and analyzed their phylogenetic relationship with orthologs of *Arabidopsis thaliana*. Gene expression analysis revealed constitutive expression of *CsAGO1* in all the studied tissues and stress conditions, whereas *CsAGO10c* showed most variable expression among all the genes. *CsAGO10c* gene was found to be upregulated in tissues undergoing high meristematic activity such as buds and roots, as well as in *Exobasidium vexans* infected samples. *CsRDR2* and two paralogs of *CsAGO4*, which are known to participate in biogenesis of hc-siRNAs, showed similarities in their expression levels in most of the tea plant tissues. This report provides first ever insight into the important gene families involved in biogenesis of small RNAs in tea. The comprehensive knowledge of these small RNA biogenesis purveyors can be utilized for tea crop improvement aimed at stress tolerance and quality enhancement.

## Introduction

Gene regulation in eukaryotes depends on post-transcriptional RNA interference mechanisms which is mediated by the action of the small RNAs (sRNAs). Gene silencing molecules like miRNAs and siRNAs are not only responsible for endogenous regulation of gene expression but are also involved in cross-kingdom mutualistic relations and interaction networks^1^. The use of RNAi technology by involving artificial miRNAs has also been an effective control measure against various biotic threats to plants^1,2^. Since RNA silencing mechanism is important for various regulatory aspects of plants, so a comprehensive understanding of the components of this machinery is needed. The RNA dependent RNA polymerases (RDRs) and Dicer-like proteins (DCLs) are directly involved in small RNA biogenesis, whereas Argonaute (AGO) constitutes a significant component of the RNA induced silencing complex (RISC)^3^. RDRs are responsible for the synthesis of dsRNAs using an RNA template, whereas DCLs are responsible for cleavage of the dsRNAs to form 21–24 nucleotide long functional small RNAs. These sRNAs, either miRNAs or any class of siRNAs, get incorporated into the RISC to drive the gene silencing machinery^4^. The sRNAs bind to specific AGO proteins and then guide the RISC to their corresponding target genes through complementary base pairing between target mRNA and the guide strand of the sRNA. This mode of gene regulation may be mediated by two approaches, viz. target mRNA cleavage or translational inhibition^5^.

The AGO proteins of plants and animals can be grouped into three types based on the nature of small RNAs with which they are associated. The first category of AGO proteins is known to interact predominantly with miRNAs and siRNAs, whereas the second category known as the PIWI proteins are exclusively found in animals which interact with PIWI-interacting RNAs (piRNAs). A third category of AGO proteins, which bind to secondary siRNAs, was reported in worms^6^. Several studies have suggested the presence of four typical domains in AGO proteins viz. N terminal domain (Argo-N), PAZ domain, MID domain and PIWI domain^7^. PAZ domain contains a nucleotide-binding pocket that anchors the two nucleotide 3′ overhangs of the small RNAs generated after RNase III-like activity of DCLs^8^. The PIWI domain exhibits extensive functional homology to RNase H and is known to impart ‘slicer’ activity of the AGO proteins^9^. The MID domain is known to bind the 5′ phosphates of small RNAs and anchors small RNAs onto the AGO proteins^10^. The Argo-N domain may facilitate the separation of the small RNA:target duplex after slicing by interrupting the duplex structure^11^. In addition to these domains, two linker domains viz. Argo-L1 and Argo-L2 may be present between the ArgoN-PAZ lobes and PAZ-Piwi lobes respectively. In plants, different species exhibit the presence of different numbers of *AGOs* in their genome. For instance, 10 *AGOs* have been reported in *Arabidopsis,* 13 in *Citrus,* maize and rice possess 17 and 19 *AGOs* respectively, whereas *Saccharum* has been reported to consist of 21 *AGO* genes in its genome^12–14^.

The DCLs are found to have six different conserved domains viz., DEAD-box helicase, Helicase C-terminal domain, a Dicer dimerization domain, PAZ, Ribonuclease-III and dsRNA binding motif. However, one or more domains mentioned above may be missing even in a functional DCL protein^15^. RDRs are represented by only one unique conserved domain in their sequence i.e., RNA-dependent RNA polymerase (RdRP)^14^.

Tea is popular as the most consumed non-alcoholic beverage all over the world as it provides numerous secondary metabolites that account for its rich taste and health benefits. Studies concerning miRNA-mediated regulation of gene expression in tea under various forms of biotic and abiotic stresses have been carried out^16–21^. The availability of annotated tea genomes has given a wider scope for understanding of genes associated with sRNA biogenesis and function. The tea genome size has been estimated to be about 2.94 Gb, assembled in 15 pseudo-chromosomes which anchor about 86.73% of the assembled sequences^22^. Such a considerable genome size corresponds to a large scale expansion of gene families. Identification of miRNAs and their putative target genes have well been facilitated by the availability of reference genome of tea for both the CSS (*C. sinensis* var. *sinensis*) and CSA (*C. sinensis* var. *assamica*) varieties. Differentially expressed miRNAs responsible for regulating the expression of genes related to biotic and abiotic stresses, accumulation of secondary metabolites and growth and development in tea, have also been reviewed recently^23,24^. Genome-wide analysis of the *AGO*, *DCL* and *RDR* gene families will decipher the diversity in these gene families and their function in this important commercial crop.

## Results

### Genome-wide identification and domain analysis of AGOs, DCLs and RDRs in *C. sinensis*

**Figure 1:**
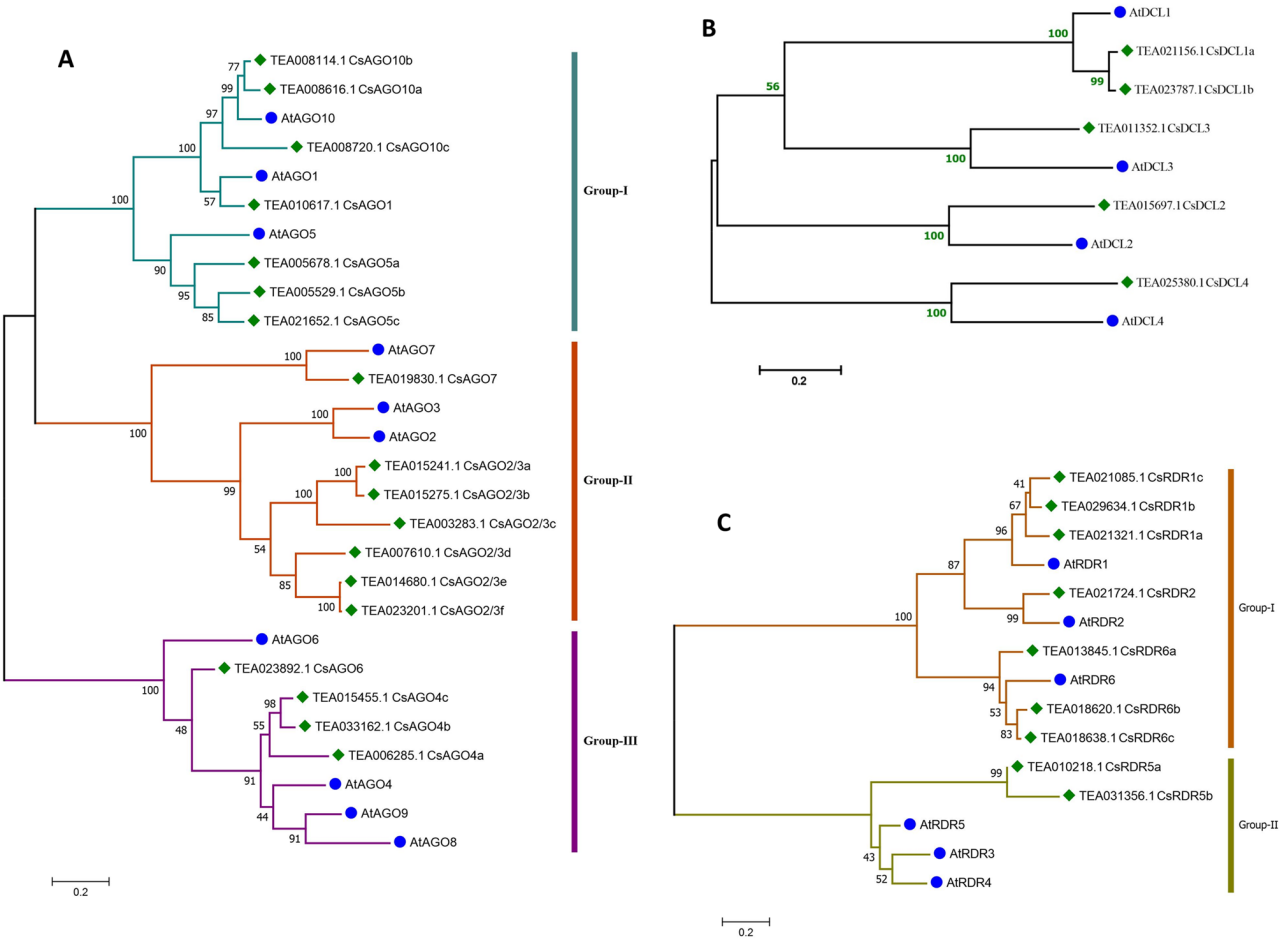
Phylogenetic trees showing relationships between (**A**) AGOs, (**B**) DCLs and (**C**) RDRs of *C. sinensis* and *A. thaliana.* The trees were constructed using the maximum likelihood method and a bootstrap replicate of 1000. The trees with the highest bootstrap support for each gene class have been shown here.

To perform genome-wide identification of the *AGO*, *DCL* and *RDR* gene families in *C. sinensis*, we obtained the Hidden Markov Model (HMM) profiles of the conserved domains and searched all the genes of *C. sinensis* present in Tea Plant Information Archive (TPIA) database for the presence of AGO, DCL and RDR specific conserved domains. Eighteen *CsAGOs* were identified after analysing against the pfam database for presence of the following AGO specific domains—Argo-N: N-terminal domain of AGO proteins; PAZ: a domain that anchors the 3′ end of the bound small RNA and Piwi_Ago-like: PIWI domain present in the C-terminal region. Similarly, five *DCLs* and twelve *RDRs* were identified in *C. sinensis* genome using HMM profiles of gene specific conserved domains viz., RNaseIII, PAZ and dsRNA binding motif (for DCLs) and RdRP (for RDRs) followed by analysis against the pfam database. Two genes (accession numbers TEA000774.1 and TEA010224.1) with RdRP domains were further discarded as their lengths were small i.e., 38 and 73 amino acids, respectively for considering them as functional and without any close phylogenetic relationship with other identified *CsRDRs*. Further, TEA007002.1 was also discarded due to anomalies in lengths of its genomic and coding sequences. Thus after assessing of structural integrity of the conserved domains, 18 *CsAGOs*, 5 *CsDCLs* and 9 *CsRDRs* have been identified in the tea genome. The identified genes were named according to the phylogenetic relationships exhibited by their corresponding protein sequences with AGOs, RDRs and DCLs of *A. thaliana* obtained from TAIR (Table 1 and Fig. 1). The multiple sequence alignment showed high sequence similarity between the protein sequences, particularly in conserved functional domain regions (Supplementary Figure S1a–c).

**Table 1:** Properties of identified *CsAGO*, *CsDCL* and *CsRDR* genes.

Sl. no.	Assigned ID	Accession	Location	Start (5′)	Stop (3′)	Strand	Transcript length	Protein length
1	*CsAGO1*	TEA010617	Scaffold2091	586,414	597,932	+	3603	1200
2	*CsAGO2/3a*	TEA015241	Scaffold409	653,585	647,587	−	3168	1055
3	*CsAGO2/3b*	TEA015275	Scaffold409	542,266	536,576	−	3219	1072
4	*CsAGO2/3c*	TEA003283	Scaffold622	1,919,612	1,908,711	−	2301	766
5	*CsAGO2/3d*	TEA007610	Scaffold1775	1,333,085	1,328,089	−	2751	916
6	*CsAGO2/3e*	TEA014680	Scaffold2339	505,271	495,573	−	3405	1134
7	*CsAGO2/3f*	TEA023201	Scaffold5319	119,575	104,830	−	3183	1060
8	*CsAGO4a*	TEA006285	Scaffold1609	1,048,204	1,065,155	+	2871	956
9	*CsAGO4b*	TEA033162	Scaffold371	591,571	577,022	−	2838	945
10	*CsAGO4c*	TEA015455	Scaffold17947	50,775	97,852	+	4386	1461
11	*CsAGO5a*	TEA005678	Scaffold3329	2,163,110	2,154,877	−	3372	1123
12	*CsAGO5b*	TEA005529	Scaffold2891	638,503	630,782	−	2919	972
13	*CsAGO5c*	TEA021652	Scaffold3619	1,616,391	1,626,257	+	3060	1019
14	*CsAGO6*	TEA023892	Scaffold366	435,162	444,062	+	2706	901
15	*CsAGO7*	TEA019830	Scaffold660	687,517	683,178	−	3084	1027
16	*CsAGO10a*	TEA008616	Scaffold347	412,492	400,307	−	2994	997
17	*CsAGO10b*	TEA008114	Scaffold736	2,967,158	2,956,963	−	2997	998
18	*CsAGO10c*	TEA008720	Scaffold2968	415,128	391,947	−	2802	933
19	*CsDCL1a*	TEA021156	Scaffold2220	297,568	282,536	−	4491	1496
20	*CsDCL1b*	TEA023787	Scaffold6409	125,218	129,705	+	2652	883
21	*CsDCL2*	TEA015697	Scaffold3698	1,259,866	1,288,358	+	4104	1367
22	*CsDCL3*	TEA011352	Scaffold4138	1,757,571	1,714,236	−	5202	1733
23	*CsDCL4*	TEA025380	Scaffold423	1,226,694	1,153,631	−	4680	1559
24	*CsRDR1a*	TEA021321	Scaffold1968	1,006,049	991,064	−	3459	1152
25	*CsRDR1b*	TEA029634	Scaffold872	905,729	927,644	+	3609	1202
26	*CsRDR1c*	TEA021085	Scaffold2268	122,465	84,100	−	3492	1163
27	*CsRDR2*	TEA021724	Scaffold1551	145,105	159,756	+	3450	1149
28	*CsRDR5a*	TEA010218	Scaffold4444	610,116	625,490	+	2481	826
29	*CsRDR5b*	TEA031356	Scaffold1203	903,839	921,291	+	1029	342
30	*CsRDR6a*	TEA013845	Scaffold2753	994,451	989,757	−	1977	658
31	*CsRDR6b*	TEA018620	Scaffold3982	713,824	732,349	+	3921	1306
32	*CsRDR6c*	TEA018638	Scaffold3982	773,264	779,257	+	3642	1213

### Phylogenetic classification of identified genes

To define the homology between the identified protein sequences, phylogenetic trees of the 18 CsAGOs, 5 CsDCLs and 9 CsRDRs were constructed along with designated AtAGOs, AtDCLs and AtRDRs found in TAIR using maximum likelihood (ML) approach. The resulting trees produced well-resolved phylogeny with high bootstrap support. It was evident that *C. sinensis* AGO family proteins can be classified into three major clusters, with Group-I and II comprising of seven CsAGOs each, whereas group- III comprised of four members (Fig. 1A). The best ML scoring rooted tree indicates that Group-III AGOs probably emerged earliest during evolution compared to Group-I and II. Maximum-likelihood based phylogenetic tree constructed for CsRDRs and the six designated AtRDRs showed the presence of two major clusters with Group-I represented by seven members of CsRDR family whereas only two CsRDR proteins present in Group-II (Fig. 1B). However, no such substantive groups or clusters were seen in the topological pattern of DCLs, which indicated a more or less parallel evolutionary trend for the *DCL* genes (Fig. 1C).

### Evolutionary relationship between *C. sinensis* and other plant AGOs, DCLs and RDRs

To determine the evolutionary relationship between tea and other plants in terms of proteins involved in small RNA machinery, we comprehensively analysed the phylogeny between single orthologs of CsAGOs, CsDCLs and CsRDRs found in representative species of all plant lineages. For this objective, 61 orthologous protein sequences of CsAGO1 and CsDCL1a, and 58 orthologous sequences of CsRDR1a were identified from different species belonging to algae, bryophytes, lycophytes, monocots and dicots. These protein sequences harboured characteristic domains and motifs of AGO, RDR and DCL proteins (Supplementary Table S1a–c). All the listed plant species in Phytozome-12 were selected and NJ trees were constructed using the orthologous protein sequences with 5000 bootstrap replicates.

The resulting NJ tree obtained for AGO contained five major clusters with significant bootstrap values. The topology of the tree depicts lower plants as the ancestors of the *AGO* gene family as they have settled in the basal group-I. AGO protein of *C. sinensis* finds its place in group-V alongside majority of the eudicots including *Arabidopsis* (Supplementary Figure S2a). However, topology of the tree constructed for RDR proteins did not follow typical evolutionary pattern with the basal group comprising a mix of algae, bryophyte and eudicots (Supplementary Figure S2b). The presence of only one representative conserved domain across the complete peptide sequence of RDRs of all the plant lineages may be responsible for retrieving such a tree with low bootstrap support and undefined distribution of plant species across the clusters. Orthologs of DCL proteins in the considered plant species mostly exhibited parallel evolution and no well-defined clusters or groups can be identified based on chronology of plant kingdom evolution (Supplementary Figure S2c). This pattern is also similar to the evolutionary pattern of paralogous DCLs of tea, as described in the previous section.

### The *CsAGO*, *CsDCL* and *CsRDR* gene families

In this study, eighteen *AGO* members with all the characteristic domains were found in the tea tree genome. The nucleotide length of these genes varied between 2301 bp (*CsAGO2/3c*) to 4386 bp (*CsAGO4c*), while their encoded protein lengths ranged between 766 (*CsAGO2/3c*) to 1461 (*CsAGO4c*) amino acid residues. On an average, *CsDCL* genes exhibited longer nucleotide lengths which ranged from 2652 bases in *CsDCL1b* to 5202 bases in *CsDCL3*. The nucleotide lengths of *CsRDRs* ranged from 1029 bp (*CsRDR5b*) to 3921 bp (*CsRDR6b*) with their corresponding peptide lengths ranging from 342 to1306 amino acid residues respectively. The *AGO* genes were mostly oriented on reverse strands with only 5 genes being positioned on the forward strand. Similarly two *DCLs* were located on the forward strand and 3 *DCLs* on the reverse strand. However, *RDR* genes were mostly were located on the forward strand with only three genes oriented on the reverse strand (Table 1).

The ProtParam tool analysis showed significant differences in molecular weights of AGO (ranging from 85.93 to 161.84 kDa), DCL (98.7 to 194.14 kDa) and RDR (38.8 to 148.03 kDa) proteins of *C. sinensis*. Most of the CsAGOs have a relatively high isoelectric point (pI) (theoretical pI > 9) except CsAGO4a, b and c. However pI values were comparatively lower in CsDCLs and CsRDRs with most of them exhibiting a theoretical pI value of less than 8. All the identified proteins have negative GRAVY (Grand Average of Hydropathicity) values which implies that genes of all the three families are non-polar or hydrophilic in nature. Comparatively, CsAGOs are typically more hydrophilic than the members of CsDCL and CsRDR families. Out of all the 32 enlisted proteins, only seven of them viz. CsAGO2/3d, CsDCL1a, CsDCL1b, CsRDR5a, CsRDR6a, CsRDR6b and CsRDR6c have an Instability index (II) value less than 40 and hence can be considered as stable proteins. All CsDCLs, four CsRDRs and only one CsAGO have more number of negatively charged residues (Asp + Glu) as compared to positively charged residues (Arg + Lys) (Table 2).

**Table 2:** Physico-chemical properties of AGO, DCL and RDR proteins of *C. sinensis.*

Proteins	Accession	Mol. wt. (kDa)	pI	(Asp + Glu)	(Arg + Lys)	Total atoms	II	Aliphatic index	GRAVY
CsAGO1	TEA010617	132.077	9.39	110	144	18,503	51.63	77.02	− 0.420
CsAGO2/3a	TEA015241	116.347	9.28	106	136	16,285	42.51	74.53	− 0.459
CsAGO2/3b	TEA015275	117.989	9.31	108	139	16,498	40.27	73.90	− 0.473
CsAGO2/3c	TEA003283	85.930	9.62	78	112	12,090	45.08	77.58	− 0.616
CsAGO2/3d	TEA007610	102.060	9.25	96	122	14,350	39.24	82.30	− 0.341
CsAGO2/3e	TEA014680	128.096	9.08	123	149	18,033	44.60	83.82	− 0.357
CsAGO2/3f	TEA023201	119.975	9.20	112	140	16,868	43.81	79.84	− 0.458
CsAGO4a	TEA006285	106.596	8.86	101	116	14,976	45.69	81.43	− 0.321
CsAGO4b	TEA033162	105.464	8.72	103	113	14,860	41.39	84.63	− 0.300
CsAGO4c	TEA015455	161.840	6.17	179	164	22,703	46.11	87.45	− 0.245
CsAGO5a	TEA005678	126.351	9.44	116	156	17,797	43.40	81.32	− 0.443
CsAGO5b	TEA005529	108.701	9.45	95	130	15,311	45.44	80.90	− 0.368
CsAGO5c	TEA021652	114.239	9.58	106	145	16,048	53.75	76.04	− 0.560
CsAGO6	TEA023892	100.718	9.44	86	117	14,236	46.52	85.13	− 0.331
CsAGO7	TEA019830	116.723	9.24	100	128	16,424	53.88	82.76	− 0.435
CsAGO10a	TEA008616	112.132	9.27	101	134	15,758	44.30	79.69	− 0.455
CsAGO10b	TEA008114	112.097	9.31	101	135	15,766	43.33	79.90	− 0.455
CsAGO10c	TEA008720	105.275	9.07	94	116	14,816	43.98	84.95	− 0.373
CsDCL1a	TEA021156	166.298	5.83	199	172	23,364	39.34	88.95	− 0.261
CsDCL1b	TEA023787	98.697	5.65	117	99	13,868	38.41	88.78	− 0.236
CsDCL2	TEA015697	154.501	6.30	157	143	21,736	44.52	94.44	− 0.129
CsDCL3	TEA011352	194.137	7.28	205	204	27,337	44.59	91.96	− 0.234
CsDCL4	TEA025380	176.031	6.15	195	173	24,662	42.60	86.45	− 0.221
CsRDR1a	TEA021321	131.820	5.56	158	133	18,458	45.01	84.66	− 0.244
CsRDR1b	TEA029634	137.362	7.71	149	151	19,243	41.42	82.79	− 0.292
CsRDR1c	TEA021085	113.126	8.32	148	155	18,696	41.96	84.49	− 0.298
CsRDR2	TEA021724	129.666	6.98	136	134	18,198	41.78	86.27	− 0.230
CsRDR5a	TEA010218	94.037	6.17	108	100	13,173	36.80	84.13	− 0.327
CsRDR5b	TEA031356	38.794	7.61	41	42	5462	46.40	90.06	− 0.143
CsRDR6a	TEA013845	74.158	6.13	80	74	10,363	31.84	81.81	− 0.309
CsRDR6b	TEA018620	148.025	7.29	167	167	20,752	36.13	82.47	− 0.337
CsRDR6c	TEA018638	138.266	8.33	157	165	19,388	35.16	80.59	− 0.377

### Structure of genes and conserved motifs in their encoded proteins

**Figure 2:**
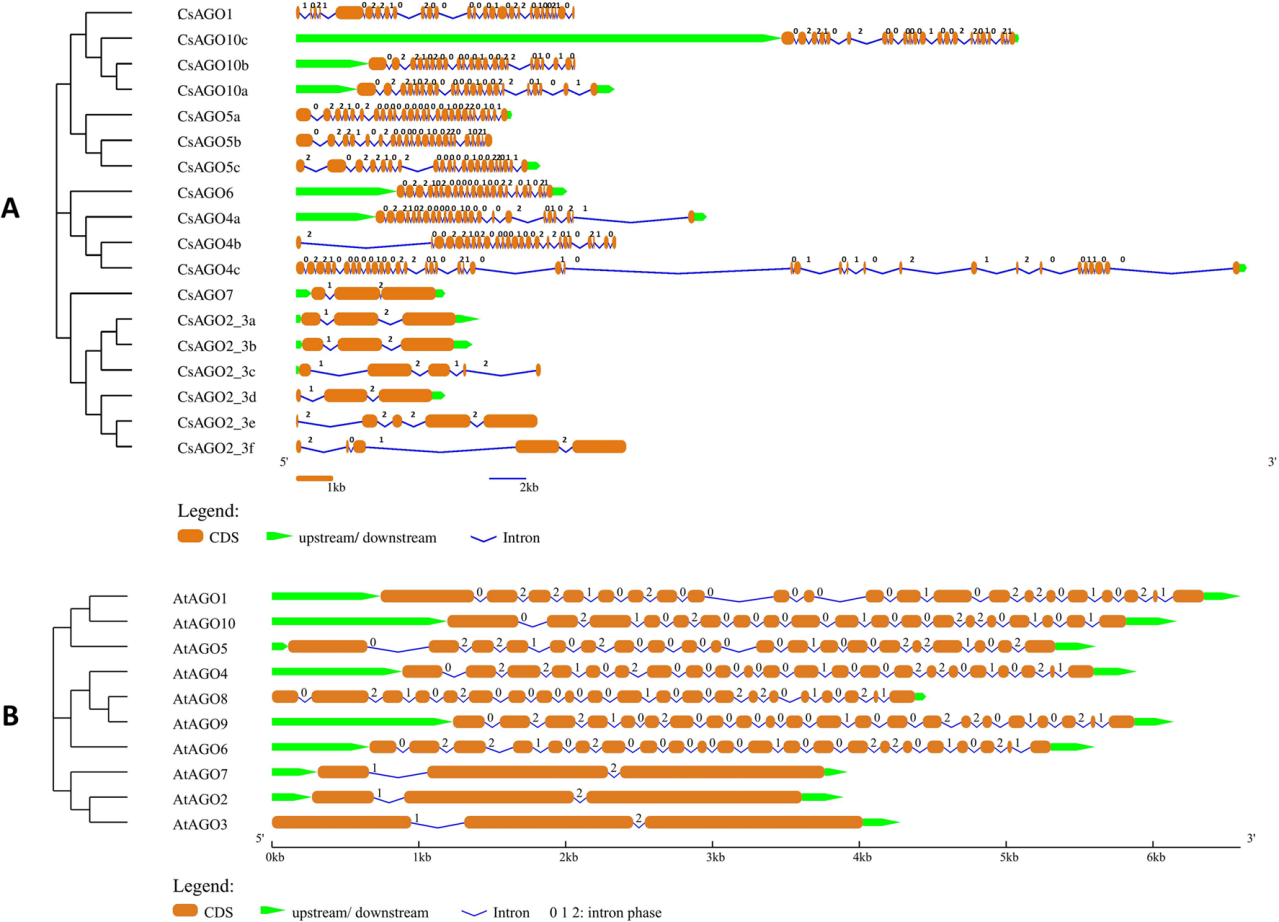
Gene structures showing the organization of exons and introns, and associated intron phases [0, 1 and 2] of (**A**) *CsAGO* and (**B**) *AtAGO* genes. The NJ phylogenetic tree of CDS is shown on the left side of the figure.

**Figure 3:**
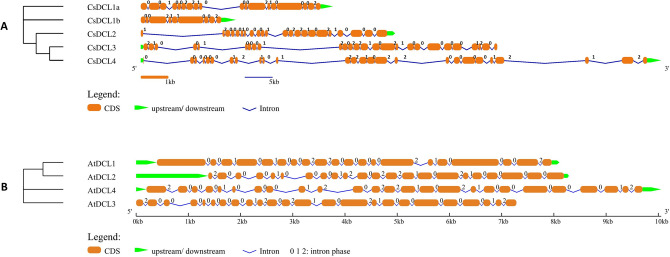
Gene structures showing the organization of exons and introns, and associated intron phases [0, 1 and 2] of (**A**) *CsDCL* and (**B**) *AtDCL* genes. The NJ phylogenetic tree of CDS is shown on the left side of the figure.

**Figure 4:**
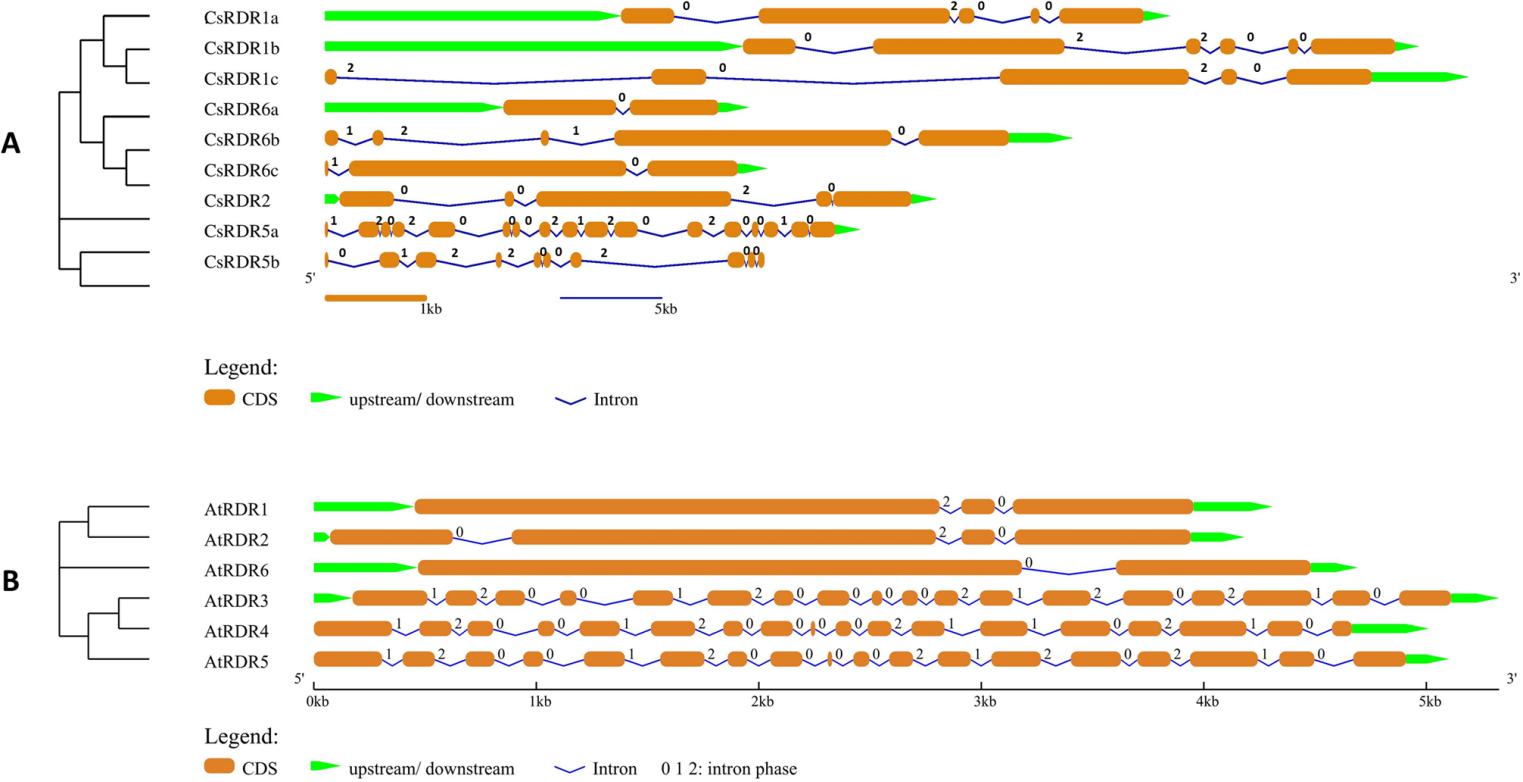
Gene structures showing the organization of exons and introns, and associated intron phases [0, 1 and 2] of (**A**) *CsRDR* and (**B**) *AtRDR* genes. The NJ phylogenetic tree of CDS is shown on the left side of the figure.

The exon–intron organisation of the genes is portrayed to elucidate the structural diversity of the *CsAGO*, *CsDCL* and *CsRDR* family genes. The number of exons varied significantly among the *CsAGO* genes, with *CsAGO4c* comprising 37 exons whereas *CsAGO7* and all paralogs of *CsAGO2/3* comprising 3–5 exons only. The length of introns also varies among different *CsAGO*s. Most of the introns in *CsAGO7* and paralogs of *CsAGO2/3* are in intron phase-2 (i.e., disrupting a codon between its second and third bases), whereas in rest of the *CsAGO* genes most introns are in phase-0 (i.e., present between two separate triplet codons). Most of the genes comprised more than one type of introns, except for *CsAGO2/3e* which consisted of four phase-2 introns (Fig. 2A). Significant differences in terms of loss/gain of exons and their arrangements was observed among genes belonging to different phylogenetic sub-trees, which may further add an element of diversity in structure and functions of *CsAGOs*. Besides, *CsAGOs* comprise of more phase-2 introns than phase-0 introns, unlike their *A. thaliana* homologs, where phase-0 introns outnumbered phase-2 introns (Fig. 2B). Moreover, *AtAGOs* exhibited a similar pattern in distribution of exons in their structures according to the clusters formed by *AtAGOs* and *CsAGOs* in the phylogenetic tree. Distribution of exons among the members of *CsDCL* genes showed that exon numbers varied from 26 in *CsDCL3* and *CsDCL4* to 9 in *CsDCL1b* (Fig. 3A). The *CsRDR* genes consisted of very lesser number of exons ranging from 2 to 6, except for *CsRDR5a* and *CsRDR5b,* consisting of 17 and 10 exons respectively (Fig. 4A). The introns mainly belonged to phase-0 type among the *CsDCL* and *CsRDR* genes, similar to the distribution patterns of introns among their *A. thaliana* counterparts (Figs. 3B and 4B). Several differences in terms of loss or gain of introns, intron phases and their shuffling were observed among the genes thus adding structural and functional diversity to the members of the three gene families.

**Figure 5:**
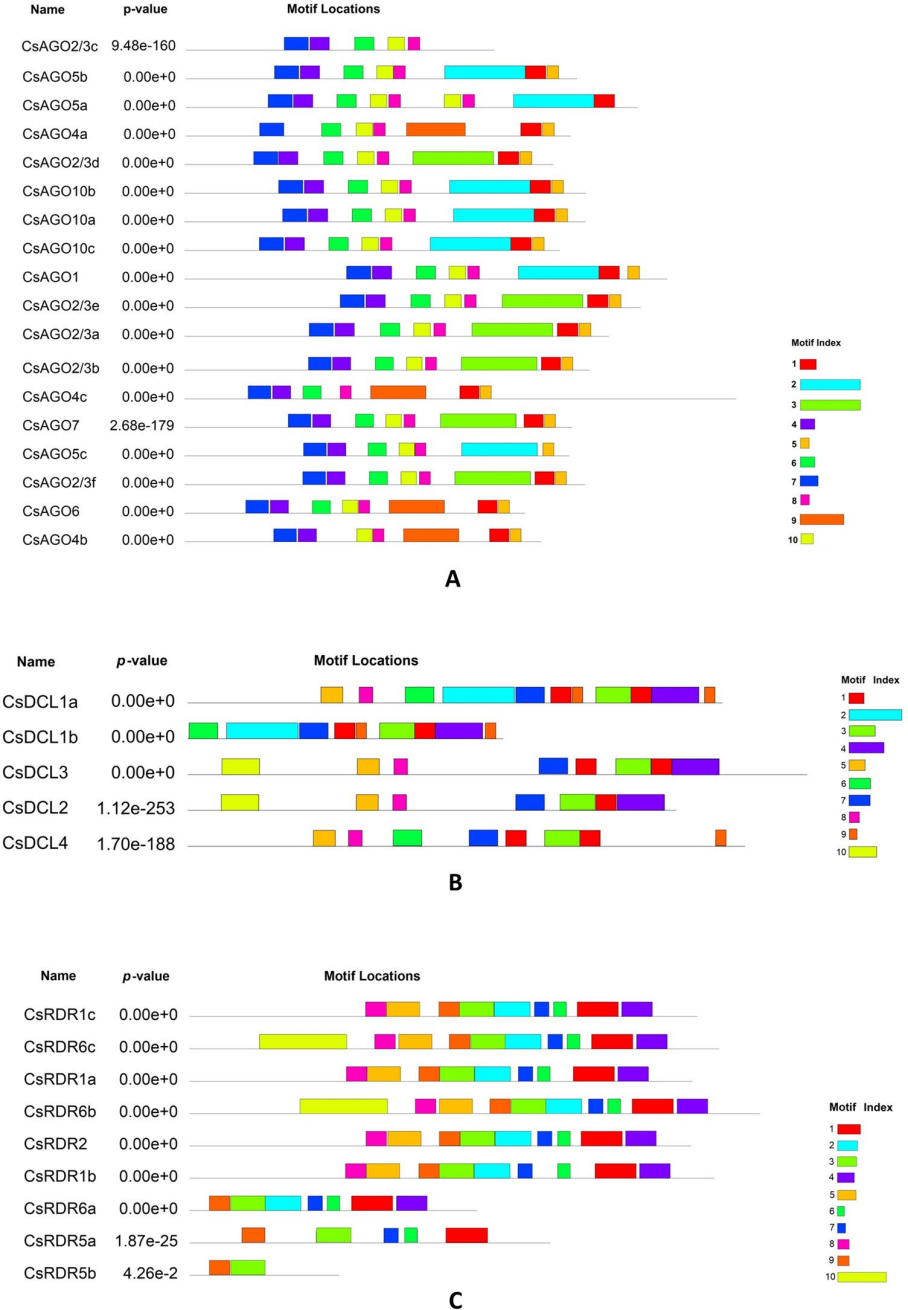
Distribution of conserved motifs identified in proteins encoded by (**A**) *CsAGOs*, (**B**) *CsDCLs* and (**C**) *CsRDRs*. The motif index represents the corresponding motif number depicted in Supplementary Figure S3 and Supplementary Table S2 for motif annotation.

The conserved motifs of the AGO, DCL and RDR proteins were detected using the online MEME server (Multiple Expectation Maximization for Motif Elicitation). For CsAGOs, eight motifs out of at least ten were part of known domains according to Pfam codes (Fig. 5A and Supplementary Table S2a). Motifs 1, 2, 3 and 9 are associated with Piwi domain, whereas motifs 4, 6, 7 and 10 represent Argo-L1, PAZ, Argo-N and Argo-L2 respectively. The functions or secondary associations of motifs 5 and 8 are still unknown. Conserved motif analysis of CsDCL proteins resulted in recognition of five motifs mapped to known domains. According to pfam annotation, motifs 2 and 7 represent parts of PAZ domain whereas motifs 1, 5 and 10 represent RNaseIII, Helicase C-terminal and DEAD box domains respectively (Fig. 5B and Supplementary Table S2b). Since RdRP is the only conserved domain present in the plant RDRs, most of the motifs identified in CsRDRs are parts of the RdRP domain, with motifs 4 and 10 not having any defined annotations in pfam (Fig. 5C and Supplementary Table S2c). The logos of the corresponding motifs have been presented in Supplementary Figure S3.

### Potential miRNA target sites in the identified gene transcripts

miRNAs regulate key biological processes such as growth, signal transduction, response to stress etc. AGO, DCL and RDR proteins are themselves involved in miRNA biogenesis and thus identification of miRNA target sites in the transcripts of these gene families may help to elucidate any potential self-regulatory or feedback mechanisms in plant miRNA biogenesis. Target analysis using the set of all plant miRNAs deposited in miRBase was carried out with expect value (e-value) threshold of 2.0, which revealed three potential miRNA target sites in *CsAGO2/3a*, two such sites in *CsAGO2/3c* and one target site each in *CsAGO4a, CsAGO5c, CsAGO10b, CsRDR1c, CsRDR6b* and *CsRDR6c*. No putative target sites within the e-value cut-off of 2.0 could be detected in *CsDCL* genes. The identified miRNAs are located on the 3′ strand of the stem-loop hairpin precursors. The UPE (Unpaired energy) value varied from 8.597 (ath-miR5658) to 27.278 (bdi-miR169c-3p) (Supplementary Table S3). The UPE represents the relative energy required to open the miRNA secondary structure around its target mRNA and thus a lower value corresponds with a better chance of contact between miRNA and target mRNA.

### *Cis*-acting regulatory elements

**Figure 6:**
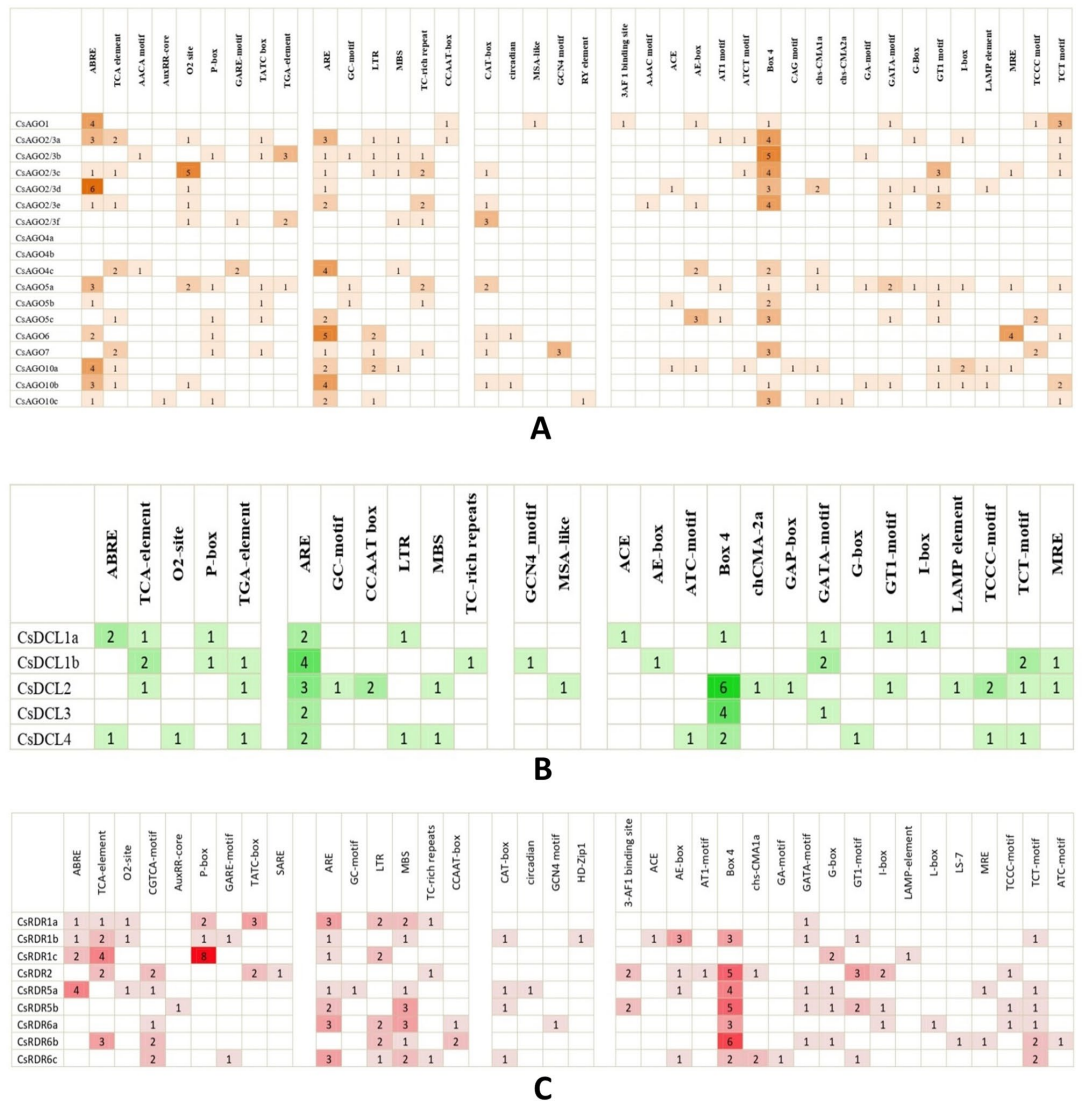
Number of each cis-acting element in the promoter region (2 kb upstream of translation start site) of respective genes belonging to (**A**) *CsAGO*, (**B**) *CsDCL* and (**C**) *CsRDR*. The elements have been separated into four distinct groups (by using a blank column between two groups) based on their functional properties (categories from left to right—hormone responsive; stress and defence response; plant growth and development; light responsive).

Various *cis*-acting regulatory elements were found in the promoter regions (2 kb upstream of translation start site) of the identified genes. Primarily TATA box, which is one of the major regulatory components and is present around 30 bases before the translation start site, has been detected in the upstream sequences of most of the genes. Common *cis*-acting enhancers and regulatory elements viz., CAAT box and A-box are also present in promoter regions of a number of genes. Other *cis*-acting elements detected in *CsAGOs*, *CsDCLs* and *CsRDRs* can be classified into four groups based on their functional properties viz. hormone responsive elements, stress and defence response, plant growth and development and light-responsive elements. The number of these elements detected in promoter regions of each gene has been shown in Fig. 6.

### dn/ds values of orthologs of *AGOs*, *DCLs* and *RDRs* in *C. sinensis* and *A. thaliana*

The dn/ds values were calculated for the orthologous genes pairs of *CsAGO*, *CsDCL* and *CsRDR* with those of *A. thaliana* (Table 3). The dn/ds values were found to be less than 1 for all the entries implying that purifying or stabililizing selection has been the major evolutionary mechanism in these genes^25^.

**Table 3:** Synonymous and non-synonymous substitution rates of orthologous gene pairs.

Gene name	*A. thaliana* gene-ID	S-sites	N-sites	ds	dn	dn/ds	Divergence time (MYA)
*CsAGO1*	AT1G48410.1	730	2411	2.3054	0.1424	0.062	177.338
*CsAGO2/3a*	AT1G31280.1	693.7	2270.3	3.016	0.4384	0.145	232.000
*CsAGO2/3b*	AT1G31280.1	685	2288	2.9057	0.4463	0.154	223.515
*CsAGO2/3c*	AT1G31280.1	500.5	1722.5	2.4394	0.5372	0.220	187.646
*CsAGO2/3d*	AT1G31280.1	648.4	2054.6	2.3885	0.3841	0.161	183.731
*CsAGO2/3e*	AT1G31280.1	687.3	2294.7	2.2713	0.4584	0.202	174.715
*CsAGO2/3f*	AT1G31280.1	688	2249	2.0033	0.445	0.222	154.100
*CsAGO4a*	AT2G27040.1	627.6	2036.4	3.2404	0.2366	0.073	249.262
*CsAGO4b*	AT2G27040.1	595.4	2038.6	3.0558	0.1731	0.057	235.062
*CsAGO4c*	AT2G27040.1	585.5	1937.5	2.9976	0.1655	0.055	230.585
*CsAGO5a*	AT2G27880.1	620.4	2109.6	3.9804	0.2849	0.072	306.185
*CsAGO5b*	AT2G27880.1	670.9	2188.1	3.8707	0.3082	0.080	297.746
*CsAGO5c*	AT2G27880.1	634	2114	3.3537	0.3018	0.090	257.977
*CsAGO6*	AT2G32940.1	627.9	1982.1	1.9205	0.2331	0.121	147.731
*CsAGO7*	AT1G69440.1	689.4	2211.6	2.4501	0.2258	0.092	188.469
*CsAGO10a*	AT5G43810.1	692.2	2229.8	3.1338	0.114	0.036	241.062
*CsAGO10b*	AT5G43810.1	671.3	2250.7	3.4197	0.0963	0.028	263.054
*CsAGO10c*	AT5G43810.1	654.4	2129.6	3.261	0.1854	0.057	250.846
*CsDCL1a*	AT1G01040.1	1081	3326	2.356	0.141	0.060	181.231
*CsDCL1b*	AT1G01040.1	656.7	1965.3	2.1638	0.1073	0.050	166.446
*CsDCL2*	AT3G03300.1	948.1	3008.9	2.1394	0.3262	0.152	164.569
*CsDCL3*	AT3G43920.1	1107.2	3542.8	2.4104	0.4073	0.169	185.415
*CsDCL4*	AT5G20320.1	1002	3279	2.1895	0.4017	0.183	168.423
*CsRDR1a*	AT1G14790.1	736.8	2557.2	4.011	0.2683	0.067	308.538
*CsRDR1b*	AT1G14790.1	737.1	2547.9	8.4387	0.2341	0.028	649.131
*CsRDR1c*	AT1G14790.1	735.8	2558.2	3.4007	0.2312	0.068	261.592
*CsRDR2*	AT4G11130.1	819.4	2525.6	1.8328	0.2862	0.156	140.985
*CsRDR5a*	AT2G19930.1	479.1	1623.9	3.5722	0.3541	0.099	274.785
*CsRDR5b*	AT2G19930.1	224.9	786.1	14.5924	0.6591	0.045	1122.492
*CsRDR6a*	AT3G49500.1	496.2	1477.8	2.7386	0.1925	0.070	210.662
*CsRDR6b*	AT3G49500.1	842.3	2739.7	2.5387	0.2272	0.089	195.285
*CsRDR6c*	AT3G49500.1	847.8	2734.2	2.5503	0.2152	0.084	196.177

### Chromosomal location of *CsAGO*, *CsDCL* and *CsRDR* genes

**Figure 7:**
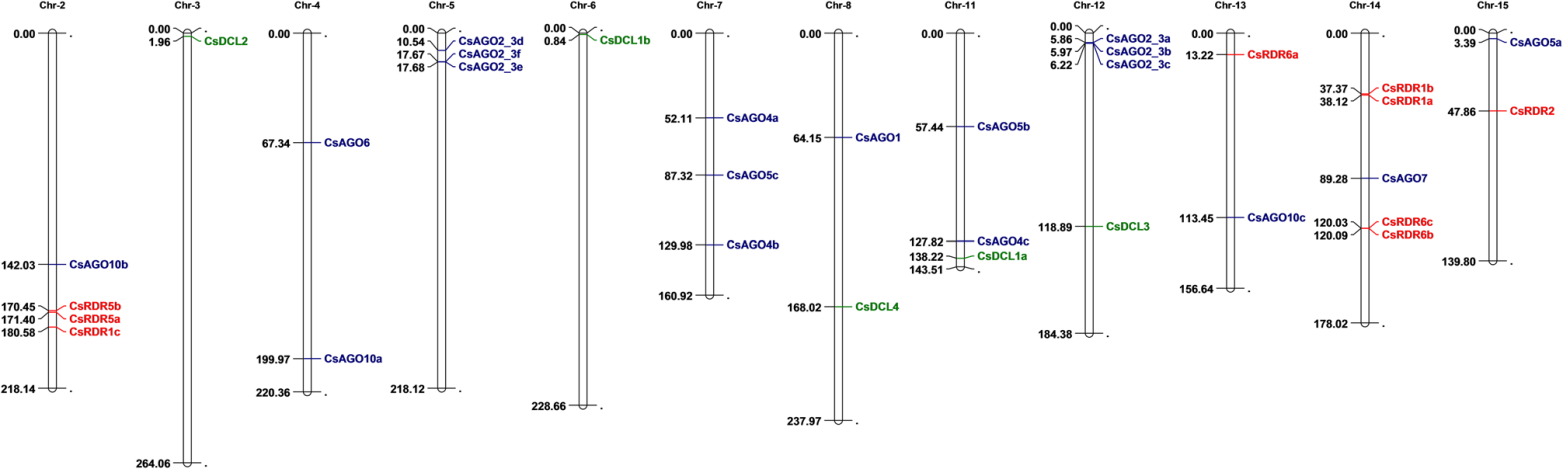
The distribution of *AGO, DCL* and *RDR* genes on pseudo-chromosomes of *C. sinensis*. Chromosome numbers have been indicated on the top of each chromosome. The position of each gene on the respective chromosome has been depicted in terms of kilobase-pairs by numbers beside each gene.

The tea genome has been recently assembled into 15 pseudo-chromosomes. Information regarding physical location of each of the gene was obtained by a blast search using sequences of the genes and the pseudo-chromosomes. The 32 genes under consideration were found to be located in 12 pseudo-chromosomes (Fig. 7). It was observed that any of these genes were not present on chromosome numbers 1, 9 and 10. All the five *CsDCLs* were found to be present on separate chromosomes, whereas gene pairs like *CsRDR5a/CsRDR5b*, *CsRDR1a/CsRDR1b* and *CsRDR6b/CsRDR6c* exhibited the presence of these genes in close vicinity with each other. Similarly, presence of homologous genes on the same location was also observed in case *CsAGO* genes, such as homologous pair of *CsAGO2/3e* and *CsAGO2/3f* and close location of *CsAGO2/3a*, *CsAGO2/3b* and *CsAGO2/3c*. This suggests that these genes might have evolved as a result of tandem duplication, thus giving rise to homologous genes. Tandem duplication events are often considered as a major driving force for the evolution of novel biological functions.

### Expression analysis of *AGO, DCL* and *RDR* genes in different parts of tea plant

**Figure 8:**
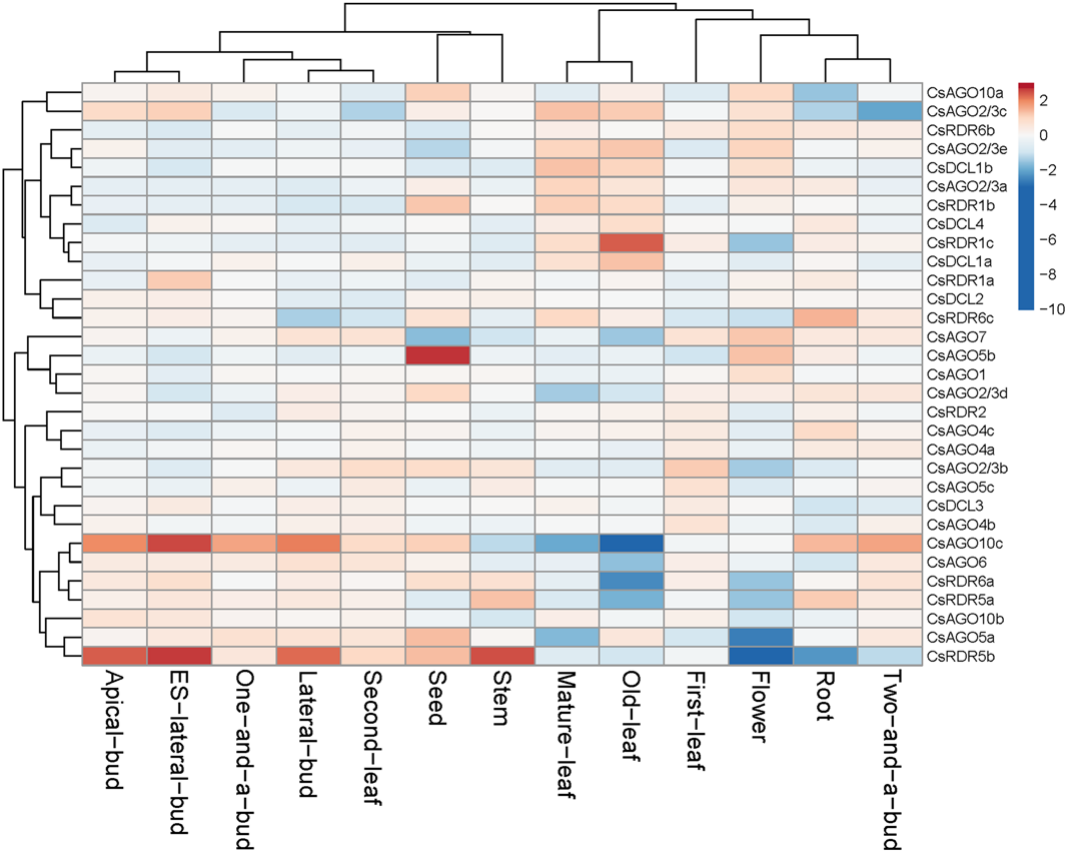
Normalized expression profiles of *AGO, DCL* and *RDR* genes of *C. sinensis* in different plant parts (left to right—apical bud, early-stage lateral bud, one leaf and a bud, lateral bud, second leaf, seed, stem, mature leaf, old leaf, first leaf, flower, root, two leaves and a bud).

To get a perception of the steady-state expression of *CsAGO, CsDCL* and *CsRDR* genes, the transcriptomic RNA-seq data was utilized from a bioproject that had been deposited previously in NCBI Genbank with the accession number PRJNA230752. The generated RNA-seq data included transcriptome profiles of thirteen different tissue samples of tea plant^26^. The final expression data of the *AGO, DCL* and *RDR* genes obtained after analysis were log transformed and illustrated in a heatmap (Fig. 8). *CsAGO10c*, *CsAGO5b*, *CsRDR1c* and *CsRDR5b* showed relatively distinctive expression patterns as compared to all the other analysed genes. This is because of the significant differences in their level of accumulation in different tissues. *CsAGO10c* exhibits the most noticeable tissue specificity within the *AGO* gene family as it gets highly expressed in the buds such as apical bud and both early stage and later stage lateral buds. The expression level of this gene is also seen to be relatively high in agronomically important young tissues like one leaf and a bud and two leaves and a bud. In contrast, the expression level of *CsAGO10c* falls drastically in mature structures like old-leaf, mature leaf and stem. Such contrasting expression measures can also be seen to some extent in *CsAGO5a* which shows extremely low build-up in mature leaf and flower compared to other tissues. Transcript of *CsAGO2/3f* has not been detected in any of the tissues analysed in this project. Most of the *CsRDR* genes show varied expression levels in different tissues, with the most diverse array displayed by *CsRDR5b*. This gene is highly expressed in tissues like apical bud and lateral buds whereas on the other hand its expression falls drastically in flower and root tissues. Regarding the *DCL* gene family all the *CsDCL* genes show a relatively average expression level in all the analysed tissues with no clear distinguishable differences. Genes involved in sRNA biogenesis show maximum variability in their expression in the old-leaf as compared to other tissues. *CsRDR1c* is showed higher expression in the old-leaf tissue, while *CsAGO10c*, *CsAGO6*, *CsRDR6a* and *CsRDR5a* exhibit reduced expression.

### Expression of *CsAGO*, *CsDCL* and *CsRDR* genes during biotic stress conditions

**Figure 9:**
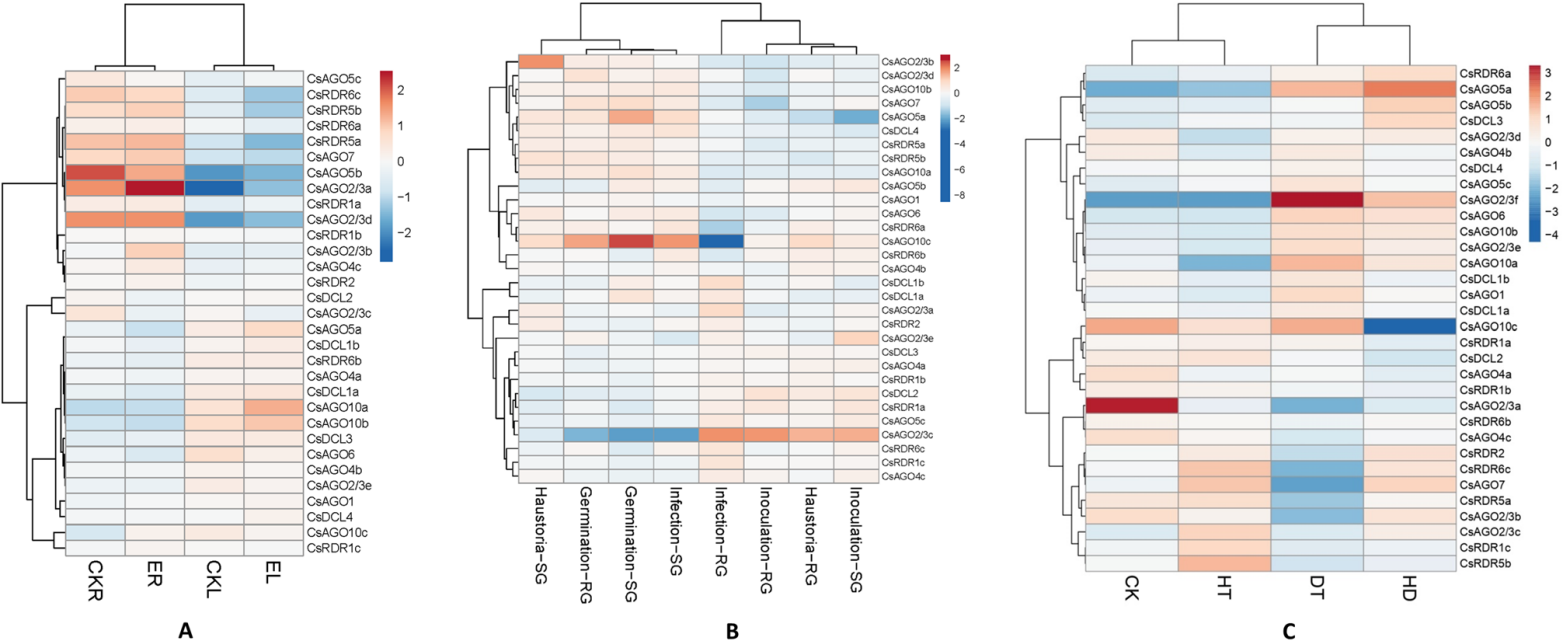
(**A**) Normalized expression profiles of *AGO, DCL* and *RDR* genes of *C. sinensis* in roots and leaves upon *E. oblique* infection (ER and EL) and non-infected plant (CKR and CKL). (**B**) Normalized expression profiles of *AGO, DCL* and *RDR* genes of *C. sinensis* during different stages of blister blight disease in susceptible (SG) and resistant (RG) genotypes. Four distinct stages of infection as depicted are as follows—Spore inoculation (Inoculation); Spore germination (Germination); Haustorial development (Haustoria); Sporulation and secondary infection (Infection). (**C**) Normalized expression profiles of *AGO, DCL* and *RDR* genes of *C. sinensis* under different conditions of abiotic stress (CK- control; HT- high temperature; DT- drought; HD- high temperature and drought).

To envisage an overview of the differential gene expression pattern of *AGO, DCL* and *RDR* genes of *C.* *sinensis* in case of biotic stress, RNAseq data from two publicly available bioprojects were analysed. The generated transcriptomic data for the first bioproject (accession no. PRJNA439206) included the expression profiles of tea leaves and roots upon infection by *Ectropis oblique*^27^. In general, the expression contours of most of the genes were different in leaves and roots, for both infected and control tissues. For instance, *CsAGO2/3a*, *CsAGO2/3d*, *CsAGO5b*, *CsAGO7*, *CsRDR6a*, *CsRDR6c* and both paralogs of *CsRDR5* were up-regulated in roots and down-regulated in leaves. However, when infected tissues were compared with the control samples, some changes were seen in expression levels of particular genes. *CsAGO2/3a* was highly expressed in the infected root sample as compared to the control. Correspondingly the expression level of *CsAGO2/3a* was also higher in *E. oblique* infected leaves than in the uninfected leaf sample. Another gene *CsAGO5b* shows greater expression levels in roots of uninfected plants than that of infected plant roots (Fig. 9A).

In a second bioproject (accession no. PRJNA306068), which has been used for analysing the expression of identified genes, RNAseq data was generated for tolerant and susceptible genotypes during blister blight disease development at four different stages of infection^28^. The most drastic differential expression was found in case of *CsAGO10c* and *CsAGO2/3c*. Out of all the analysed genes, *CsAGO10c* has the highest expression level in spore germination stage in the susceptible genotype and the lowest expression in the sporulation and secondary infection stage of the resistant genotype. *CsAGO2/3c* exhibits an expression pattern that is in contrast with that of *CsAGO10c* since expression of *CsAGO2/3c* is up-regulated in inoculation stage and down-regulated in the germination stage for both the genotypes (Fig. 9B). Most of the other genes however, exhibit average levels of expression changes in different infection stages.

### Expression analysis of *CsAGO*, *CsDCL* and *CsRDR* genes in response to heat and drought stress

Differential expression trends of the members of aforesaid gene families were also analysed for a dataset associated with high temperature and drought treatments (accession no. PRJNA545401)^29^. Transcriptome data was used to generate the expression profiles of the *CsAGOs, CsDCLs* and *CsRDRs* in high temperature (HT), drought (DT), high temperature + drought (HD) and control (CK) conditions (Fig. 9C). *CsAGO2/3f* can be predicted to be an important drought responsive gene, since it was highly upregulated in the drought conditions as compared to control and HT treated samples. More specifically, expression level of *CsAGO2/3f* was even more in DT than in HD treated samples. Similarly, *CsAGO5a* shows increased expression in response to drought as its expression has been seen to be upregulated in DT and HD conditions. On the other hand, *CsAGO2/3a* exhibited a negative correlation with the onset of drought and showed downregulation in drought stress conditions. Furthermore, *CsAGO10c* has been found as one of the genes with significant level of differential expression and was considerably down-regulated in plants treated with simultaneous exposure of high temperature and drought.

### Co-expression network analysis

**Figure 10:**
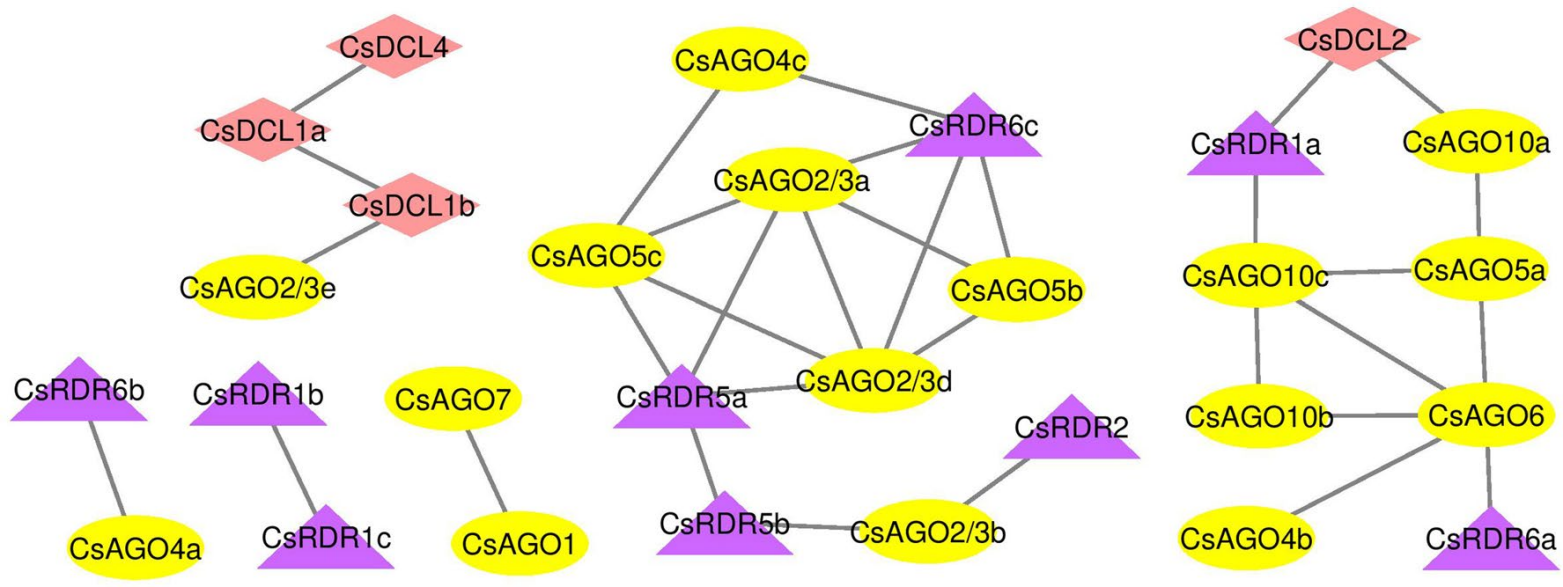
Co-expression networks of *CsAGO, CsDCL* and *CsRDR* genes showing positive correlation, based on combined expression data from various tissues and stress conditions.

To further understand the correlation among the *AGO, RDR* and *DCL* genes in terms of their expression, a positive correlation network analysis was carried out using the RNAseq data (Fig. 10). The co-expression network was constructed with Pearson’s correlation coefficient threshold of 0.5. Three gene pairs viz. *CsAGO1/CsAGO7*, *CsRDR6b/CsAGO4a* and *CsRDR1b/CsRDR1c* are found to be interacting and co-expressing independently. Exhibition of linear correlation was observed in expression patterns of *CsDCL4, CsDCL1a, CsDCL1b* and *CsAGO2/3e*. Two more conspicuous networks can be seen which show intensive cross-links among various genes. Besides, in some cases gene members belonging to the same phylogenetic clade have been found to be present in the same network with close relationship in their expression. These results suggest that various combinations of *AGO*, *DCL* and *RDR* may participate in different RNA interference pathways in *C. sinensis.*

### qRT-PCR based expression analysis of *CsAGO, CsDCL* and *CsRDR* genes in various tissues

**Figure 11:**
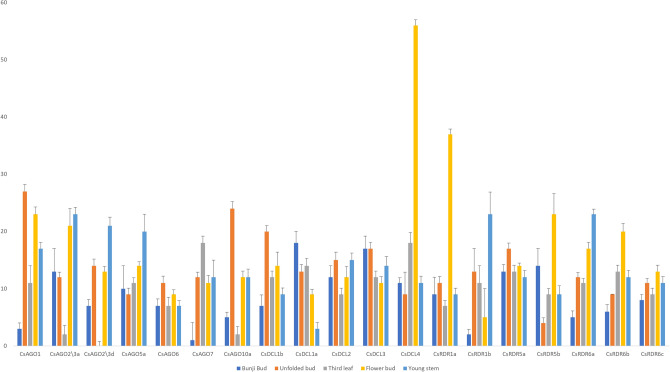
qRT-PCR analysis showing the results of expression pattern of 19 considered genes in different tissues of TV1 cultivar of tea plant. The names of the genes are shown in the x-axis, and y-axis represents the fold changes of expression of the genes.

To validate the expression of *AGO, DCL* and *RDR* genes in various tissues in tea, we analysed the expression profiles of 7 *CsAGOs*, 5 *CsDCLs* and 7 *CsRDRs* by qRT-PCR, which were randomly selected from the total set of 32 genes. Since *CsAGO2/3f* exhibited interesting expression patterns in the high throughput analysis showing particularly drought-specific trends, primers were designed for this gene based on its CDS. However, even after repeated trials, *CsAGO2/3f* did not show any amplification in any of the analysed tissues. Five different tissues were considered for this analysis, viz. bunji bud, unopened bud, third leaf, flower bud and young stem of TV1 plants, which is a popular Indian tea cultivar. As shown in Fig. 11, the 19 analysed genes showed variable expression in five different tissues. *CsAGO6, CsDCL2, CsDCL3, CsRDR5a* and *CsRDR6c* did not show significant variation in their expression levels in different tissues, the pattern of which is mostly similar to the expression levels detected for these genes in the SRA data analysis (Fig. 8). Among rest of the genes, *CsAGO1*, *CsAGO10a* and *CsDCL1b* showed comparatively highest expression in unfolded apical buds relative to other analysed genes. The expression levels of *CsDCL4* and *CsRDR1a* were remarkably higher in flower buds with respect to other tissues. *CsDCL1a* showed downregulation in all the other tissues with respect to bunji bud, whereas *CsAGO1*, *CsAGO7* and *CsRDR1b* were expressed least in bunji bud. *CsAGO7* was found to express more in third leaf as compared to other tissues, whereas *CsAGO2/3a*, *CsAGO2/3d*, and *CsAGO10a* exhibited minimal expression in third leaf tissue. Among all the analysed genes, a higher expression level in stem was displayed by *CsAGO2/3a, CsRDR1b* and *CsRDR6a*, whereas *CsDCL1a* had lowest expression in stem.

## Discussion

RNA interference is an adaptable phenomenon that regulates the degree of accumulation of gene transcripts by sequence specific gene silencing machinery. Thus exploring the differential expression patterns of the core genes of this silencing machinery in different conditions becomes indispensable. Coordinated function of *RDR-DCL-AGO* genes is crucial for processing different classes of small RNAs, which indirectly makes them involved in regulation of diverse biological pathways^30,31^. Members of these three gene families are involved in biogenesis of sRNAs and effective silencing of their targets. For example, *DCL1* is primarily involved in the biogenesis of microRNAs with no necessity of RDR proteins, whereas *DCL2*, *3* and *4* are mainly responsible for processing of siRNAs originating from long dsRNAs synthesized by the action of RDR proteins^32,33^. Moreover, *DCL3* and *DCL4* products are also known to have discrete functions, with the former known to be involved in RNA-directed-DNA-methylation (RdDM) pathway and latter being a component of the RNA interference apparatus^34^. In regard to the *AGO* gene family, *AGO1* is generally the most prevalent member engaged in miRNA mediated gene silencing process. However in some cases other homologs of *AGO* genes also participate in completing the silencing machinery of various miRNAs. For example, miR390-*AGO7* module is involved in the regulation of Auxin Response Factors (ARFs) and miR166-*AGO10* module has been reported in development of shoot apical meristem^35^. Three gene families—*AGOs*, *DCLs* and *RDRs*—which function as the key components for biogenesis and action of the sRNAs in tea plants were identified in this study. Eighteen *AGOs*, five *DCLs* and nine *RDRs* were predicted in this study from tea plants with the number of genes being significantly higher than that in *A. thaliana*. However this increase in number of genes might be attributed to more number of chromosomes and bigger genome size of tea^25^. The expression pattern of the genes was also analysed in different tissues of the tea plant and also in response to biotic and abiotic stresses.

### Structural organisation and gene expansion

The domain analysis in the CsAGO family revealed the presence of N-terminal domain, PAZ, Piwi, Mid domains and linker-1 and linker-2 domains. However, only the ArgoN, PAZ, Piwi and Argo-L2 domains were found to be present in all the identified AGOs whereas the Mid domain was absent in CsAGO7, CsAGO4c and five members of CsAGO2/3 clade except CsAGO2/3d. Linker-1 domain was absent only in CsAGO4a. A Glycine-rich-AGO1 specific domain was found in CsAGO1, which has already been reported in AGO1 proteins of many other plants such as *Arabidopsis* and *Coffea*^36,37^. In the DCL family CsDCL1a, CsDCL2, and CsDCL3 showed the presence of N-terminal and C-terminal domains of DEAD-box helicase, Dicer-dimerization domain, PAZ and RNase III domains. CsDCL1b however lacked the DEAD-box helicase and the Dicer-dimerization domains, whereas PAZ domain was absent in CsDCL4. A dsRNA binding motif was also detected in the protein sequences of CsDCL1a and CsDCL1b. The CsRDR proteins consisted of one conserved domain i.e. RdRP, which is truncated to some extent in CsRDR5a and CsRDR5b. Furthermore, a part of RNA recognition motif superfamily (RRM_SF) is present towards the upstream region of CsRDR2 protein. Presence of RRM_SF has also been reported in RDR2 protein of *Salvia miltiorrhiza*^38^. The gene copy numbers are greater than those of *A. thaliana* indicating that the genes might have undergone significant expansion through gene duplication events. Selection pressures leading to large-scale duplication events have also been observed for a number of stress responsive genes in tea^22,39^. Expansion of *AGO* and *RDR* gene families suggests a corresponding diversification of the gene function in tea^40^. This is also substantiated by tandem duplication events observed for homologous genes of *CsRDRs* and *CsAGOs*, which are present on the same chromosomal location.

### *AGOs*,* DCLs* and *RDRs* as moderators of gene silencing

Different set of genes are regulated in distinct developmental stages of a plant in a tissue specific manner. *CsAGO1* seems to be the most ubiquitously expressed gene in all the tissues and various stress conditions considered in this study. It is one of the main components of gene silencing machinery and is known to participate in the biogenesis of most of the conserved miRNAs and siRNAs in tea^24^. In our study *CsAGO1* seems to be ubiquitously expressed in all the tissues and various stress conditions in tea. As stated earlier, *CsAGO10c* has been found to be the most dynamically regulated *AGO* gene according to the nature of the plant tissue in *C. sinensis*, and shows high accumulation in buds. Activity of miR166-*AGO10* module is important for meristem formation in plants^35^. Expression of *CsAGO10c* is found to be higher in tissues undergoing active meristematic development such as buds, roots and seeds. Furthermore, *CsAGO10c* also exhibits significant potential in supplementing the establishment of *Exobasidium vexans* infection in tea to cause blister blight disease. The level of accumulation of *CsAGO10c* seems to be quite similar during inoculation, germination and haustorial development stages in both the resistant and susceptible genotypes. However at the final stage of sporulation and secondary infection, the expression of *CsAGO10c* is highly down-regulated in the resistant genotype, as compared to the susceptible one. During the *E. oblique* infestation in tea, expression levels of *CsAGO2/3a*, *b* and *d* were higher in effected roots and leaves as compared to control. On a similar trend, expression of *AGO2* has been reported to be significantly induced by biotic stress in *Capsicum annuum*^41^. This indicates that *AGO2* might be somehow involved in regulation of defense mechanisms in plants against pathogens. In case of drought stress in tea, expression level of *CsAGO2/3a* is reduced significantly compared to control. Interestingly, the 3′UTR of *AGO2* has been validated as a target site for a drought responsive miRNA i.e. miR403 in *Arabidopsis*^42^. Downregulation of *CsAGO2/3a* in tea during drought and heat stress may result from any such interaction with a stress-induced miRNA, which warrants further investigation. In *Arabidopsis*, *AGO2* and *AGO3* have been reported to play substantial roles in antiviral defence and epigenetic pathway respectively, and both these genes show high amount of homology in their protein sequences^43^. *CsAGO5a* exhibits preferential accumulation in seed, while *CsAGO5b* mostly accumulates in both seed and flower which could be a probable result of active involvement of *CsAGO5* in reproductive tissues. Higher expression of *AGO5* has also been reported in *Arabidopsis* during all stages of flower and seed formation^44^.

*DCL* genes are important components for biogenesis of miRNAs and various classes of siRNAs. Even though plants have evolved four different groups of *DCLs*, these are said to have structurally diversified with overlapping functions^45^. The *DCL* genes in tea seemed to exhibit a more or less uniform expression levels in all the tissues, with the only notable observation being slightly higher expression of *CsDCL1b* in mature and old leaves and in flower. Potential role of *DCL1* genes in inducing flowering has also been suggested earlier in *Arabidopsis* where *dcl1*/*dcl3* mutants exhibited delay in flowering^46^.

*RDRs* participate in dsRNA synthesis for the biogenesis of siRNAs. We identified nine *RDRs* in our study representing four different homologous groups viz. *RDR1*, *2*, *5* and *6*. Expression of *CsRDR* genes showed significant degree of variability in different tissues and stress conditions. *RDR2* has been reported to be actively involved in biogenesis of hc-siRNA, along with participation of *AGO4* in the DNA methylation pathway in *Arabidopsis*^47^. In our study, expression levels of *CsAGO4a* and *CsAGO4c* are mostly similar to *CsRDR2* in all the considered tissues of tea plant. *CsRDR5a*, *CsRDR5b* and *CsRDR6c* showed low expression levels in *E. oblique* infested samples, but were also found to be tissue specific showing significant differences in their expression. This suggests they might play critical roles under definite circumstances in plant growth and development or that their expression could be induced in response to specific environmental signals and during various stress conditions. The qRT-PCR results also showed variable expression patterns of the *AGO*, *DCL* and *RDR* genes in different tissues.

Functions of AGO, DCL and RDR proteins as components of silencing machinery in inducing resistance against abiotic and biotic stress has been studied extensively in various plants^14,41,48^. Moreover, miRNA mediated gene silencing is a crucial regulatory process of important agronomic traits of various crops. Hence, comprehensive knowledge about the regulatory potential of these three components of gene silencing machinery becomes important in the aspect of genetic improvement of an economically important crop such as tea.

## Conclusion

Functional association between *AGOs*, *DCLs* and *RDRs* is responsible for supplementing gene regulatory functions like RNA interference and RdDM in eukaryotes. Evaluating the potential roles of these important gene families in a commercially important crop like tea certainly helps to engineer tea crop to enhance crop productivity and quality. In the present study, we have identified 18 *AGOs*, 5 *DCLs* and 9 *RDRs* in tea genome*.* Phylogenetic and structural analyses of these gene sequences show differences in arrangement of exons and introns, based on which they can be grouped into distinct clades. Even though the identified genes exhibit evolutionary expansion in tea, their expression patterns in various tissues and stress conditions indicate presence of overlapping functions among the paralogous members. Presence of stress hormone related promoter elements in their upstream region indicates the involvement of these genes in adaptation during stress condition in tea. The genes identified in this study can be used as potential targets for crop improvement for developing stress resistant tea cultivars.

## Materials and methods

### Identification of *CsAGO*, *CsDCL* and *CsRDR* gene family members

The reference genome, coding sequences (CDS) and peptide sequences of *C. sinensis* var. *sinensis* were downloaded for Tea Plant Information Archive (TPIA). In order to identify the *AGO, DCL* and *RDR* gene families, the alignment files of the PIWI, PAZ, RNaseIII and RdRP domains were downloaded from pfam database in Stockholm format from which the corresponding HMM profiles were built using the HMMER toolkit^49,50^. The tea peptide sequences were then searched for the presence of HMM-profiles of the conserved domains followed by subjecting the identified non-redundant proteins to domain analysis in batch CD search against the pfam and SMART databases with default cut-off parameters^51^. Peptide sequences of *C. sinensis* were also BLASTP searched against AGO, DCL and RDR protein sequences of *A. thaliana* to ensure that any putative genes of these three gene families are not left out from the analysis. Sequences containing N-terminal (pfam16486), PAZ (pfam02170) and PIWI (pfam02171) domains were recognized as AGO proteins. Linker and Mid domains however may or may not be present in all the identified genes. Similarly, tea peptides showing presence of RNase III domains were analysed in batch CD search against pfam and SMART database to detect the presence of all the conserved domains of DCL proteins viz., DEAD (pfam00270), Helicase-C (pfam00271), Dicer-dimer (pfam03368), PAZ (pfam02170), RNaseIII (pfam00636) and DSRM (pfam00035). For identification of *RDRs*, the peptides which exhibited the presence of RdRP domain were considered as putative RDRs of tea. The positions and structural integrity of the identified domains were also confirmed by biosequence analysis using Hidden Markov Models in HMMER database^52^. The identified genes were named according to their positions in phylogenetic trees which also included designated *AGOs*, *DCLs* and *RDRs* of *A. thaliana*^53^.

### Characterization and physicochemical properties

Amino acid properties, physicochemical traits such as charge, molecular weight (g mol^−1^), aliphatic index, instability index (II), isoelectric point (pI), grand average of hydropathy (GRAVY) and other properties of the CsAGO, CsDCL and CsRDR proteins were calculated using the ProtParam tool in the ExPASy web server^54^.

### Sequence alignments and phylogenetic analysis

Multiple sequence alignments for the predicted CsAGO, CsDCL and CsRDR proteins were performed using ClustalX 2.1 program with default settings, and viewed using GeneDoc software v1.0 (https://genedoc.software.informer.com)^55,56^. The identified conserved domain sites specific for AGO, DCL and RDR were manually checked and verified using the coordinates’ data of the conserved domains in each protein, obtained using the ‘hmmscan’ tool from the HMMER web server^50^. MEGA7 software (https://www.megasoftware.net) was used to carry out the evolutionary and phylogenetic analyses^57^. Preliminary trees for heuristic search were obtained by applying Neighbour Joining/BioNJ method to a matrix of pairwise distances estimated using Jones-Taylor-Tshorton (JTT) matrix-based model^58^. Final phylogenetic trees were constructed using Maximum Likelihood method based on the Jones-Taylor-Thorton (JTT) model using bootstrap of 1000 replicates. The trees were squared to scale, with number of substitutions per site represented by branch lengths. The phylogenetic analyses also included putative orthologous genes from other plant species, which were BLASTP searched and downloaded from Phytozome^59^ using *CsAGO1, CsDCL1a* and *CsRDR1a* encoded proteins as query. Neighbour Joining (NJ) trees using 5000 bootstrap replicates and JTT based model were constructed using the identified orthologs.

### Prediction of gene structure, motifs and miRNA target sites

The structures of the *AGO*, *DCL* and *RDR* family genes showing exon–intron organization were determined based on alignments of their coding sequences with the corresponding genomic sequences, and an illustration was obtained using Gene Structure Display Server 2.0^60^. The conserved motifs in the identified proteins were identified in MEME web server keeping the optimal motif width between 6 and 200, and the maximum number of different motifs as ten^61^. The discovered motifs were annotated with Pfam and hmmscan programs^49,50^. For miRNA target sites prediction within the *CsAGO*, *CsDCL* and *CsRDR* transcripts, sequences of identified transcripts were used as target gene input to the psRNATarget server^62^ and analysed against all the available plant miRNAs using an expect value threshold of 2.0 and maximum energy to un-pair the target site (UPE) up to 50 units.

### Identification of *cis-*acting regulatory elements, chromosomal location and dn/ds calculation

The data about locations of the identified genes in different scaffolds of the genome were obtained from TPIA platform and 2000 bases upstream sequences were retrieved. The *cis-*acting elements present in these upstream promoter regions of *AGO*, *DCL* and *RDR* genes of *C. sinensis* were identified using PLANT CARE database^63^.To gather information about the chromosomal locations of each gene, the pseudo-chromosome sequences of tea genome available in TPIA, and the gene sequences were blasted, following which coordinates of each gene on the chromosomes were depicted on physical map of each chromosome using Mapchart v2.3^64^.

Orthologous genes of the *CsAGOs, CsDCLs* and *CsRDRs* were identified in *A. thaliana* by using BLAST tool of Phytozome^59^. The best hit for each of the genes were designated as orthologous partners and rates of synonymous and non-synonymous substitutions were determined using the PAL2NAL utility^65^. The dn/ds ratio was calculated in order to assess the selection history and divergence time of the gene families. The divergence time (T) was calculated using the formula T = ds/(2λ) × 10^−6^ million years ago (MYA), where value of λ = 6.5 × 10^–9^ (Universal substitution rate)^66,67^. The pairwise alignment files for the protein sequences required as inputs in the PAL2NAL program were created using Clustal Omega^68^.

### Analysis of *AGO, DCL* and *RDR* gene expression in tea

Transcriptome data from four different bioprojects submitted in NCBI were downloaded for in silico analysis of expression data of *CsAGO, CsDCL* and *CsRDR* genes. The details of the bioprojects are as follows: (i) To explore the expression patterns of these genes in different tissues of tea plant, the Illumina RNA-sequencing data of *C. sinensis* (L.) O. Kuntze cv. ‘*Longjing 43*’ was downloaded from GenBank archives (accession no. PRJNA230752)^26^. The SRA data of 13 different tissue samples viz. apical bud, early stage lateral bud, lateral bud, flower, seed, stem, root, mature leaf, old leaf, two and a bud, one and a bud, first leaf and second leaf were downloaded, from which the low quality reads and adapters were removed, and then mapped to the tea reference genome^22^. (ii) Data obtained from the bioproject with accession no. PRJNA439206 includes transcriptome profiles of leaves and roots of *E. oblique* infested plants along with the control samples^27^. (iii) The third dataset with accession no. PRJNA306068 represents blister blight infected leaf samples at four different stages of infection viz., spore inoculation, germination, haustoria development, and sporulation and secondary infection for both susceptible and resistant genotypes of tea^28^. (iv) Differential expression analysis of the said genes was also carried out for a particular study associated with abiotic stress, i.e. bioproject PRJNA545401. This dataset includes the RNA seq data for tea plants treated with high temperature and drought conditions^29^. An annotation file consisting of only the identified *AGO*, *DCL* and *RDR* genes with their respective gene-IDs was manually created to get the mapping and expression data of only these genes. The gene expression data was normalized by FPKM (fragments per kilobase per million) and the resulting FPKM values of genes were log2 transformed using edgeR and Trinity (R language-based) programs. The dispersion value threshold was set as 0.1, as the samples analysed were tissues belonging to same cultivar of tea and were highly similar in their genetic constitution. The heatmaps along with the expression clustering were generated and visualized using Clustvis—an R based online tool (https://biit.cs.ut.ee/clustvis/)^69^.

### Gene co-expression network construction

To represent the co-expression profiles of the identified genes, we performed gene co-expression network analysis using the FPKM data generated for the gene expression evaluation using RNA-seq data. Cytoscape software version 3.7.2 (https://cytoscape.org) was used for this purpose where FPKM matrices of the gene expression were fed as inputs^70^.

### qPCR validation of selected transcripts in tissues representing different developmental stages

To determine the expression of some of the members from the 3 different gene families, qRT-PCR analysis was carried out using the different tissue samples collected from young saplings of TV1 cultivar. Around 100 mg tissue was used to extract total RNA with Trizol reagent following manufacturer’s protocol (Invitrogen, USA). Quantity and quality of the purified DNA-free RNAs were determined using Nanodrop 1000 (Thermo scientific, USA) and agarose gel electrophoresis. The cDNA was prepared by using 2 μg of total extracted RNA using SuperScript III cDNA synthesis kit (Invitrogen, USA) following manufacturer’s protocol. The cDNA samples were diluted 40 times and then subjected to qRT-PCR. The diluted cDNAs were used for 25 μl PCR reactions using QuantiFast SYBR Green PCR Master Mix (Qiagen, India). The gene specific primers were designed manually for all the transcripts along with 18S rRNA (NCBI Genbank id: AF207029.1) as an internal control^71^. The primers used in this study are listed in Supplementary Table S4. Real-time PCR analysis was conducted as described previously^72^, using primer specific annealing temperatures. Two technical replicates from three individual biological replicates were considered for each experiment conducted. The relative expression analyses of the qRT-PCR results were expressed using the 2^−ΔΔCT^ method^73^. Five different tissues viz. bunji bud, unfolded bud, young third leaf, unopened flower bud, and young stem of TV1 plants were considered to analyse the relative expression levels in various tissues.

### Ethical approval

The authors have obtained permission to collect tea plant material for the experiment. The authors also declare that the experimental research work conducted in this study comply with relevant institutional, national, and international guidelines and legislation.

## Supplementary Information

Supplementary Information.
