# Supplementary material for: Genome-wide identification, evolutionary relationship and expression analysis of AGO, DCL and RDR family genes in tea
Source: Sci Rep. 2021 Apr 21;11:8679. doi: 10.1038/s41598-021-87991-5 (PMC8060290; doi:10.1038/s41598-021-87991-5)
Supplement: Supplementary file 1 — Supplementary Information. [file 41598_2021_87991_MOESM1_ESM.pdf]

Genome-wide identification, evolutionary relationship and expression analysis of AGO, DCL and RDR family genes in tea

Debasish B. Krishnatreya[1], Pooja Moni Baruah[1], Bhaskar Dowarah[1], Soni Chowrasia[2], Tapan Kumar Mondal[2], Niraj Agarwala[1]*

1. Department of Botany, Gauhati University, Jalukbari, Guwahati, Assam, 781014, India.

2. ICAR-National Institute for Plant Biotechnology, LBS building, Pusa, IARI, New Delhi-110012, India.

*Correspondence to: niraj_botany@gauhati.ac.in

Supplementary Figure S1-a. Multiple Sequence alignment of CsAGO proteins: The names of the identified CsAGO genes are indicated to the left of the alignments. The length of the alignment is shown on the top, while the length of each protein is given on the right. Conserved consensus residues are shown at the bottom. Invariable residues conserved at 100% are highlighted in red, those conserved at >80% are shown in yellow and residues that are >60% conserved are highlighted in green. The sequences were aligned usi9ng ClustalX and viewed by GeneDoc.

```
                      *        20         *        40         *        60         *
CsAGO2/3f : ---------------------MNPHEHLETLVHLHCP-----LFIQDCPLLSLNSQTP----------TQSR :  36
CsAGO2/3d : ------------------------------------------------------------------------ :   -
CsAGO7    : --------------------------------------------MEETEEANTTKKCTP------------- :  15
CsAGO10a  : ------------------------------------------------------------------------ :   -
CsAGO2/3a : --------------------------------------------MERDGGRNYNRGRGGAAPPRSGGD---- :  24
CsAGO2/3b : --------------------------------------------MERDGGRNYNRGRGGAALPRSGGDGGNR :  28
CsAGO6    : ------------------------------------------------------------------------ :   -
CsAGO4c   : ------------------------------------------------------------------------ :   -
CsAGO4a   : ------------------------------------------------------------------------ :   -
CsAGO4b   : ------------------------------------------------------------------------ :   -
CsAGO2/3c : --------------------------------------------MERDG-RNYNRG-----APRGASCG--- :  19
CsAGO10b  : ------------------------------------------------------------------------ :   -
CsAGO10c  : ------------------------------------------------------------------------ :   -
CsAGO1    : MFGKEDEKRRKRGRKLLVEADLAALAYLTQDGKWYLKSGFEIYILLASLYSFSLGSIAISGFSLLPGSENGF :  72
CsAGO5c   : ---------------------MKGLRLWLGEREDDKNKGFRFRSPARERRGGWWSVVVGVATAVVIAREERD :  51
CsAGO5a   : ------------------------------------------------------------------------ :   -
CsAGO5b   : ------------------------------------------------------------------------ :   -
CsAGO2/3e : ---------------------MGYDDYIEKKVEVVSNPCDEVGFIHPSQFVMLNEEAGGPLPSSVGTMLQSE :  51

                  80         *       100         *       120         *       140
CsAGO2/3f : FHGLNALESEEEPTTADELLMVVVVAVVTEEFLSFS----------LPRYQTHNHHHHQYHGGGQGGGGTYT :  98
CsAGO2/3d : --------------------------------------------------MERSNYGRGGRGGGGGRGGRGG :  22
CsAGO7    : -----KTRSFRGRNSPP------------------------------NHHHHHHHYQQQQQQQLLLQ----- :  47
CsAGO10a  : --------MPMRQMKEIP-------------------EQHLVIKPQLQNSMNQHLVIKPQLQNSMNQVHK-- :  43
CsAGO2/3a : --RGGNGGGGRGRGQGGGGRGGARAWGPSEQSQNQGQ--GGGGGRGGGARAWGPPQQGQNQGGGGGGGG--- :  89
CsAGO2/3b : GGRGGNGGGGRGRGQGGGGRGGARAWGPSELSQNQGQCGGGGGGRGGGARAWAPPQQGQNQGGGGGGGGGRGG : 100
CsAGO6    : ------------------------------------------------------------------------ :   -
CsAGO4c   : ------------------------------------------------------------------------ :   -
CsAGO4a   : ------------------------------------------------------------------------ :   -
CsAGO4b   : --------------------------------MMIMMATVTMAATAEGVAVKFPFMKAINPNLTLPNRTS-- :  38
CsAGO2/3c : ---GGGSGSGRGRGRG---------------------------------RAWGPSQQGQNQGGGRGQ----- :  50
CsAGO10b  : --------MPMRQMKESS-------------------EQHLVIKPQLQN--------------TMNQAQK-- :  29
CsAGO10c  : --------MPHWQPKEPE-------------------QRHVISRRPLQD--------------SMNQHSL-- :  29
CsAGO1    : PVRASGIIMVRKRRTELPGGGESSESQEASGGRGGGAQRPPAQQQQQQQQQGGGHQGGGRGWGPQSQQGGRG : 144
CsAGO5c   : EKKYSRRRTEREREREREGGGRHG------------VQSSEMSTRGRGR----GDGGGRGRGRGQPRRDQ-- : 105
CsAGO5a   : -----------------------------------------MSGRGRGR----GRG-----GRGQPRHDR-- :  20
CsAGO5b   : -------------------------------------MSGRNRGRGSGV----GGGDGGGRGRAQPRTGL-- :  29
CsAGO2/3e : DRVLGSRVSKVNPSETVFWCRDHKLGISTQVLLPLYNVAKQTFMAALERYKIHSNLSVKKDESGSDNMSSWY : 123

                *       160         *       180         *       200         *
CsAGO2/3f : AT-----------------------------LQPPQPPPQQWGPRPVQGQRGPYMRPPPR------------ : 129
CsAGO2/3d : GG----------------------------------------------GGRGSVDR---------------- :  32
CsAGO7    : -------------------------------------QSNRIGYCFNQNQYQTYPALLPLP----------- :  71
CsAGO10a  : ---------------------------PPKTAQNGKGPPTHESQNTELHNQPFLPSKNRGR----------- :  77
CsAGO2/3a : --------------------------------------ARGWGPSQSGGQPWVDRAVQGGPVGGGGGGACRP : 123
CsAGO2/3b : GG------------------------------------ARAWGPSQSGGQQWVDRAVQGGPVGGGGG-AYRP : 135
CsAGO6    : ----------------------------------------------MEKEAVGSRRLPPPP----------- :  15
CsAGO4c   : -------------------------------------------MDTFEKDGNGTTESLPPP----------- :  18
CsAGO4a   : -------------------------------------------MEPFG----ETDVLLPPP----------- :  14
CsAGO4b   : ----------------------ITILVLMYSFSVGSMALSFDLMDSFEHDGNGATEALPPP----------- :  77
CsAGO2/3c : --------------------------------------VRAWGPSQSGGQQWGP------------------ :  66
CsAGO10b  : ---------------------------PPKTAQNGKGPPTQEPQNTKIYNQTSPPSKNRGR----------- :  63
CsAGO10c  : ---------------------------SQESSEDAN----------------KIEGSGRGR----------- :  47
CsAGO1    : GYGGRGGGGGGGGPQRGGMAGQQYYGGPPEYHQQGRGGPPRGGVSQQRRGGSSSSGVGGGLGVVPSA---GG : 213
CsAGO5c   : --------------------------PPPPSSSGRGGGRGRGTAGGSAPYQAAPPPQRPPH----------- : 140
CsAGO5a   : --------------------------PSPSPASSSGGRGGNHHIPPPASSQFPALSSPTPP----------- :  55
CsAGO5b   : --------------------------PSP---AVQGRGRGRGGAG-PAPTQAPPPTVSQPP----------- :  60
CsAGO2/3e : ASSLDVLESEVMKHSRALLLLSCDFGTVWNSSRRLRSPPSAVVAPAAQQQRQSLSRRKPSSSSSNSTVISGP : 195

              220         *       240         *       260         *       280
CsAGO2/3f : VRPTRQDSGEISDRGRGRIPGRRGSGRACQPAP-----APRTQSLSSPEPPPRPR---PDFQILEQPPASSL : 193
CsAGO2/3d : -----QYQGGGGRGGDPSIP-----------------------------------------------DISSL :  52
CsAGO7    : PIMPLQLTVTPPLPQNQTFRSKTHLQKHPWKQNNPPLATSSDPEPQNSAVSPAPEGLGRQPGIPFRGKDGRR : 143
CsAGO10a  : -------RRGKGGRKPDNSDVLMRPSLRPAS--VQPTGAFEADIPNGSRSRSN--------------SCVER : 126
CsAGO2/3a : PAPLQQVQGVGVGPTSGVWSGRPWSG-----QSSSQ--PPIQPVPVPQKLPEQALPDIKELEISEKKPSPAI : 188
CsAGO2/3b : PAPLQQVQGVGVGSTRGVWSGRPWSGSPVAGQSSSQ--PPIQPVPVSQKLTEQALPDIKQLQISEKKPSSSI : 205
CsAGO6    : ---------------------------PIIPPTVKPEKLVTPERS--------------------------- :  33
CsAGO4c   : ---------------------------PPVPADVVPMRA-EADNAPEPVKN--------------------- :  41
CsAGO4a   : ---------------------------PEIPPDIDPIRVQEPEDVPEFTKKTS------------------- :  40
CsAGO4b   : ---------------------------PPVPPNIVPLQA-EPDHAPEPVKK--------------------- : 100
CsAGO2/3c : ----QQGQ--------GVWSGRPWIGQ--SSSSSQT--HQIQTVPVPHRFPEQLLPDVQSLEISEKKPQLSI : 122
CsAGO10b  : -------RRGRGGRKSDQGEVFMRPSSRPCTGVHKPARAGETDIPNGSIVNGAN------------LCEVEM : 116
CsAGO10c  : -------RRSAKGSK------------------METAHNVKPEIFN-----------------------VEP :  71
CsAGO1    : PSRPPVPELHQATQASYQAGVTTQPVAYPKPAEMHGEASSSSPVPDPSLSQVTSKVQGLSLQEEGTSSQAIQ : 285
CsAGO5c   : ------YASTSTPSSSQPPPTPS----SSLSQDVERKLILQSPPPTPAAAQPSSSVQRPA-QPPLPAQDPAQ : 201
CsAGO5a   : ------LASSSASLSRDMQLKLT--LQSPAPAQLTLPPVVQTPPP------------------PQSVKEAVQ : 101
CsAGO5b   : ------VVSHSPPPSQAPSSSTTRTEVSSLSQDVESKLSMQTSPS--------------------PVQPMTV : 106
CsAGO2/3e : TRIVYREALNESSESDLNLPFWQLEGTPTLPAFGCLGKYARGQSLLLNKLETCPFNSIPDFQILEQPPASSL : 267

                *       300         *       320         *       340         *       360
CsAGO2/3f : PR-TKENNLVPVRRPDKGGTHSIQSPKLLVNHFPVMFNAKTTIIHYYIDIKEEVPSKNRSAK--IQKSHLRM : 262
CsAGO2/3d : KI-SSGSNLLPVRRPDTGGTLAIQSPKLSVNHFPVRFNEKTIIIHYDIDIKPDVSSKSRPAR--VSKSVLSM : 121
CsAGO7    : VMGSTPQSLVVVRRPDSGGVEGAVIS-LLANHFLVQFDPSHRIFHYDVEISPNP-----------SKEVARL : 203
CsAGO10a  : GFPSSSKSLSCAHRPGFG--QLGTKCVVKANHFFAEL-PDKDLNQYDVTITPEVAS---------RAVNRAI : 186
CsAGO2/3a : LG-SSGKKVVPMKRPDDGGTLAFRNTRLLANHFPVKFNPQSIIMHYDVDIKPNMPPGSRAAKRPISKSDLRL : 259
CsAGO2/3b : PGGSSEKKIVPIKRPDNGGTLAFRNTRLLANHFPVKFNPQSIIMHYDVDIKPNMPPGSRAAKRPISKSDLRL : 277
CsAGO6    : ----------IISRRGFG--SAGRRISLLTNHFKVSV-KSPNEIFYQYSVSISSEDKRVVEN---KGIGRKI :  89
CsAGO4c   : -----KAVRVPMARRGVG--SKGQKIPILTNHFKVNV-TNVS---------VSYEDGRPVDG---KGAGRRV :  93
CsAGO4a   : -----EPKRVPMARPGTG--SKGQKISLLSNHFKVGI-TNATGHFFHYCVSIFYEDDRPLDG---KGIGRKI : 101
CsAGO4b   : -----KVMRVPMTRRGQG--SKGQKVPLLTNHFKVNV-TNVDGHFFHYSVSVSYEDGRPVDG---KGVGRKV : 161
CsAGO2/3c : PE-SSDKRIVPIRRPDRGGTLAVRSTRLFANHFPVRFNPESTIMHYNVDIKPNTTPGSRAAKRPIPKSDLRL : 193
CsAGO10b  : GFPSSSKSLSFAPRPGYG--QLGTKCVVKANHFFAEL-PDKDLNQYDVTITPEVAS---------RNVNRAI : 176
CsAGO10c  : --PPSNKSLVLHRRPGYG--QLGTKCLVKANHFLAEI-SEGDLTQYSVKIIPEVNS---------TKLNKAI : 129
CsAGO1    : PAPPSSKSMRFPLRPGKG--SSGTRCIVKANHFFAEL-PDKDLHQYDVSITPEVTS---------RGVNRAV : 345
CsAGO5c   : PP-LSTKSLRPPARPGYG--TVGRRCIVRANHFLVDV-ADRDLHHYDVTITPEVTS---------KKVCREI : 260
CsAGO5a   : PPPASSKAVRLPARPGFG--TAGQKCVVRANHFMVS-------------ITPEVIS---------KKLHREI : 149
CsAGO5b   : LP-SSSKSLRPPARPGYG--TIGRKCIVRANHFLVDV-ANKDLHHYDVTITPEVAS---------KKVCRDI : 165
CsAGO2/3e : PR-TKENNLVPVRRPDKGGTHSIQSPKLLVNHFPVMFNAKTTIIHYYIDIKEEVPSKNRSAK--IQKSHLRM : 336
                           Rp  G         6  NHF v                                    6
```

```
                     *       380         *       400         *       420         *
CsAGO2/3f : IKDKLFSDDPSQFPLSSTAYDGDKNIFSAVELPTE-----------KFKVKFSDGEGMKC------------ :  311
CsAGO2/3d : VKDKLFSDDPAQFPLSRTAYDGERNVFSAVPLPTG-----------TYKVDFSGGEDMKS------------ :  170
CsAGO7    : IKQKLVEDNSVVLSGALPVYDGRKNLYSPMEFQSDRLEFFICLPIPSSKSLLPSRESVDSQ----------- :  264
CsAGO10a  : MAELVKLYKESDLGKRLPAYDGKKSLYTAGELPFAWK---------EFNIKLVDEEDG-------------- :  235
CsAGO2/3a : ITDKLFSDDPTRFPELISTYDGEKNIFSAVPLPTG-----------TFYVELSGGEDTAP------------ :  308
CsAGO2/3b : ITDKLFSDDPTRFPELISTYDGEKNIFSAVPLPTG-----------TFYVELSGGEDTAP------------ :  326
CsAGO6    : IDKLYQTY-SSELAGKKFAYDGEKTLYTVGPLPQNIS---------EFTVVLEESFAK--------RGSPPH :  143
CsAGO4c   : IERVHETY-DTELAGKDFAYDGEKSLFTIGPLPRNKL---------EFTVVLEDVSS----NRNNGNRSPG- :  150
CsAGO4a   : IDKVHETY-ESELGKKDFAYDGEKSLFTVGALPRNKM---------EFTVVLDSVSPKSFGSKTNGSGSPGG :  163
CsAGO4b   : IDRVHETY-DTELAGKDFAYDGEKSLFTVGPLPRNKL---------EFTVVLEDISS----NRNNGNRSPG- :  218
CsAGO2/3c : IREKLFSDDPTRFPEQMSAYDGEKNIFSAMPLPEG-----------NSVVVLSGGEDVAS------------ :  242
CsAGO10b  : MAELVKLYKESDLGKRLPAYDGRKSLYTAGELPFAWK---------EFNIKLVDEEDG-------------- :  225
CsAGO10c  : MAQLVKLHRDTDLGRMLPVYDGRGALYTAGLMPFTLK---------KFTIILADGDES-------------- :  178
CsAGO1    : MEQLVKMYRESHLGKRLPAYDGRKSLYTAGPLPFTSK---------EFKITLIDEDDG-------------- :  394
CsAGO5c   : VNALIASYQESHLGKRCPAYDGRKSLYTAGPLPFVSQ---------DFVVKLVERET--------------- :  308
CsAGO5a   : INKLVELYRESHLGKRQLAYDGRKSVYTAGPLPFSSK---------DFVIKLVDQDR--------------- :  197
CsAGO5b   : ISELIASYRESHLGKLWPAYDGRKSLFTAGKLPFISK---------DFVVKLVDKYD--------------- :  213
CsAGO2/3e : IKDKLFSDDPSQFPLSSTAYDGDKNIFSAVELPTE-----------KFKVKFSDGEGMKC------------ :  385
            6                 aYDG   653    p                  l
```

```
                  440        *        460        *        480        *        500
CsAGO2/3f : ------------------VSYIVTIKEVKKLRFGKLKDYLSG---EIMSIPRDILQGMDLVTKENPSRE-RI :  361
CsAGO2/3d : ------------------SSYTVTIKHVNELELGKLKEYLSG---KVLSIPRDVLQGMDLVMKENPSRH-RI :  220
CsAGO7    : ------------EMHPQLKLFRINIKLVSKFDGRGLSSYLSKEEDDWVPLPQDYLHALDVVLRESPLEK-CI :  323
CsAGO10a  : -----------TNGPKRVREYKVVIKFAARANLHHLGQFLAG---KRADAPQEALQILDIVLRELATKR-YC :  292
CsAGO2/3a : ------------------RSYRFAIELVNELKLSKLNAYLRG---MLSYVPRDVFQAMDLVMKDNPSRH-MI :  358
CsAGO2/3b : ------------------RSYRFTIELVNELKVSKLNDYLRG---MLSYVPREIFQGMDLVMKDNPSRH-MN :  376
CsAGO6    : NGSPSESS-KRSKRSFQSKSFNVEINYAAKIPLKSISLALQG---SEVENIQDALRVLDIILRQQAANRGCL :  211
CsAGO4c   : --SPNGNDRKRQRRPYQSKTFKVEISFAAKIPMQAIANALRG---HESENSQEALRVLDIILRQHAAKQGCL :  217
CsAGO4a   : NGSPNESDRKRLRRAPQSKTFKVEISFAAKIPVQAIASALRG---QESENSQEALRVLDIILRQHASKQGCL :  232
CsAGO4b   : --SPNENDRKRQRRPYQSKTFKVEISFAAKIPMQAIANALRG---QESENSQEGLRVLDIILRQHAAKQGCL :  285
CsAGO2/3c : ------------------RSYTFTIQLVNKLKLSKLNDYLRG---MLSYIPRDILQGMDLVMKENPCQH-RI :  292
CsAGO10b  : -----------INGPKRERVYKVVIKFVARANLHHLGQFLAG---KRADAPQEALQILDIVLRELSSKR-YC :  282
CsAGO10c  : -----------IGMAK-EREFEVTIKFGAHASMHQLRELLSG---KQVDTPLEALKIIDIVLRELAAQR-YV :  234
CsAGO1    : -----------TGGARREREFRVVIKLAARADLHHLGLFLQG---RQADAPQEALQVLDIVLRELPTMR-YC :  451
CsAGO5c   : -------------TSRREREFRVAIKFAAKADLHHLQQFLHG---RQLDVPQETIQVLDVVLRSTPSNN-YA :  363
CsAGO5a   : -------------GARKDREFKVSIKFAAKPDIHHLQEFMRG---RQLDTPQETIQVLDVVLRATLSMN-NA :  252
CsAGO5b   : -------------QTRREREFKVAIKFAAKADIHHLQEFLRG---RQFDAPQETVQVLDVVFRSTPSTI-YT :  268
CsAGO2/3e : ------------------VSYIVTIKEVKKLRFGKLKDYLSG---EIMSIPRDILQGMDLVTKENPSRE-RI :  435
                                5   I          6   6 g        p      6D66 4
```

```
                 *       520         *       540         *       560         *
CsAGO2/3f : PVGRSFYPTTSNKDDDLDGGVTASKGFQHSLKPTSQGLALCLDYSVLAFHKRLPVIDFLKEHVWGFKQVNDM :  433
CsAGO2/3d : CAGRGFYSATS--ADDLGVGIIASKGFQQSLKPTSQGLALCLDYSVLAFRKRLPVIDFLKEHVRGFRGVNDV :  290
CsAGO7    : PVGRSLYSSSMGGAKDIGGGAIGLRGFFQSLRPTQQGLALNVDFSVTAFHESIGVIAYLQKRLEFLCDLSQR :  395
CsAGO10a  : PVGRSFFSPDIRKPQRLGDGLESWCGFYQSIRPTQMGLSLNIDMASAAFIEALPVIEFVAQL-----LGKDA :  359
CsAGO2/3a : SVGRSFYSKEFRPNEDLGYGIAAFRGSQQSLKATAQGLALCLDSSVLAFRKRLPVLDFLME--QGFQ-LNDV :  427
CsAGO2/3b : SVGRSFFSKEFRPNDDLGYGVAAFRGFQQSLKATAQGLALCLDSSVLALRKRLPVLDFLME--QGFQ-LNDV :  445
CsAGO6    : LVRQSFFHDDSRNFTNVGGGVTGCRGFHSSFRPTHGGLSLNMDVSTTMILTPGPVIDFLLAN-----QNARE :  278
CsAGO4c   : LVRQSFFHNDPRNFADVGGGVLGCRGFHSSFRTTQGGLSLNIDVSTTMIIQPGPVVDFLIAN-----QNVKD :  284
CsAGO4a   : LVRQSFFHDNQSNFTDLGGGVLGCR-----------------DGSTTTIVQPGPVIDFLIAN-----QGVRD :  282
CsAGO4b   : LVRQSFFHNDPRNFSDVGGGVLGCRGFHSSFRTTQGGLSLNIDVSTTMIIQPGPVVDFLIAN-----QNVKD :  352
CsAGO2/3c : SVGRSFYSKEFRPRDDLGYGIAAYRGFQQSLKPTSQGLALCLDYSVLAFRKRLPVIDFLVENVEGFR-VNDM :  363
CsAGO10b  : PVGRSFFSPDIRKPQRLGDGLESWCGFYQSIRPTQMGLSLNIDMASAAFIEALPIIEFVAQL-----LGKDV :  349
CsAGO10c  : SVGRFFYSPNINRPQPLGNGLQSWRGFYQSIKPTQMGLSLNIDMSATAFIEPLPVIEFVAQI-----LGKDV :  301
CsAGO1    : PVGRSFYSPDLGRRHPLGEGLESWRGFYQSIRPTQMGLSLNIDMSSTAFIEPLPVIDFVTQL-----LTRDV :  518
CsAGO5c   : VVGRSFFSPQLGDTGHLGDGIEYWKGYYQSLRPTQMGLSLNIDISARAFYEPVLVSDFVATY-----CNIRD :  430
CsAGO5a   : VVGRSFFSPSFG-VGDLGNGFQYWKGFYQSLRPIQMGLSLNIDMSARAFYEPILVTDFVAKY-----FNVKD :  318
CsAGO5b   : VVGRSFFSPILGEVGDLGDGLQYWKGFYQSLRPTQMGLSLNIDMSARAFYEPILVSDFVAKY-----FNVRD :  335
CsAGO2/3e : PVGRSFYPTTSNKDDDLDGGVTASKGFQHSLKPTSQGLALCLDYSVLAFHKRLPVIDFLKEHVWGFKQVNDM :  507
             vgrsf5         6g G     g   s   t  gl l  D s  a     p6 d56
```

```
             580         *       600         *       620         *       640
CsAGO2/3f : RR--LRRDVIN----ALTGLKVRVTHRVTNQKYIIVGLTDQNTR--NISFDVEDREEKAPPKRVSLVEYFRE :  497
CsAGO2/3d : TR--LRTDVKN----ALEGLKVTVNHRITKQKYTIAGLTDRSTR--DLSFDNVDPEGKAPPRRVSLVEYFRG :  354
CsAGO7    : KTRGLAGEERKEVEKALKNIRVFVCHRETVQRYRVYSLTEEATE--NLWFPDRDG------RTLRLVNYFKD :  459
CsAGO10a  : ISRTLSDSDRVKIKKALRGVKVEVTHRGHVRRKYRVQGLTSQP------TRELVFPVDDDSTMKSVVEYFQE :  425
CsAGO2/3a : RT--SRREVMQKVMHVLKGLKVHVTHRLTSQKYTIAGFSDQNTR--DITFTLEDREGENPPRETGLVEYFRE :  495
CsAGO2/3b : RT--SRREVMREVMHVLKGLKVHVTHRLTNQKYTIAGFSDQNTR--DITFTLEDREGENPPREIGLVEYFRE :  513
CsAGO6    : PR----NIDWAKAKKMLKNMRVKTRHSNMEFKIIGLSEKPCNQQFFSLKVRNADGGYGGEALEITVYDYFTK :  346
CsAGO4c   : PF----SIDWAK-------------------------------------QKGKDDDGEVQTLEVTVYDYFVN :  315
CsAGO4a   : PF----QVDWAKAKRTLKNLRIKVTPSNMEYRIIGLSESPCKEQMFSLKTRGQKD--ENESVDVTVYDYFVN :  348
CsAGO4b   : PF----SIDWAKAKRILKNLRVKTNPSNTEYKITGLSEKPCKEQMFSLKQKGKDDDGEVQTLEVTVYDYFVN :  420
CsAGO2/3c : KR--SRRDVMR----ALKGLKVHVTHRVTKQKYIVAGLTNQNAR--DITFTLEDPEGENPPREIGLIEYFRE :  427
CsAGO10b  : ISRTLSDSDRVKIKKALRGVKVEVTHRGNVRRKYRVSGLTSQP------TRELVFPVDDNSTMKSVVEYFQE :  415
CsAGO10c  : YSRPLSDADRVKVKKALRGIKVEVTHRGNMRRKYRIAGLTSQP------TRELIFPVDEEKNMKSVVEYFQE :  367
CsAGO1    : SSRPLSDADRVKIKKALRGVKVEVTHRGNMRRKYRITGLTSQA------TRELTFPVDERGTMKSVVEYFHE :  584
CsAGO5c   : LSRPLSDQDRIKVKKALKMVKVELIHSEDVKR--KISGVSVQP------TSQLTFTSGDTGTQTSVVKYFRE :  494
CsAGO5a   : LTRPLSDQDCLKVKRALRGIKVELAHRKHVQCR-RISGLSVRP------TNQLTFPLDDTGEKMSVVEYFRQ :  383
CsAGO5b   : LTRPLSDQDRIKVKRALKGIRVELTHREDGKRY-KISGVSAQP------TSQLTFARDDTDAKISVIQYFRE :  400
CsAGO2/3e : RR--LRRDVIN----ALTGLKVRVTHRVTNQKYIIVGLTDQNTR--NISFDVEDREEKAPPKRVSLVEYFRE :  571
                            l        h                                       6  YF
```

```
             *       660         *       680         *       700         *       720
CsAGO2/3f : KYNKEIKH-KDIPCLDLGKNNRINYVPMEFCILVEGQRFPREHLSRDGTILLKNMSLVPPKDRMNTICEMVQ :  568
CsAGO2/3d : KYGKEIMY-KDIPSLDLGKNGRINYVPMEFCILVEGQRFPKENLNDRGAKLLKEMSLIRPAVRRKTICEMVQ :  425
CsAGO7    : HYNYDIQF-RNLPCLQISR-SKPCYLPMELCMICEGQKFLG-KLSDDQTAKILKMGCQRPRQRKAIIDEVMA :  528
CsAGO10a  : MYGFTIQY-THLPCLKVGNQKKANYLPMEACKIVEGQRYTK-RLNEKQILLVQMVTCQRPRDRENDILQTVQ :  495
CsAGO2/3a : KYNRELRY-QDMPCVDLGKGNRKNYVPMEFCVLVEGQRYPKEHLDRDQALFLKQISLPRPRERKDIIYGMVR :  566
CsAGO2/3b : KYNRELRY-QDMPCVDLGKGNRKNYVPMEFCVLVDGQRYPKEHLDRDQALFLKQISLPRPRERKDTIYGMVR :  584
CsAGO6    : HRNIELTYSAFMPCLDVGKPKRPNYLPLELCTLVSLQRYTK-ALSSMQRASLVEKSRQKPQERIR---VALK :  414
CsAGO4c   : YRNIELRYSADLPCINVGKPKRPTYFPLELCSLVSLQRYTK-ALSTFQRASLVEKSRQKPQERMKVLTDALK :  386
CsAGO4a   : YRHLELRYSADLPCVNVGKPKRPTYIPIELCMLVSLQRYTK-ALTVHQRSSLVEKSRQKPQEKIRILTDVMK :  419
CsAGO4b   : YRHIDLRYSGDLPCINVGKPKRPTYFPLE----------------------------------------ALK :  452
CsAGO2/3c : KHNKEIKY-QDMPCLDLGKNNRKNYVPLEFCVLLEGQRYPKEQLDKETALFLKKISLPQPRERKNTIYEMVR :  498
CsAGO10b  : MYGFTIQH-THLPCLQVGNQKKANYLPMEACKIVGGQRYTK-RLNEKQITALLKVTCQRPRDRENDILQTVQ :  485
CsAGO10c  : VYGFTIQH-PHLPCLQVGNPRKVNYLPMEACKIVDGQRYTK-RLNDKQITSLLKFSCQRPQEQEMDILKTMH :  437
CsAGO1    : TYGFVIQH-TQWPCLQVGNQQRPNYLPMEVCKIVEGQRYSK-RLNERQITALLKVTCQRPQEREQDILQTVH :  654
CsAGO5c   : KYDIALKF-TALPALQVGSDSRPIYLPMELCKIVPGQRYSK-RLNDRQVSALLRATCQRPNDRERSINQMVK :  564
CsAGO5a   : KYNIILEY-AFLPALVAGSDAKPVYLPMEICKIAEGQRYSK-KLNEKQVSALLKATCQRPDVREGSIKQIVR :  453
CsAGO5b   : RYHINLKF-AFLPALQAGSDLKPVYLPMELCKIVEGQRYSK-KLNEKQVTALLRATCQRPFERVQSISRMVE :  470
CsAGO2/3e : KYNKEIKH-KDIPCLDLGKNNRINYVPMEFCILVEGQRFPREHLSRDGTILLKNMSLVPPKDRMNTICEMVQ :  642
                 6      Pc6  g   4  Y P6E c    gq      l               p          6
```

```
                   *       740          *       760          *       780          *
CsAGO2/3f : ADDGPCGGNIAQNFGIQVNKNMTKVEGRVIGPPQLKFGASNGKVNVVRVD-KEKCQWNLVGKSVVDGKPVKR : 639
CsAGO2/3d : AEDGPCGGNVARGFDIEVIKDMTMVKGRVIGPPQLKLRAPDGNLNAVSVD-RVKCHWNLVRRSVVEGKPVQS : 496
CsAGO7    : GPVGPTSGIQGREFKLHVSREMTRLNGRILQPPKLKLGD--GGFIRDLIPSRYDRQWNLLDSHVFEGTRIDR : 598
CsAGO10a  : QNAYDQDPYAK-EFGIKISEKLTSLEARVLPAPWLKYHETGKEKDCLPQV----GQWNMMNKKMINGMTVNR : 562
CsAGO2/3a : SEDGPCG-VVARNFGIEVKKDMTRLLGRGMEPPVLKVGSSTGNVMVLRINDKDKCQWNLLGKSVMEGMPLNR : 637
CsAGO2/3b : SEDGPCG-DVARNFGIEVKKDMTRLLGRGMEPPVLKVGSSTGSAVVLRITDKDKCQWNLLGKSVMEGMPLDR : 655
CsAGO6    : TYRYDDDPLFV-ACGISIEKQLTQVDGRVLEAPKLKVGNG---EDCIPRN----GRWNFNNKQLFTPIRIER : 478
CsAGO4c   : INNYDAEPMLR-SCGISISSNFTQVEGRVLPAP----------------------------RKLVDPTKIER : 429
CsAGO4a   : SNNYGSDPMLR-SCGVSIQANFTQVEGRVLSAPRLKLGNG---EDLMPRN----GRWNLNNKKFVEPTKIKN : 483
CsAGO4b   : LNKYEAEPMLK-SCGISIGTNFTQVEGRVLPAPRLKVGNG---EDFFPRN----GRWNFNNKKLVDPTKIDR : 516
CsAGO2/3c : SQDGPCG-DVSQNFEIEVEKNMTGVLGRVIEPPDLKLGTPNGNVNVLRINDRDKCQWNLLGKAVVGGKPLER : 569
CsAGO10b  : HNAYDQDPYAK-EFGIKISEKLASVEARVLPAPWLKYHETGKEKDCLPQV----GQWNMMNKKMINGMTVSR : 552
CsAGO10c  : QNGYNQDPYAK-EFGISIDDKLTSVEARVLPAPWLKYNDNGKEKEFLPKV----GQWNMMNKKVINGGTVNY : 504
CsAGO1    : HNAYADDPYAK-EFGIKISEKLAAVEARVLPAPWLKYHDTGREKDCLPQV----GQWNMMNKKMVNGGTVNN : 721
CsAGO5c   : HNNYNNDKFVN-EFGLRVRTELTSVDARVLPAPMLKYQQDT----QETPRV----GAWNMIDKKMVNGGKVEF : 627
CsAGO5a   : HNNYNNDKLVNEEFGIQVRAELASVDARVLPPPRLKYHDTGRETQVDPRV----GVWNMIDKKMVNGGKVEF : 521
CsAGO5b   : RNNYNNDKFVN-EFGLRVKTELTSIDARVLPAPMLKYQGT----QEVPRV----GCWNMIDKKMVNGGKVEF : 533
CsAGO2/3e : ADDGPCGGNIAQNFGIQVNKNMTKVEGRVIGPPQLKFGASNGKVNVVRVD-KEKCQWNLVGKSVVDGKPVKR : 713
                       fg6 6     t 6  R 6  P 1k                    wn        g  6
```

```
                  800          *       820          *       840          *       860
CsAGO2/3f : WALLDFSSSDRF------------------------------------------------------------ : 651
CsAGO2/3d : WALLDFSSSD-------------------------------------------------------------- : 506
CsAGO7    : WALISFGGSADQ------------------------------------------------------------ : 610
CsAGO10a  : WACINFSRSVQ------------------------------------------------------------- : 573
CsAGO2/3a : WALIDFTANDRYN----------------------------------------------------------- : 650
CsAGO2/3b : WALIDFTANDRYN----------------------------------------------------------- : 668
CsAGO6    : WAVVNFSARCDTS----------------------------------------------------------- : 491
CsAGO4c   : WAVVNFSARCDVR----------------------------------------------------------- : 442
CsAGO4a   : WVVVNFSARCDVR----------------------------------------------------------- : 496
CsAGO4b   : WAVVNFSARCDIR----------------------------------------------------------- : 529
CsAGO2/3c : WALIDFSSYDRY------------------------------------------------------------ : 581
CsAGO10b  : WACINFSRSVQ------------------------------------------------------------- : 563
CsAGO10c  : WACINFSRSVQ------------------------------------------------------------- : 515
CsAGO1    : WICINFSRNVQ------------------------------------------------------------- : 732
CsAGO5c   : WTCVSFSRIRQ------------------------------------------------------------- : 638
CsAGO5a   : WTCISFSRSRINLDQFCCELISMCRSKGMDFNEPFLPIKSAYPGQIEKDLHDIHACSTEKLANMGLTGKQLQ : 593
CsAGO5b   : WTCVSFSRLRQ------------------------------------------------------------- : 544
CsAGO2/3e : WALLDFSSSDRF------------------------------------------------------------ : 725
            W  6 F
```

```
                  *        880          *        900          *        920          *
CsAGO2/3f : ------------------------------------------------------------------------ :   -
CsAGO2/3d : ------------------------------------------------------------------------ :   -
CsAGO7    : ------------------------------------------------------------------------ :   -
CsAGO10a  : ------------------------------------------------------ESVAR------------- : 578
CsAGO2/3a : ------------------------------------------------------------------------ :   -
CsAGO2/3b : ------------------------------------------------------------------------ :   -
CsAGO6    : ------------------------------------------------------------------------ :   -
CsAGO4c   : ------------------------------------------------------------------------ :   -
CsAGO4a   : ------------------------------------------------------------------------ :   -
CsAGO4b   : ------------------------------------------------------------------------ :   -
CsAGO2/3c : ------------------------------------------------------------------------ :   -
CsAGO10b  : ------------------------------------------------------ESVAR------------- : 568
CsAGO10c  : ------------------------------------------------------ESAVR------------- : 520
CsAGO1    : ------------------------------------------------------DSVSR------------- : 737
CsAGO5c   : ------------------------------------------------------DEVF-------------- : 642
CsAGO5a   : LLIIVLPDEKRYSKKLNEKQVSALLKATCQRPDVREGSIKQMVRHNNYNNDKLVNEEFGIQVRAELASVDAR : 665
CsAGO5b   : ------------------------------------------------------NEVF-------------- : 548
CsAGO2/3e : ------------------------------------------------------------------------ :   -
```

```
             940          *        960          *        980          *       1000
CsAGO2/3f : -------------------------------------------------KLDHGKFVLNLTNRCQKLGIHIE : 674
CsAGO2/3d : -------------------------------------------------LNY---FVPQLIKKCNNLGIRMN : 526
CsAGO7    : -------------------------------------------------KSAIPKFINQLSQRCEQLGIFLN : 633
CsAGO10a  : ------------------------------------------------------GFCSELAQMCQVSGMEFN : 596
CsAGO2/3a : -------------------------------------------------KLNPDRFIPNLRARCYNLGIQVE : 673
CsAGO2/3b : -------------------------------------------------KLNPDRFIPNLRARCYNLGIQVE : 691
CsAGO6    : ------------------------------------------------------HLSRELINCGRNKGINIE : 509
CsAGO4c   : ------------------------------------------------------GLVRDMIKCGDMKGIHVE : 460
CsAGO4a   : ------------------------------------------------------GLCRDLARVGEMKGISID : 514
CsAGO4b   : ------------------------------------------------------GLVRDLIKCGDMKGIQID : 547
CsAGO2/3c : -------------------------------------------------RLNPSHFVQNLRNRCRNLGIRVE : 604
CsAGO10b  : ------------------------------------------------------GFCNELAQMCQVSGMEFN : 586
CsAGO10c  : ------------------------------------------------------GFCHQLAQMCQGSGMEFN : 538
CsAGO1    : ------------------------------------------------------GFCNELAQMCVICGMVFN : 755
CsAGO5c   : ------------------------------------------------------RFCENLVYMCQSKGMDFN : 660
CsAGO5a   : VLPPPRLKYHDTGKETQVDPRVGVWNMIDKKMVNGGKVEFWTCISFSRSRINLDQFCRELISMCRSKGMDFN : 737
CsAGO5b   : ------------------------------------------------------KFCENLVDMCRNKGMDFN : 566
CsAGO2/3e : -------------------------------------------------KLDHGKFVLNLTNRCQKLGIHIE : 748
                                                                  f   6   c   G6
```

```
                 *       1020          *       1040          *       1060          *       1080
CsAGO2/3f : HPLVYRPTDMRQLSDITRLRSLLESVISEANGRCQG--RLQIIVCVMTRIDPG--YKYLKWLSETQIGIITQ : 742
CsAGO2/3d : EPLVYRSTNMRPFSDIDQLRSLLIENVIKEANEKYKS--QLQIIVCVMDGKHPG--YKSLKWLTETQIGILTQ : 594
CsAGO7    : KNTVVGP--QFESIQVLNNVSLLESKLKKIYRAASN--NQLQLICVMERRHKG--YADFKRIAETSVGVLSQ : 699
CsAGO10a  : PEPVIPIYTSRPDQVEKALKHVYHSCTNKLK--GKE---LELLLAILPD-NNGSLYGDIKRICETDLGLMSQ : 662
CsAGO2/3a : DPVVCRFTYMNKFSSLATIQELLDSVVKEASRNCKG--KLQLIFCVMSGKHDG--YKYLKWVSETQIGVLTQ : 741
CsAGO2/3b : DPVACRFTNMNKFSSLATIQELLDSVVKEASRNCKG--KLQLIFCVMSGKHDG--YKYLKWVSETQIGVLTQ : 759
CsAGO6    : RPHTLVEEDP---QYRRSSPIVRVEKMFELITGKLP-GPPQFLLCVLPEKKNSDIYGPWKKKSLSDFGIATQ : 577
CsAGO4c   : PPFDVFEENA---QNRRAPPLVRVEKMFEQIQSKLP-GAPQFLLCLLPERKNSDIYGPWKKKNLADFGIVTQ : 528
CsAGO4a   : PPADVFEENP---QFRRAPPPVRVEKMFEQMMPKLPHGPPHFILCLLPDRKNSDVYGPWKKKNLADFGIFNQ : 583
CsAGO4b   : APFDVFEESP---QNRRAPPLVRVEKMFEDIQSKLP-GAPQFLLCLLPERKNSDLYGPWKRKNLADFGIVTQ : 615
CsAGO2/3c : EPVVRRFTNMNEFSSIAAIQKLLHNVVEEASQKCKG--QLQLIVCVMSARHDG--YKYLKWVSETQIGVLTQ : 672
CsAGO10b  : PEPVIPIYTAKPEQVEKALKHVYHVCMNKLK--GKE---LELLLAILPD-NNGSLYGDIKRICETDLGLISQ : 652
CsAGO10c  : FEPVIPIYSARPDQVKKALKYVCSAAANNHG--GKE---LELLIAILPD-NNGSLYGDLKRICETELGIISQ : 604
CsAGO1    : RDPVLAPLSARPDQVERVLKARFHDAMTKLQPQGKE---LDLLIVILPD-NNGSLYGDLKRICETDLGIVSQ : 823
CsAGO5c   : SMPLLEPRSAHPAQIEKTLLEIHKQSNAKLASSGK---LQLLIIILPD-ATG-TYGRIKRVCETELGIISQ : 727
CsAGO5a   : -EPFLPIKSAYPGQIEKDLHDIHAHSTEKLANMGLTGKQLQLLIIVLPD-VSG-SYGKIKRVCETELGIVSQ : 806
CsAGO5b   : LMPLIPIRLAHPAQIERTLVEIHTQSNVQLTSIGKADEQLQMLIIILPE-VTG-SYGRIKRVCETELGIISQ : 636
CsAGO2/3e : HPLVYRPTDMRQLSDITRLRSLLESVISEANGRCQG--RLQIIVCVMTRIDPG--YKYLKWLSETQIGIITQ : 816
                                                       l  6  66      g   Y    K    e    G6   Q
```

```
                     *      1100         *      1120         *      1140         *
CsAGO2/3f : CCLFNHANNAN-DRYFANLALKINAKLGGSNVELIEPLP-----RFEGEGHVMFVGADVNHPGARNSTCPSI :  808
CsAGO2/3d : CCLSNHAYEAK-DQYLANLALKINAKLGGSNVELNQRLP-----RFDGEGHVMFVGADVNHPAARNSTCPSM :  660
CsAGO7    : CCLYPNLGKLS-SQFLANLAIKINAKIGGCTVALYNSLPSQIPRLFQADDPVIFMGADVTHPHPLDDFSPSV :  770
CsAGO10a  : CCLTKHVFKIS-KQYLANVSLKINVKMGGRNTVLLDAISCKIP--LVSDIPTIIFGADVTHPENGEDSSPSI :  731
CsAGO2/3a : CCLVGIANKGK-DQDLANIALKLNCKLGGSNVELNGPLP-----GFEGDEHVMFVGADVNHPKAFNASCPSI :  807
CsAGO2/3b : CCIVGIANKGK-DQDLANIALKLNCKLGGSNVELNGMLP-----GFEGDEHVMFVGADVNHPKAFNASCPSI :  825
CsAGO6    : CISP---TKIN-DQYLTNVLLKINSKLGGTNSLLAIEHASRVP--LIKDTPTMILGMDVSHGSPGRSDIPSI :  643
CsAGO4c   : CIAP---TRVN-DQYLTNVLLKINAKLGGLNSMLAVEHAPSIP--LVSKVPTLILGMDVSHGSPGQSDIPSI :  594
CsAGO4a   : CLAP---MRVN-DQYLNNLLLKINAKLGGLNSILSIEPARNIP--VVSNNPTMIFGMDVSHGSPGHSDVPSI :  649
CsAGO4b   : CIAP---TRVN-DQYLTNVLLKINAKLGGLNSMLSVEHSPSIP--LISKVPTIILGMDVSHGSPGQSDIPSI :  681
CsAGO2/3c : CCLVGNANKGN-DQYLANLALKINGKIGEVLNELQSPP-----------------------------SVPSS :  714
CsAGO10b  : CCLTKHVFKIT-KQYLANVSLKINVKMGGRNTVLLDAISCRIP--LVSDIPTIIFGADVTHPENGEDSSPSI :  721
CsAGO10c  : CCLTKHVLKIS-RQYLANLSLKINVKMGGRNNVLLDALSWRIP--LVSDIPTIIFGADVTHPESGEDCSPSI :  673
CsAGO1    : CCLTKHVFRMS-KQYLANVALKINVKVGGRNTVLVDAISRRIP--LVSDLPTIIFGADVTHPHPGEDSSPSI :  892
CsAGO5c   : CCQPKQASKCQ-KQYLENVALKINVKAGGRNNVLLNALHKKIP--YVTDAPTIIFGADVTHPQPGEDASPSI :  796
CsAGO5a   : CCQPRQAQKNNNKQYLENLSLKINVKVGGRNTVLMSAIERRIP--FVTDRPTIIFGADVTHSQPGDDASPPI :  876
CsAGO5b   : CCQPKQAMKCN-KQYLENVSLKINVKAGGRNTVLANALEKKIP--FLTDLPTIIFGADVTHPQPGEDSSPSI :  705
CsAGO2/3e : CCLFNHANNAN-DRYFANLALKINAKLGGSNVELIEPLP-----RFEGEGHVMFVGADVNHPGARNSTCPSI :  882
            Cc           q l N6 6K6N K Gg n  L                     g dv h        Ps

                1160         *      1180         *      1200         *      1220
CsAGO2/3f : AAVVATVNWPAANRYAARVCPQYHRSEKILNFGTMCLELIQAYARIN------------------------- :  855
CsAGO2/3d : AAVVATLNWPAANRYAARVCPQYHRKENILNFGVMCQELIRTYAQIN------------------------- :  707
CsAGO7    : AAVVGSVNWPAANKYVSRMRSQTHRQEIIEDLTAMVGEILDDFYQEQ------------------------- :  817
CsAGO10a  : AAVVASQDWPEVTKYAGLVCAQAHRQELIQDLYKTWHDPVRGPISGG-------MIRDLLVSFRKATG---- :  792
CsAGO2/3a : AAVVASVNWPAANRYVARICPQEHRKEKIVNFGAICLDLVNTYAKLN------------------------- :  854
CsAGO2/3b : AAVVASVNWPAANRYVARICPQEHRKEKIVNFGPMCLDLVNTYAKLN------------------------- :  872
CsAGO6    : AAVVGSRSWPLISRYRAAVRTQSSKVEMVESLFKPLANGKDDGIM-----------RELLVDFYQTS----- :  699
CsAGO4c   : AAVVSSRHWPLISRYRASVRTQSPKVEMIDSLYKRVSDTEDEGIFSKT---LSFNFRELLLDFYVS------ :  657
CsAGO4a   : AAVVSSRHWPLISRYRASVRSQSSKVEMIDSLFKRGADNEDTGIGVVAGLLFKFRAKKAITNYNFQGLAAWS :  721
CsAGO4b   : AAVVSSRQWPLISRYRASVRTQSPKVEMIDSLYKRVSESEDEGIM-----------RELLLEFYVT------ :  736
CsAGO2/3c : SALTPAVSLNESTYITCKESKQCKDKAAIQ----------SDKITLK------------------------- :  751
CsAGO10b  : AAVVASQDWPEVTKYAGLVCAQAHRQELIQDLYKTWHDPVRGTVSGG-------MIRDLLVAFRKATG---- :  782
CsAGO10c  : AAVVASQDWPEVTKYAGLVCTQPHRQELIQDLYKTWQDPQGGTVGGG-------MIRELLLSFQKATG---- :  734
CsAGO1    : AAVVASQDWPEVTKYAGLVCAQEHRQELIQDLFKSWQDPVRGTVTGG-------MIKELLISFRRATG---- :  953
CsAGO5c   : AAVVASMDWPEVTKYRGLVSAQPHRDEIINDLYKTIQDPQRGTVHSG-------MIRELLIAFRRSTG---- :  857
CsAGO5a   : AAVVASMDWPEVTKYRGLVSAQGHKEEIIQDLYKTYRDPQRGTVHCG-------MIRELLIAFKTSTG---- :  937
CsAGO5b   : AAVVASMDWPEATKYRGLVSAQPHREEIIQDLYKTSLNAKNEIVHGG-------MIRDMLISFRRSTG---- :  766
CsAGO2/3e : AAVVATVNWPAANRYAARVCPQYHRSEKILNFGTMCLELIQAYARIN------------------------- :  929
            aA6v    wp    y      Q    e 6

                 *      1240         *      1260         *      1280         *
CsAGO2/3f : ----------------------------------------KVKPQRIVVF-RDGVSEGQFDMVLNEELFDLK :  886
CsAGO2/3d : ----------------------------------------KVKPARIVVF-RDGVSEGQFDMVLNEELLDLK :  738
CsAGO7    : ----------------------------------------TKLPKRIIFF-RDGVSETQFYKVLREELQAIQ :  848
CsAGO10a  : -----------------------------------------QKPLRIIFY-RDGVSEGQFYQVLLYELDAIR :  822
CsAGO2/3a : ----------------------------------------KVKPKKIVVF-RDGVSEGQFDMVLNEELLDLK :  885
CsAGO2/3b : ----------------------------------------KVKPKKIVVF-RDGVSEGQFDMVLNEELLDLK :  903
CsAGO6    : --------------NG-------------------------RKPAQIILF-RDGVSESQFNQVLNTELDQMI :  731
CsAGO4c   : --------------SG------------------------KRKPDQIILF-RDGVSESQFNQVLNIELDQII :  690
CsAGO4a   : VVVRDGGFIMALHPCGGQAALACSIERCCAIEPVFRILRIETNPNKLIHYGMDGVSESQFNQVLNIELDQII :  793
CsAGO4b   : --------------SG------------------------KRKPDQIIIF-RDGVSESQFNQVLNIELDQII :  769
CsAGO2/3c : ----------------------------------------KVP----------------------------- :  754
CsAGO10b  : -----------------------------------------QKPLRIIFY-RDGVSEGQFYQVLLYELDAIR :  812
CsAGO10c  : -----------------------------------------QKPLRIIFY-RDGVSDGQFYQVLLYELDAIR :  764
CsAGO1    : -----------------------------------------QKPQRIIFY-RDGVSEGQFYQVLLYELDAIR :  983
CsAGO5c   : -----------------------------------------HKPHRIIFY-RDGVSEGQFNQVLLYEMDAIR :  887
CsAGO5a   : -----------------------------------------QKPHRIIFY-RDGVSEGQFNQVLLHEMDAIR :  967
CsAGO5b   : -----------------------------------------QKPCRIIFY-RDGVSEGQFNQVLLYEMDAIR :  796
CsAGO2/3e : ----------------------------------------KVKPQRIVVF-RDGVSEGQFDMVLNEELFDLK :  960
                                                      kp       rdgvse qf  vl  e

            1300         *      1320         *      1340         *      1360
CsAGO2/3f : KAIYEE--HYRPTITLVVAQKRHQTRLFP--------EKMKDGGSTGNVPPGTVVDKKITHPFEFDFYLCSH :  948
CsAGO2/3d : MAIYEE--HYRPTITLVVAQKRHQTRLFP--------ESMKDGSPDGNVVPGTVVDTVITHPYDFDFYLCSH :  800
CsAGO7    : AACSRFP-SYKPPITFAVVQKRHHTRLFPNETQTHQQSSTRDQFLDENIPPGTVVDTVITHPREFDFYLCSH :  919
CsAGO10a  : KACASLEPNYQPPVTFIVVQKRHHTRLFANN-----HRDRSSTDKSGNIVPGTVVDSKICHPTEFDFYLCSH :  889
CsAGO2/3a : NAIYEG--NYRPTITLVVAQKRHQTRLFL--------ENERDGGASGNVPPGTVVDTTIVHPFEFDFYLCSH :  947
CsAGO2/3b : NAIYEG--NYRPTITLVVAQKRHQTRLFL--------ENERDGGASGNVPPGTVVDTTIVHPFEFDFYLCSH :  965
CsAGO6    : KAYQHLGEATIPKFTVIVAQKNHHTKLFQSG-----APENVPPGLFLCSI-GTVVDTKVVHPRNYDFYMCAQ :  797
CsAGO4c   : ESCKFLDEKWSPKFVVIVAQKNHHTKFFQPG-----SPDNVPP--------GTVIDNKVCHPRNNDFYMCAH :  749
CsAGO4a   : EACKFLDENWSPKFTVIIAQKNHHTKFFQAG-----SPDNVPP--------GTVIDKEVCHPHNFDFYMCAH :  852
CsAGO4b   : EACKFLDEKWSPKFVVIVAQKNHHTKFFQPG-----SPDNVPP--------GTIIDNKICHPKNNDFYLCAQ :  828
CsAGO2/3c : --------------------------------------------APGKPSPG-------AQNF--------- :  766
CsAGO10b  : KACASLEPNYQPPVTFIVVQKRHHTRLFANN-----HRDRSSTDKSGNILPGTVVDSKICHPTEFDFYLCSH :  879
CsAGO10c  : KACASLESSYQPPVTFIVVQKRHHTRLFASN-----HNDKTSIDKSGNILPGTVVDTKICHPTEFDFYLCSH :  831
CsAGO1    : KACASLEPNYQPPVTFVVVQKRHHTRLFANN-----HRDRNAVDRSGNILPGTVVDSKICHPTEFDFYLCSH : 1050
CsAGO5c   : KACVSLEENYLPPVTFVVVQKRHHTRLFPAQ-----HNDRNSTDRSGNILPGTVVDTTICHPTEFDFYLCSH :  954
CsAGO5a   : KACVSLEEHYLPRVTFVVVQKRHHTRLFPAD-----HSDRRSTDRSGNILPGTVVDTHICHPNEFDFYLCSH : 1034
CsAGO5b   : KACVSLEADYLPRVTFVVVQKRHHTRLFPAE-----HGDRNSTDRSGNILPGTVVETKICHPTQFDFYLCSH :  863
CsAGO2/3e : KAIYEE--HYRPTITLVVAQKRHQTRLFP--------EKMKDGGSTGNVPPGTVVDKKITHPFEFDFYLCSH : 1022
             a         p  t    qk h t lf                      pGt  d    hp   dfy c h

             *      1380         *      1400         *      1420         *      1440
CsAGO2/3f : YGSLGTSKPTHYYVLWDEHNFTSDQLQKLIYDLCFT------------------FARCTKPVSLVPPVYYAD : 1002
CsAGO2/3d : YGSMGTSKPTHYYVLWDEHNFTSDQLQKLIYDMCFT------------------FARCTKPVSLVPPVYYAD :  854
CsAGO7    : WGVKGTSRPTHYHVLWDENQFTSDELQKLVYNLCYT------------------FVRCTKPVSLVPPAYYAH :  973
CsAGO10a  : AGIQGTSRPAHYHVLWDENNFTADGIQSLTNNLCYT------------------YARCTRSVSVVPPAYYAH :  943
CsAGO2/3a : YGNLGTSKPTHYYCLFDENKFTSDKLQKLIYHLCYT------------------FARCTKPVSLIPPVYYAD : 1001
CsAGO2/3b : YGNLGTSKPTHYYCLFDENKFTSDKLQKLIYHLCYT------------------FARCTKPVSLIPPVYYAD : 1019
CsAGO6    : AGMIGTSRPAHYHVLLDEIGFSPDDLQNLILSLSYV------------------YQRSTTAISIVAPVCYAH :  851
CsAGO4c   : AGMIGTTRPTHYHVLLDEVGFSADDLQELVHSLSYV------------------YQRSTTAISVVAPICYAH :  803
CsAGO4a   : AGMIGTTRPTHYHILFDEIGFSVDDLQELVHSLSYV------------------YQRSTTAISIVAPIRYAH :  906
CsAGO4b   : AGMIGTTRPTHYHVLLDEVGFSADDLQELVHSLSYV------------------YQRSTTAISVVAPICYAH :  882
CsAGO2/3c : ------------------------------------------------------------------------ :    -
CsAGO10b  : AGIQGTSRPAHYHVLWDENNFTADGIQSLTNNLCYT------------------YARCTRSVSVVPPAYYAH :  933
CsAGO10c  : AGIQGTSRPAHYHVLWDENNFSADEIQSLTNNLCYT------------------YARCTRSVSLVPPAYYAH :  885
CsAGO1    : AGIQGTSRPAHYHVLWDENKFTADGLQSLTNNLCYTYVSSFNPSQLLFLNHQLIYARCTRSVSIVPPAYYAH : 1122
CsAGO5c   : AGIQ---------------------------------------------------------VNSVPPAYYAH :  969
CsAGO5a   : AGIQGTSRPTHYHVLYDENQFSADALQT---------------------------------LTNMPPAYYAH : 1073
CsAGO5b   : AGIQGTSRPTHYHVLYDENKFTADALQMLTNNLCYT------------------YARCTRSVSVVPPAYYAH :  917
CsAGO2/3e : YGSLGTSKPTHYYVLWDEHNFTSDQLQKLIYDLCFT------------------FARCTKPVSLVPPVYYAD : 1076
             g  gt  p hy  l de  f  d  q l                           r t       p  ya
```

```
                         *      1460         *      1480         *      1500         *
CsAGO2/3f : LVAYRGRMFQEVMMEVSPVSVSSSSSSS---ATSTSSLSVS----SFDDKFYKLHRD--------------- : 1052
CsAGO2/3d : LVAFRGRMFQEVVMKLPPGSASSSSCSS---SSSHAMVSITQQSASFDEKFYKLHPE--------------- :  908
CsAGO7    : LAAYRGRLYLERSDSSSISPFSRNAAST---ISRAAP--------PKATPLPKLSEN--------------- : 1019
CsAGO10a  : LAAFRARFYLEPDMQENGSSGGGNA----------GKSTRANGESG-VRPLPALKEN--------------- :  989
CsAGO2/3a : LVAYRGRQYQEVVMEFQPPPSVSSSSSS---SSSS--------AASLNERFYALHPD--------------- : 1047
CsAGO2/3b : LVAYRGRQYQEVVMEFQPPPSVSSSSSS---SSS---------AASLNERFYALHLD--------------- : 1064
CsAGO6    : LAAQQMGQFIKFEEFSETSSGQK---------------SLTSVGSPAVPELPRLHDN--------------- :  893
CsAGO4c   : LAATQMGQFMKFEDMSETSSSHG---------------GVTSAGAVPVPQLPRLQENSCACSVYSHMASSFS :  860
CsAGO4a   : LAATQISLFMKFEDMSETSSSHG---------------GLTSVGGPPVPELPKLHSN--------------- :  948
CsAGO4b   : LAATQVGQFMKFEDQSETSSSHG---------------GMTSAGARHVIDIARVTK---------------- :  923
CsAGO2/3c : ------------------------------------------------------------------------ :    -
CsAGO10b  : LAAFRARFYMEPDMQENGS-GGGQV----------GKSTRANGDTG-VRPLPALKEN--------------- :  978
CsAGO10c  : LAAYRARFYLEPHVHENAS----------------MRATRTVNGPC-IRPLPALKEK--------------- :  925
CsAGO1    : LAAFRARFYMEPETSDSGSMSSAAVGRAGMMVGPGARSTRVPGANAPVRPLPALKDN--------------- : 1179
CsAGO5c   : LAAFRARYYIEGETSESESTSGG-------------RATRE--RNVEVRPLPLIKDN--------------- : 1011
CsAGO5a   : LAAFRARFYIEGDTSENESTSGG-------------RNTRE--RSIEVRPLPAIKAN--------------- : 1115
CsAGO5b   : LAAFRARYYIEGETLEAGSSSGGGRG--------AGQATRE--RSLEVQPLPQIKDN--------------- :  964
CsAGO2/3e : LVAYRGRMFQEVMMEVSPVSVSSSSSSS---ATSTSSLSVS----SFDDKFYKLHRD--------------- : 1126
            l a

                1520         *      1540         *      1560         *      1580
CsAGO2/3f : ----------------------------------LENIMFFV------------------------------ : 1060
CsAGO2/3d : ----------------------------------LENIMFFI------------------------------ :  916
CsAGO7    : ----------------------------------VKKLMFYC------------------------------ : 1027
CsAGO10a  : ----------------------------------VKRVMFYC------------------------------ :  997
CsAGO2/3a : ----------------------------------VKDTMFFI------------------------------ : 1055
CsAGO2/3b : ----------------------------------LKDTMFFI------------------------------ : 1072
CsAGO6    : ----------------------------------VAGSMFFC------------------------------ :  901
CsAGO4c   : IAMSTWPQRMCSCGYGHCLVKVSRSHKNPGRAYYVCPPAHGTRILDHDSGSTDVMQAEAVEDGGNGDSGGGD :  932
CsAGO4a   : ----------------------------------VCSSMFFC------------------------------ :  956
CsAGO4b   : ----------------------------------VVQIAFCVLFLVPESDAYPLLE---------------- :  945
CsAGO2/3c : ------------------------------------------------------------------------ :    -
CsAGO10b  : ----------------------------------VKRLQQGLQFRSMIIKEQEQ------------------ :  998
CsAGO10c  : ----------------------------------VKNVMFYC------------------------------ :  933
CsAGO1    : ----------------------------------VKRCYLAAVAFWPAVGSPLES----------------- : 1200
CsAGO5c   : ----------------------------------VKDVMFYC------------------------------ : 1019
CsAGO5a   : ----------------------------------VKDVMFYC------------------------------ : 1123
CsAGO5b   : ----------------------------------VKAVMFYC------------------------------ :  972
CsAGO2/3e : ----------------------------------LENIMFFV------------------------------ : 1134
                                                  f

                  *      1600         *      1620         *      1640         *
CsAGO2/3f : ------------------------------------------------------------------------ :    -
CsAGO2/3d : ------------------------------------------------------------------------ :    -
CsAGO7    : ------------------------------------------------------------------------ :    -
CsAGO10a  : ------------------------------------------------------------------------ :    -
CsAGO2/3a : ------------------------------------------------------------------------ :    -
CsAGO2/3b : ------------------------------------------------------------------------ :    -
CsAGO6    : ------------------------------------------------------------------------ :    -
CsAGO4c   : LGDSEAGGDVVEGGEDESGEVVDRAPEFRPDLGFEAEVGGAWESGEAVAPPWKPRRGVVWVDDYPSLLLPAL : 1004
CsAGO4a   : ------------------------------------------------------------------------ :    -
CsAGO4b   : ------------------------------------------------------------------------ :    -
CsAGO2/3c : ------------------------------------------------------------------------ :    -
CsAGO10b  : ------------------------------------------------------------------------ :    -
CsAGO10c  : ------------------------------------------------------------------------ :    -
CsAGO1    : ------------------------------------------------------------------------ :    -
CsAGO5c   : ------------------------------------------------------------------------ :    -
CsAGO5a   : ------------------------------------------------------------------------ :    -
CsAGO5b   : ------------------------------------------------------------------------ :    -
CsAGO2/3e : ------------------------------------------------------------------------ :    -

            1660         *      1680         *      1700         *      1720
CsAGO2/3f : ------------------------------------------------------------------------ :    -
CsAGO2/3d : ------------------------------------------------------------------------ :    -
CsAGO7    : ------------------------------------------------------------------------ :    -
CsAGO10a  : ------------------------------------------------------------------------ :    -
CsAGO2/3a : ------------------------------------------------------------------------ :    -
CsAGO2/3b : ------------------------------------------------------------------------ :    -
CsAGO6    : ------------------------------------------------------------------------ :    -
CsAGO4c   : VRLVCSSDEVVHSSASNTFNPDLPQALGDRGKEGSKLDADRVLKLIPQWSESVEDWNSLIEPLIDKMFAEPS : 1076
CsAGO4a   : ------------------------------------------------------------------------ :    -
CsAGO4b   : ------------------------------------------------------------------------ :    -
CsAGO2/3c : ------------------------------------------------------------------------ :    -
CsAGO10b  : ------------------------------------------------------------------------ :    -
CsAGO10c  : ------------------------------------------------------------------------ :    -
CsAGO1    : ------------------------------------------------------------------------ :    -
CsAGO5c   : ------------------------------------------------------------------------ :    -
CsAGO5a   : ------------------------------------------------------------------------ :    -
CsAGO5b   : ------------------------------------------------------------------------ :    -
CsAGO2/3e : ------------------------------------------------------------------------ :    -

              *      1740         *      1760         *      1780         *      1800
CsAGO2/3f : ------------------------------------------------------------------------ :    -
CsAGO2/3d : ------------------------------------------------------------------------ :    -
CsAGO7    : ------------------------------------------------------------------------ :    -
CsAGO10a  : ------------------------------------------------------------------------ :    -
CsAGO2/3a : ------------------------------------------------------------------------ :    -
CsAGO2/3b : ------------------------------------------------------------------------ :    -
CsAGO6    : ------------------------------------------------------------------------ :    -
CsAGO4c   : NAIIVRWENQNYEVDNPLKLEHSLFDRLCPLLIIRLLPLRVFNDLNSSLIYGELCKQDTGYFDIHDAESVAG : 1148
CsAGO4a   : ------------------------------------------------------------------------ :    -
CsAGO4b   : ------------------------------------------------------------------------ :    -
CsAGO2/3c : ------------------------------------------------------------------------ :    -
CsAGO10b  : ------------------------------------------------------------------------ :    -
CsAGO10c  : ------------------------------------------------------------------------ :    -
CsAGO1    : ------------------------------------------------------------------------ :    -
CsAGO5c   : ------------------------------------------------------------------------ :    -
CsAGO5a   : ------------------------------------------------------------------------ :    -
CsAGO5b   : ------------------------------------------------------------------------ :    -
CsAGO2/3e : ------------------------------------------------------------------------ :    -
```

```
                     *      1820         *      1840         *      1860         *
CsAGO2/3f : ------------------------------------------------------------------------ :    -
CsAGO2/3d : ------------------------------------------------------------------------ :    -
CsAGO7    : ------------------------------------------------------------------------ :    -
CsAGO10a  : ------------------------------------------------------------------------ :    -
CsAGO2/3a : ------------------------------------------------------------------------ :    -
CsAGO2/3b : ------------------------------------------------------------------------ :    -
CsAGO6    : ------------------------------------------------------------------------ :    -
CsAGO4c   : ILLNRAFNKFEFEDVRKLSAELCGRIHPQVLFPLIASQLEHAVDARDALKIKACLFSICTSLVARGRDSLLH : 1220
CsAGO4a   : ------------------------------------------------------------------------ :    -
CsAGO4b   : ------------------------------------------------------------------------ :    -
CsAGO2/3c : ------------------------------------------------------------------------ :    -
CsAGO10b  : ------------------------------------------------------------------------ :    -
CsAGO10c  : ------------------------------------------------------------------------ :    -
CsAGO1    : ------------------------------------------------------------------------ :    -
CsAGO5c   : ------------------------------------------------------------------------ :    -
CsAGO5a   : ------------------------------------------------------------------------ :    -
CsAGO5b   : ------------------------------------------------------------------------ :    -
CsAGO2/3e : ------------------------------------------------------------------------ :    -
```

```
                1880         *      1900         *      1920         *      1940
CsAGO2/3f : ------------------------------------------------------------------------ :    -
CsAGO2/3d : ------------------------------------------------------------------------ :    -
CsAGO7    : ------------------------------------------------------------------------ :    -
CsAGO10a  : ------------------------------------------------------------------------ :    -
CsAGO2/3a : ------------------------------------------------------------------------ :    -
CsAGO2/3b : ------------------------------------------------------------------------ :    -
CsAGO6    : ------------------------------------------------------------------------ :    -
CsAGO4c   : PIVLKIRNTIETILLWPSSNGDEVSKAQHGCIDCLALMICTELQALESFRDLTSKNTSLVRMDSSRDAAMRD : 1292
CsAGO4a   : ------------------------------------------------------------------------ :    -
CsAGO4b   : ------------------------------------------------------------------------ :    -
CsAGO2/3c : ------------------------------------------------------------------------ :    -
CsAGO10b  : ------------------------------------------------------------------------ :    -
CsAGO10c  : ------------------------------------------------------------------------ :    -
CsAGO1    : ------------------------------------------------------------------------ :    -
CsAGO5c   : ------------------------------------------------------------------------ :    -
CsAGO5a   : ------------------------------------------------------------------------ :    -
CsAGO5b   : ------------------------------------------------------------------------ :    -
CsAGO2/3e : ------------------------------------------------------------------------ :    -
```

```
                 *      1960         *      1980         *      2000         *
CsAGO2/3f : ------------------------------------------------------------------------ :    -
CsAGO2/3d : ------------------------------------------------------------------------ :    -
CsAGO7    : ------------------------------------------------------------------------ :    -
CsAGO10a  : ------------------------------------------------------------------------ :    -
CsAGO2/3a : ------------------------------------------------------------------------ :    -
CsAGO2/3b : ------------------------------------------------------------------------ :    -
CsAGO6    : ------------------------------------------------------------------------ :    -
CsAGO4c   : SVRSYVIHQLTRDKDCTYEASISMSFVLCMANVLISACQKIPDSCKRPFAREILPRLIQSVEVMMESEYRAA : 1364
CsAGO4a   : ------------------------------------------------------------------------ :    -
CsAGO4b   : ------------------------------------------------------------------------ :    -
CsAGO2/3c : ------------------------------------------------------------------------ :    -
CsAGO10b  : ------------------------------------------------------------------------ :    -
CsAGO10c  : ------------------------------------------------------------------------ :    -
CsAGO1    : ------------------------------------------------------------------------ :    -
CsAGO5c   : ------------------------------------------------------------------------ :    -
CsAGO5a   : ------------------------------------------------------------------------ :    -
CsAGO5b   : ------------------------------------------------------------------------ :    -
CsAGO2/3e : ------------------------------------------------------------------------ :    -
```

```
            2020         *      2040         *      2060         *      2080
CsAGO2/3f : ------------------------------------------------------------------------ :    -
CsAGO2/3d : ------------------------------------------------------------------------ :    -
CsAGO7    : ------------------------------------------------------------------------ :    -
CsAGO10a  : ------------------------------------------------------------------------ :    -
CsAGO2/3a : ------------------------------------------------------------------------ :    -
CsAGO2/3b : ------------------------------------------------------------------------ :    -
CsAGO6    : ------------------------------------------------------------------------ :    -
CsAGO4c   : CLQVFFSAVYHLKSTILPYTSDLLRVSLKSLREGSEKEKMAGAKLMASLMASEEAIVNSIAGGLLEAITVLS : 1436
CsAGO4a   : ------------------------------------------------------------------------ :    -
CsAGO4b   : ------------------------------------------------------------------------ :    -
CsAGO2/3c : ------------------------------------------------------------------------ :    -
CsAGO10b  : ------------------------------------------------------------------------ :    -
CsAGO10c  : ------------------------------------------------------------------------ :    -
CsAGO1    : ------------------------------------------------------------------------ :    -
CsAGO5c   : ------------------------------------------------------------------------ :    -
CsAGO5a   : ------------------------------------------------------------------------ :    -
CsAGO5b   : ------------------------------------------------------------------------ :    -
CsAGO2/3e : ------------------------------------------------------------------------ :    -
```

```
             *      2100         *
CsAGO2/3f : ------------------------- :    -
CsAGO2/3d : ------------------------- :    -
CsAGO7    : ------------------------- :    -
CsAGO10a  : ------------------------- :    -
CsAGO2/3a : ------------------------- :    -
CsAGO2/3b : ------------------------- :    -
CsAGO6    : ------------------------- :    -
CsAGO4c   : SLSSSDPSPDVRQVSEKLLACLTSL : 1461
CsAGO4a   : ------------------------- :    -
CsAGO4b   : ------------------------- :    -
CsAGO2/3c : ------------------------- :    -
CsAGO10b  : ------------------------- :    -
CsAGO10c  : ------------------------- :    -
CsAGO1    : ------------------------- :    -
CsAGO5c   : ------------------------- :    -
CsAGO5a   : ------------------------- :    -
CsAGO5b   : ------------------------- :    -
CsAGO2/3e : ------------------------- :    -
```

Supplementary Figure S1-b: Multiple Sequence alignment of CsDCL proteins: The names of the identified CsDCL genes are indicated to the left of the alignments. The length of the alignment is shown on the top, while the length of each protein is given on the right. Conserved consensus residues are shown at the bottom. Invariable residues conserved at 100% are highlighted in red, those conserved at >80% are shown in yellow and residues that are >60% conserved are highlighted in green. The sequences were aligned usi9ng ClustalX and viewed by GeneDoc.

```
                   *        20         *        40         *        60         *
CsDCL1a : ---------------------------MRKEICEIQKVRGSGKSSSGKDRELKKLHCTVNYDVQEGSGPLPGKG :   47
CsDCL1b : -------------------------------------------------------------------------- :    -
CsDCL2  : MDNCISLEDDNNSSVSNNPTVGNNPISEGMELVERTMSGTQQMSTDPLPFARSYQIEALDKAIKQNTIVFLETG :   74
CsDCL3  : MHSPDEAEAVKPLKRSFEIMNSKQSDPIEMDIAPPPSTTTSSSLTPHNFRPRGYQLKVFEVAMRRNTIAVLETG :   74
CsDCL4  : -------------------------------------------------------------------------- :    -

              80         *       100         *       120         *       140
CsDCL1a : GEEDMVGCLVKDSKIPKK-------------AAGAFIVFMGVDCAIKIRNLETKLDS--------VVCTIKDRK :  100
CsDCL1b : -------------------------------------------------------------------------- :    -
CsDCL2  : SGKTLIAIMLLRSYAYLLRKPSP--YIAVFLVPNVVLVTQQAEAIKMYTDLEVGMYWGEMGVDYWDGATWKEQQ :  146
CsDCL3  : AGKTMIAVMIINDMAPSLNLVPPHKNIIVFLAPTVHLVHQQFEVMKVHTNLQVEEYYGAKGVDEWNAKSWEKEI :  148
CsDCL4  : -------------------------------------------------------------------------M :    1

           *       160         *       180         *       200         *       220
CsDCL1a : ELEKHVPMPSEIVVEYDKATSLWSLHEQIKQMELAVEEAA--QSSSRRSKWQFMGARDAGAKEELRQVYGVSER :  172
CsDCL1b : -------------------------------------------------------------------------- :    -
CsDCL2  : DNHEVLVMTPMILLNALRHSFLRLDMIKVLIFDECHNARG--RHPYVCIMTEFYHYQLRSNSSQLPRIFGMTAS :  218
CsDCL3  : NDHDVLVLTPQILLDALRKAFVSFEMVCLMIIDECHRATG--NHPYTKIMKEFYHKSRNK-----PKIFGMTAS :  215
CsDCL4  : EEYEVFVMTPQLLFRKLCHCFIKIELIALLIFDECHHAQLNSDHPYAEIMKVFYKNDASK----LPRIFGMTAS :   71
                                                              f                g

                 *       240         *       260         *       280         *
CsDCL1a : TESDGAAN---LIQKLRAINYALGEIGQWCAYKVAQS------------------------------------- :  206
CsDCL1b : -------------------------------------------------------------------------- :    -
CsDCL2  : PIKAKGSS---SGSTYWKQILELENLMNSKVYTVVSESVLAKYIPFSTPKLKIYKHVEIP----YAIFEHLVHD :  285
CsDCL3  : PVIRKGVS---SLTDCEEQISELEGILDSQIYTVEDRTELEEIIPSAEEICRFYDPAVSLNMDLKAKLESSWKK :  286
CsDCL4  : PKLGKGNTSCCHVTISGASINTLETLLHSKVYSVEDKEELEQFVASPKFKIYYYGPCVSN----SSSPHMIYYK :  141
                                      l        y v

           300         *       320         *       340         *       360         *
CsDCL1a : -------FLTALQNDERSNYQLDVKFQESYLSKVVTLLQCQLSEGAVSENNSKNATADNDATQGGSGLDEIEEG :  273
CsDCL1b : -------------------------------------------------------------------------- :    -
CsDCL2  : LTCLKEKHEFTLKASNLEEHAMDNAMKK-LSKLFSSFLFCLNELGVWLALKAAESLSCEENDIFSWGKLDVCSE :  358
CsDCL3  : FDALLLELQGSISSQYRDTEDKYTALRKRLSNDNAKILYCLDDLGLLCAYEAVKVCIENSSNATAECEYYRKSS :  360
CsDCL4  : NLEEIKHQFVSTLSTKTDDLGSLKSTKKMLWRLHNYLIFCLENLGVWGAFQACDILLKGDCPERNELIAAEGNG :  215
                                                 c    g

                 380         *       400         *       420         *       440
CsDCL1a : ELPSSHVVSGGEHVDVII--------------GAAVADGKVTPKVQSLIKILLKYQ-------HTVDFRAIIFV :  326
CsDCL1b : -------------------------------------------------------------------------- :    -
CsDCL2  : TVVRDFSLDAFKVFSNYVPSGP--HWSIGDDINANMDAGYISSKVICLIESLLDYR-------DLNNLRCIIFV :  423
CsDCL3  : LQCKYFLEEVLSTMTASLPHGNKNLLGIGSDCSKTVAMGYISPKLYELFQIFQSFG-------EARQVLCLIFV :  427
CsDCL4  : DDISIWNRYLAQAASVFARDCKTDGIEADLSCVEVLKEPLFSKKLLQLIGILSTFRSSFWAEVYVPEKYEYHFG :  289
                                                     k   l                        f

               *       460         *       480         *       500         *       5
CsDCL1a : ERVVAALVLPKVFAELP-SLSFVKSASLIGHN--------------NSREMRTSQMQD-TIGKFRDGRVTLLVA :  384
CsDCL1b : -------------------------------------------------------------------------- :    -
CsDCL2  : ERVISAIVLQKLLSELLPKLGGWKTKYTAGNQ--------------SGFQSQSRKEQNKIVEEFRKGTVNIIVA :  483
CsDCL3  : ERIITAKVIERVMKRTD-YLSHLTVSYLTGSN--------------SSVDALAPKVQKETLESFRSGKVNLLFA :  486
CsDCL4  : KVPVWQGVHSGLKCMSRKNMNIILEKFRSGKQELIIDLKELGLEFGTSLGPTSMRFRETEFDKFLIWQLNLLVA :  363
                 v                     g                                 f         a

          20         *       540         *       560         *       580         *
CsDCL1a : TSVAEEGLDIRQCNVVIRFDLAKTVLAYIQSRGRARKPGSDYILMVERGNLSHEAFLRNARNSEVTLRKEAVER :  458
CsDCL1b : -------------------------------------------------------------------------- :    -
CsDCL2  : TSILEEGLDVQSCNLVIRFDPSATVCSFIQSRGRARMQNSDFLLMVRSGDSSTLTRVKNYIASGDIMREESLRH :  557
CsDCL3  : TDVVEEGIHVPNCSSVIRFDLPKTVRSYVQSRGRARQNDSHFVMMLERGNIKQRDHIFDIIRSEYSMTDTAINR :  560
CsDCL4  : TKVGEEGLDIQTCCLVIRFDLPETVASFIQSRGRARMPQSEYAFLVDRGNQNELNLIEDFKKEEDKMNAEIASR :  437
          t   eeg     c  virfd   tv    qsrgrar   s        g

               600         *       620         *       640         *       660
CsDCL1a : TDLSHLKGTSRLISVDTTPGTVYQVESTGAVVSLNSAVGLIHFYCSQLPSDRYSILHPEFIVERHEMPGGPIEY :  532
CsDCL1b : -------------------------------------------------------------------------- :    -
CsDCL2  : ASL-----PCASLDSELFNEVLYRVQSTGAVVTLSSSVALIYFYCSRLPSDGYFKPSP--RCEIDKDL-----E :  619
CsDCL3  : DPD-----ASVLKACTIKEIDAYYVDATGASVTVDSSVNLIHRYCEKLPRDEYFTPKPIFQFILSGES-----Y :  624
CsDCL4  : TSS---------VTFVDLEEKTYKVASTGATISSGYSISLLHHYCSKLPHDEYFNPKPEFSCFDDVEG-----T :  497
                                y v  tga         l   yc  lp d y    p

             *       680         *       700         *       720         *       740
CsDCL1a : SCRLQLPCNAPFEKLEGPVCSSMRLAQQACWHHVINAYKGSGEGEKVDQNDEGDPLPGTARHREFYPEGVANIL :  606
CsDCL1b : -------------------------------------------------------------------------M :    1
CsDCL2  : ICTLNFPKSCPVQTVR-----------------------------------------------------VEGNI :  640
CsDCL3  : QCNLTLPPNAAFQTLVG---------------------------------------------------PPSRNS :  647
CsDCL4  : VCSIILPSNAPIHQISS---------------------------------------------------LPQSSM :  520
           c    p

                   *       760         *       780         *       800         *
CsDCL1a : QGDWILSGRDACYNSKLFHLYMYDIECVDIGSLQDPFLTQVLEFAVVFGNELDAEVLSMSMDLFIARTMITKAS :  680
CsDCL1b : QGEWILSGRDACYNSKLFHLYMYDIECVDIGSSKDPFLTQVLEFAVVFGNELDAEVLSMSMDLFIARTMITKAS :   75
CsDCL2  : KVLKQLVCLEACKQLHQKGALTDNLVPDILMEEADE-----QESGYEPYVDDQVEYFPPELVHNCTNDS-VRTY :  708
CsDCL3  : HLSKQLVCFDACKKLHQMGALNDHLLPFNEEPSVNDSTLKSKRSTSSAGTTKRKELHGTTCVHALSGTWGLKPD :  721
CsDCL4  : EAAKKDACLKACKELHEKGALNEYLLPDE-DDENEELMQDFSGSDSCDDEASRRELHEMRIPAALKEPWTDLEN :  593
               l    AC            6                             E

             820         *       840         *       860         *       880
CsDCL1a : LVFRGSIDITESQLASLKSFHVRLMSIVLDVDVEPSTTPWDPSKAY-LFVPVVGDNSVDPLKEIDWYLVEKISK :  753
CsDCL1b : LVFRGSIDITESQLASLKSFHVRLMSIVLDVDVEPSTTPWDPSKAY-LFVPVVGDNSVDPLKEIDWDLVEKISK :  148
CsDCL2  : HRYLIVLKRNFEYNIP---LHPIVLAVRTKLEFSDENLACELETDR-GTLKVQMEYIGELSLTSEQVLLCQRFE :  778
CsDCL3  : GTVFQAYRIHFTCNIPGVHYSSFVLLIDSKLDDDVGNVEMELFLLS-KFVKSSVSSCGEIHLDAEQVVRAMCFQ :  794
CsDCL4  : PVYLNSYFIKLSPDPADRFYKNFALFVKAPLPREAERMKLDLFLARGRLVMTELVPSGVAKFDKDEILQAQNFQ :  667
                  i               6 6   6                  6                 6
```

```
               *       900         *       920         *       940         *       960
CsDCL1a : TDVWNNPLQRARPDVYLGTNERTLGGDRREYGFGKLRHGMAFGQKSHPTYGIRGAVAQFDIVKASGLVPNRNAF :  827
CsDCL1b : TDAWNNPLQRARPDVYLGTNERTLGGDRREYGFGKLRHGMAFGQKSHPTYGIRGAVAQFDIVKASGLVPNRNAI :  222
CsDCL2  : ITVFRVLLDHNLKKLK------ELLAFHSSNDLAVLDYLVLPCTSSHQNPS----IDWNCVSSVVFPSKILGHN :  842
CsDCL3  : EFFFNGIFGKLFVRLKSSRGGRKFLLQGESLWEPSNMYLLPLESSNVSSDEPWRINWMGINSCVSVVEFLKEN :  868
CsDCL4  : EMYLKVILDRSEFISEFVS----LGKNGFSGSGSPTFYLLPVILCEYKSTMT--VDWKLIRRCLSSPIFRSRQ :  735
                  l               l               6     s              6   6

               *       980         *      1000         *      1020         *
CsDCL1a : EIGNHLDLT--------------------KGKLMMADSFIGVEDLVGRIVTAAHSGKRFYVDSVRYDMTAENSF :  881
CsDCL1b : EIGNHLDLT--------------------KRKLMMADSFIGVEDLVGRIVTAAHSGKRFYVDSVRYDMTAENSF :  276
CsDCL2  : HMNTTPNSS--------------------CRQVRTKNGLVCSCMLENSLVYTPHND-YVYSIAGTLD------- :  888
CsDCL3  : AWLSAEQSSGDRGNSSVHGTNSIESECKATNVIHLANKSIGTKNLREMVVLAIHTG-RIYSVLRAVDNTSAESP :  941
CsDCL4  : DARDSVIPHLN-------------------NHLELANGPHIINDVLNSLVYAPCKDTFFISDVVPE------- :  783
                                       6  a1      6   6V a h      5     d

          1040         *      1060         *      1080         *      1100         *
CsDCL1a : PRKEGYLGPLEYSSYADYYRQKYGVELIYKQQPLIRGRGVSYCKNLLSPRFEHSEAHDGE-----SEEALDKTY :  950
CsDCL1b : PRKEGYLGPLEYSSYADYYRQKYGVELIYKQQPLIRGRGVSYCKNLLSPRFEHSEANEGE-----SEEALDKTY :  345
CsDCL2  : LHGNS-LLEGKCITYKQYYKSKYGIDLHFGRESLLNGRHIFTVQNYLHRCRQ-------------KKERESRNA :  948
CsDCL3  : FDGNSDAIPSSYSSFAEYFSKKYGITLMHPGQPLLLLKQSHNAHNLLVDFRNEGASFGKNLEAVNKMAVEKPRN : 1015
CsDCL4  : --KNGYSPFNDSTNHIEHYITLFNDGSGPRCRHWYETSGMAVMDDYHKEWLSG-------------------- :  834
                            y5  k5g  l      l         1 1

                1120         *      1140         *      1160         *      1180
CsDCL1a : YVFLPPELCFVHPLPASLVRGAQR--LPSIMRRVESMLLAIQLKDIINYPVPA-SKILEALTAAS-----CQEP : 1016
CsDCL1b : YVFLPPELCFVHPLPGSLVRGAQR--LPSIMRRVESMLLAIQLKDIINYPVPA-SKILEALTAAS-----CQET :  411
CsDCL2  : YVELPPELCFVFMSPISIATVYSLSFVPSIMHRIECLLIALNLKKMHLDHCMQ-NVVIP-------------- : 1006
CsDCL3  : HVHMPPELLVSIDVPINVLKSFYL--LPSLMYRLESLMLASQLREEIACHSSN-IRIPSSLILEAITTLRCNEV : 1086
CsDCL4  : -----------------NGPNSLSLLPSIMHRLESFLVAIELKDRLSASFPEGAEVTAYRVLEALTEKCNEK :  890
           v  ppel      p          6PS6M R6Es 66A  L4           6           c e

                *      1200         *      1220         *      1240         *      12
CsDCL1a : FCYERAELLGDAYLKWVVSRFLFLKYPRKHEGQLTRLRQQMVSNMVLYQYALNKGLQSYIQADRFAPSRWAAPG : 1090
CsDCL1b : FCYERAELLGDAYLKWVVSRFLFLKYPQKHEGQLTRMRQQMVSNMVLYQYALNKGLQSYIQADRFAPSRWAAPG :  485
CsDCL2  : ------------------------------------------------------TSKGFIRNEPFDPKTWIVPG : 1026
CsDCL3  : FSMERLELLGDSVLKYSVSCHLFLKYPKKHEGQLSARRSWAVCNSTLHKLGTDRNLQGYIRDGAFEPRRWIAPG : 1160
CsDCL4  : FSLERLEVLGDAFLKFAVGRHLFLLHDTLDEGWLTRKRSNIVNNSNLLKLATMSKLQEYIRDQSFDPHQFFTLG :  964
          f  er e lgd  lk  v   lfl      eg l   r   v n   l        lq 5I     F P  5  pG

          60         *      1280         *      1300         *      1320         *
CsDCL1a : VLPVFDEDIKETGSSLFDDETALGVTVPGEAQDADGYEDDEMEDGELESDSSSYRVLSSKTIADVVEALIGIYY : 1164
CsDCL1b : VLPVFDEDIKETGSSLFDNERALGVTVPGEAQGADGYEDDEMEDGELESDSSSYRVLSSKTIADVVEALIGIYY :  559
CsDCL2  : DQ-------------------------------SGDYALDEESLSSTRKIYIRGRRKMKSKTVADVVEALIGAFL : 1070
CsDCL3  : QRSIHPCPCSCG--------------VDTLEVPLDIKFQTEDAKVVGKCCDRGHRWMGSKTISDCVEALIGAYY : 1220
CsDCL4  : RPCPVICSTKT-----------------EAIIHSPQENSLTNDANVEVRCTRGHHWLHKKTISDVVEALIGAFI : 1021
                                     d      e                 r 6 sKT6 DvVEALIG 5

             1340         *      1360         *      1380         *      1400
CsDCL1a : VEGGKNAANHLMKWIGIQIDFDPKELECTTRPSNVPESILRSINFDALEGALNINFKDRGSLVEAITHASRPSS : 1238
CsDCL1b : VEGGKKAANNLMKWIGIQIDFGPKELECTTRPSNVPESILRSINFDALEGALNIKFEDRGLLVEAITHASRPSS :  633
CsDCL2  : STGGEASALSFMDWLGIKVDFVNIPYERHFPVQAER-----LVNIKYFESLLNYSFRDPSLLVEALTHGSYMLP : 1139
CsDCL3  : VGGGLTAALQLMKWLGVDAELEPSLVDEAIKVASLHSYIPKAKEIETLESKLGYEFSIRGLLLEAITHASEQEL : 1294
CsDCL4  : VDSGFKAAATAFLKWIGIQVDFEASQVSNICSASTIFKTLDAHMDITKLENLLGYQFLHKGLLVQAFVHPSYKKY : 1095
          v gG  aA   6kW6G6  df                         lE  L   F   glL62A tH S

            *      1420         *      1440         *      1460         *      1480
CsDCL1a : GVS-CYQRLEFVGDAVLDHLITRHLFFTYSDLPPGRLTDLRAAAVNNENFARVAVKHKLHMHLRHGSSALEKQI : 1311
CsDCL1b : GVS-CYQRLEFVGDAVLDHLITKHLFFTYTDLPPGRLTDLRAAAVNNENFARVAVKHKLHLHLRHGSSALEKQI :  706
CsDCL2  : EIPGCYQRLEYLGDSVLDFLITMHLYHKYPGMSPGLLTDLRSASVNNDCYAQSAVKFGLHKHILHASQSLHKDI : 1213
CsDCL3  : GVGYCYQRLEFLGDSVLDILITWHLYQSHTDIDPGELTDLRSASVNNENFANVAVKKNLYQHLQHRSGLLNQI : 1368
CsDCL4  : WGG-CYQRLEFLGDAVLDYLITSYLYSVYPKLNPGQLTELRSLSVCNNSFAKIAVDLSFHEFMLCNSSSLHASM : 1168
               CYQRLE56GD VLD LIT hL5  y  6 PG LTdLR a VnN  5A  AVk  lh h6 h S  L  6

                    *      1500         *      1520         *      1540         *
CsDCL1a : RDFVKEVKDELSKPGFNS--------------------------FGLGDCKAPKVLGDIVESIAGAIFLDSGR- : 1358
CsDCL1b : RDFVKEVQDELSKPGFNS--------------------------FGLGDCKAPKVLGDIVESIAGAIFLDSGR- :  753
CsDCL2  : VATVQNFEQLSVDTTFG--------------------------WESETTFPKVLGDIIESLAGAILVDSGYN : 1259
CsDCL3  : TEYVKFISGSQRTTQP-------------------------------LQGGKGLKTLGDLVESIAGAILIDTKLN : 1412
CsDCL4  : NKYVNFIRTTALEKGFVEGPSCPKYGYGYGYGYLNGCTCTGYAKYVCEILGNDMGTFVGDLVESCVGAILLDTGFD : 1242
            V           f                            k 6GD66ES aGAI 6D3g

           1560         *      1580         *      1600         *      1620
CsDCL1a : ---DTAVFQPLLHPMVTPETLPMHPVRELQERCQQQAEGLEYKASRNGNLATVEVFIDG---VQVGIAQNPQKK : 1426
CsDCL1b : ---DTAVFQPLLHPMVTPETLPMHPVRELQERCQQQAEGLEYKATRSGNLATVEVFIDG---VQVGIAQNPQKK :  821
CsDCL2  : KEVVLRSIRPLLEPMITPETVKLHPVRELTELCSQKHFVMEKAVSRDKGVASITIKVEANGVYKSTSTASDKK : 1333
CsDCL3  : LDEVWRIFEPLLSPIVTPDKLELPPLRELIELCDSLGYFIKENCIKKGGMVHAELRLQLKDVLLVGEGLGHNRK : 1486
CsDCL4  : LNRVWKIVLSFMDPVMSFSKFQLNPKQELQKLCDFHSWKPEFPYLKKNGVFVVEAKVKAKNVWITACATNINKT : 1316
                 pl6 P663p    6 P rEL e C        e    4    6    e  6                4k

           *      1640         *      1660         *      1680         *      1700
CsDCL1a : MAQKLAARGMPLCVSEGGPAHAKR-------FTFAVRVNTTDRELTEECIGEPMPSVKKAKDSAAVLLELLNK : 1493
CsDCL1b : MAQK--------CVSEGGPAHAKR-------FTFAVRVNTTDRGWTDECIGEPMPSVKKAKDSAAVLLELLNK :  880
CsDCL2  : TAKKLAAKAVLKSLKEG--------------------MTGACEQQNKACSSNFR------------------------ : 1367
CsDCL3  : VAKGQAALHLLKDLESRGISYSKRRKQCPDGVGVQSTLHLDNNICSQTNADPSQPKAQKKQKTTVTQLDSVPNK : 1560
CsDCL4  : AAQRIAAQKLYGNISISMAESSEQDLDSNFAGLRRSHLNVFDRQRLVRFPSNRSFAGYKLKSKSLEEVLKSSLK : 1390
           A   aa       6                       1                   k              k

                  *      1720         *      1740         *      1760         *
CsDCL1a : LYP----------------------------------------------------------------------- : 1496
CsDCL1b : LYP----------------------------------------------------------------------- :  883
CsDCL2  : -------------------------------------------------------------------------- :    -
CsDCL3  : NPYHSDDCSSKSCDIPGAFKKRNSLGNLVVSLEEINYLKLDGNYSILCIGMFSAKSARLRRRLIFLGDRSYEFE : 1634
CsDCL4  : MEAKLIGHDEASIDVTAPDAIPLDN------LKLQESSRSQCNLKVHPVSQEPKKTFHPRIRSITEPQPSLEAQ : 1458

          1780         *      1800         *      1820         *      1840         *
CsDCL1a : -------------------------------------------------------------------------- :    -
CsDCL1b : -------------------------------------------------------------------------- :    -
CsDCL2  : -------------------------------------------------------------------------- :    -
CsDCL3  : KGLADAIGRRHVVDIVERGQGVRQAIELFGA-EIKWVADQMRKASKMNTVGVFLGRVSRGQRSVKRDGRALVME : 1707
CsDCL4  : GPQSTAGGDHNLVGGSCKKSAKSRLHEICAANCWKPPSFKCCKEIGPSHLKEFIFKVSVDIEEASDVIAAFGAP : 1532

                1860         *
CsDCL1a : --------------------------- :    -
CsDCL1b : --------------------------- :    -
CsDCL2  : --------------------------- :    -
CsDCL3  : HRDLVRSAAHDGYRDLGKMKTQSQIS- : 1733
CsDCL4  : HSRKKDAAEHAAEGALWYLKNAGYLHD : 1559
```

Supplementary Figure S1-c: Multiple Sequence alignment of CsRDR proteins: The names of the identified CsRDR genes are indicated to the left of the alignments. The length of the alignment is shown on the top, while the length of each protein is given on the right. Conserved consensus residues are shown at the bottom. Invariable residues conserved at 100% are highlighted in red, those conserved at >80% are shown in yellow and residues that are >60% conserved are highlighted in green. The sequences were aligned usi9ng ClustalX and viewed by GeneDoc.

```
                   *        20         *        40         *        60         *
CsRDR1c : -----------------------------------------------------------------------MAI :    3
CsRDR1a : -------------------------------------------------------------------------- :    -
CsRDR1b : -------------------------------------------------------------------------- :    -
CsRDR2  : -------------------------------------------------------------------------- :    -
CsRDR5a : -------------------------------------------------------------------------- :    -
CsRDR5b : -------------------------------------------------------------------------- :    -
CsRDR6a : -------------------------------------------------------------------------- :    -
CsRDR6b : MPLLNLTATCYTAGTQFRRWDTEKETREKDEERDAKQNEEGSEGFGGINHNTNTKMREVFAAVLTLTLTVAVLS :   74
CsRDR6c : -----------------------------------MMRESGS-------------------------------- :    7

              80         *       100         *       120         *       140
CsRDR1c : AVAVAMVMSDGGKHWRPPTLGWLAGVRIGGDYSVCVKSRLDSMGKTIQLSGFPHLVSAERVKEFLEKHTG---- :   73
CsRDR1a : ------------------------------------------MGKTIDLAGFHCLVSEETVKELVEQYTG---- :   28
CsRDR1b : ------------------------------------------MGKTIHLSGFPYLVSAERVKEFLEQHTG---- :   28
CsRDR2  : ---------------------------------------MAVDRPTVRVSNIPQTAIAKDLFDFFESTIG---- :   31
CsRDR5a : -------------------------------------------------------------------------- :    -
CsRDR5b : -------------------------------------------------------------------------- :    -
CsRDR6a : -------------------------------------------------------------------------- :    -
CsRDR6b : LPLSPVGKCLVNVVLVANMTLPSWKCPCLLGGMGSEGSENEMVVTQISVGGFGNYVTAKMLFDYFEDQTGGVWR :  148
CsRDR6c : --------------------------IHIKGGMGSEGSENEMVVTQISVGGFGIYVTAKMLFDYFEDQIGGVWR :   55

           *       160         *       180         *       200         *       220
CsRDR1c : --------------KGTVYALEVREPKKRG-SRTYATVQFTSAKSSEHIISLANQR-LYYGSSYLKAYAKDFD- :  130
CsRDR1a : --------------TGTVYLVQVKEPRKKE-SRVYARVQFTTVENADLIISLADERKIYYETSYLKAYPKEFD- :   86
CsRDR1b : --------------RGTVVALEVKEPKRKG-SRAFATVQFATASSADYIISLASRR-LYYGSSYLKAYPKDFD- :   85
CsRDR2  : --------------KGSVYACEIVSDRKNWKSKGFGRVQFETLDSKSNATRLSQEGKLVFKGFVLFLSASFDD- :   90
CsRDR5a : -------------------------------------------------------------------------- :    -
CsRDR5b : -------------------------------------------------------------------------- :    -
CsRDR6a : -------------------------------------------------------------------------- :    -
CsRDR6b : CRLKTSSTPPESYPNFLIDTADIQRKDDNHQVEPHAFLHFISPDSAKLALDAAGRGELVLMKNHLKVSLGPDNP :  222
CsRDR6c : CRLKTSSTPPESYPNFLIDSADIRRTNDYHKVEPHAFLHFISPDSAKLALDAAGRGELVLMKKHLKVSLGPDNP :  129

                 *       240         *       260         *       280         *
CsRDR1c : ---IVQKPKVYVHDMESVTLYFGCQISKEKFSVLWKKANVSVKFGFG--LRKLYFFLSYLSVEYKLELSYENIW :  199
CsRDR1a : ---IERERKVYVHDMEAETLYFGCQTSKDMFSVLWKSENVPVEFWFH--RRKLRFFLSYLSVEYKLELLYENIW :  155
CsRDR1b : ---LVQT-KAYVHDMESVTLYFGCQISKEKFSVLWKRENVSVKFGFG--LRKLYFFLSHFFVEYRLDLFYENIW :  153
CsRDR2  : ---VIARPVEPSNRVENGVLHCGLMVKHDCMSVLESFDGVKAWVMPE--RKSLEFWVVHSGECYKLDLQFDYVL :  159
CsRDR5a : -------------------------------------------------------------------------- :    -
CsRDR5b : -------------------------------------------------------------------------- :    -
CsRDR6a : -------------------------------------------------------------------------- :    -
CsRDR6b : HSMNQRRRNTVPHKLSDVRLEIGILVSRDEFFVGWRGPLTGVDFVVDPFNETCEFLFTRDTVFSLKNSNKHSIM :  296
CsRDR6c : HSMNQRRRNTVPHKLSDVRLEIGNLVSRDEFFVGWRGPPTGVDFVVDPFNETCEFLFTRDTVFSFKNSNKHSVM :  203

           300         *       320         *       340         *       360         *
CsRDR1c : QIELRRLQG------------------------QTAKF------LLIQLLGAPRIYEKYEGS---------VLN :  234
CsRDR1a : QIELRCPPD------------------------KSEKF------LLIQLLGACQIYKKCEES---------ADS :  190
CsRDR1b : QIELRRPRD------------------------KTSKF------LLIQLVGAPRIYKKVEES---------IYS :  188
CsRDR2  : ETKGCRLDSEKPNAVLLKGRIAAQVAKPGTTLAQPDKFGASIGALVHKLKHAPKIYQKISGPNVNSKFGANRYH :  233
CsRDR5a : -------------------------------------------------------------------------- :    -
CsRDR5b : -------------------------------------------------------------------------- :    -
CsRDR6a : -------------------------------------------------------------------------- :    -
CsRDR6b : KCNFKVEFLVRDIN------------------EIKQYADMSSLIVVMQLASSPSVHYRTADYD-----IEESVP :  347
CsRDR6c : KCNFKVEFLVRDIN------------------DIKQYTDMFSLIVVMQLASSPSVYYRTADDD-----IEESVP :  254

                 380         *       400         *       420         *       440
CsRDR1c : FFKEIPDDQWVRSTDFTPSHCIGHSSALCLELPSWVPLLNFSDYFAYYRENEGPFVLKSGSTFSQNLDLVPIMG :  308
CsRDR1a : YSKETPEYQWVRETDFTPLFCLGQSSAICLELPSGVPVLNFSEYFAYYKENEGPFILESGLPFSCNLDLVPIVG :  264
CsRDR1b : YFKETSDDQWVRETDFTPSNCIGQSSSLCLELPCGVDLLNFSEYFAFYKENEGPFILERGLTFSCNLDLVPIVG :  262
CsRDR2  : ICKEDFGFLWVRTTDFSGMKSIGQSSSLCWEINEELSTFDNFTSLPYYTKDVKDLILEEGEEFGSTSEVVPLVS :  307
CsRDR5a : -------MQNLVSYEDSLFKDGGKEEKKKSPTSSSVKCYFVRMESLAPCDETESYILSKKTVYEAR-------- :   59
CsRDR5b : ------------------------------------------MMSKMKAEQDQGLYLNSTRTHLQR-------- :   24
CsRDR6a : -------------------------------------------------------------------------- :    -
CsRDR6b : FDLLDDDDPWIRTTDFTPSGAIGRCNTYRISIPPRCGVKLRKAMGYFRDRRVPDESPKQQLRILDEPDFGKPMS :  421
CsRDR6c : FDLLDDDDPWIRTTDFTPSGAIGRCNTYRISIPPRCGVKLRKAMGYFRDRRVPDESPKQQLRIRDEPDFGKPMS :  328
                                g

               *       460         *       480         *       500         *       5
CsRDR1c : P-----PQGLELPYQILFRICMLVQQGCIPGPALDINFFRLVDPKRNDIACVEYALEKLIHLKECCYEPVRWLS :  377
CsRDR1a : P-----PHELDLPYKILFKICILVQQGYLAGPTLDNNFYRLVNPQRMNIALIEHALEKLYHSKECCYEPVRWLV :  333
CsRDR1b : P-----PHGLEMPYKILFKICILVQQGCLPGQALDINFFRLVDPRRINIAFVEHALEKLYHLKECCYEPVRWLD :  331
CsRDR2  : C-----QSDSKLAYEILFQLNSLVHTQKISLASVDNDLIEILSS--LDIDTAMMVLQKMHKLQSTCYEPVSFIK :  374
CsRDR5a : ----------CLFMHVQMVSSLTKYMPRFS--------LVLSKTIKLQVDLASITIKDIEDIPCRDENGCVVYN :  115
CsRDR5b : ----------ELRDQIVLIVKFVEETTSCN--------NWVTGSSNYDVAFNKI-----------AEEGILVGL :   69
CsRDR6a : -------------------------------------------------------------------------- :    -
CsRDR6b : DPFFCIKSEKELTFKELFLVNAVMHKGIINQHLLSDKIFDLLRSQTEEVN--LAALKHICSYKRPVFDAYWRLK :  493
CsRDR6c : DPFFCIKSEKELTFKELFLVNAVMHKGIINQHLLSDRVFDLLRSQTEEVN--LAALKHICSYKRPVFDAYRRLK :  400

          20         *       540         *       560         *       580         *
CsRDR1c : EQFIELRKSRQHLRTPAIALD----DGLVNVRRVQITPCKVYFCGPEINMSNRVLRHYHEDIDNFLRISFLDEE :  447
CsRDR1a : EQYIKYPTLRKLPKTPDITLD----NGLMYVHRVQITPCKVFFSGPEVNMSNRVLRHYSEYIDNFLRVSFVDEE :  403
CsRDR1b : EQYRKYLTLKRLPRTPAITLD----GGLVYVHRVQITPCKVYFSGPEVNMSNRVLRHYSEDIDNFLRVSFVDEE :  401
CsRDR2  : TRLHILKKNSNGKNLPSLSLSRLTNHNTMSCHRVLITPSKIYCLGPELESSNYVVKKFASYASDFLRVTFVEED :  448
CsRDR5a : EDREPLIHTDGTG---------------------YISVDIALKAPKDFSRAKNDVRGNTYCFLSWERVFTFDLK :  168
CsRDR5b : RRFRFFVFKDGG------------------------------KEEKKKSPTSSIVK---CYFVRMESLAPCDEI :  110
CsRDR6a : -------------------------------------------------------------------------- :    -
CsRDR6b : LVQEWLLKNPKLLKSLTKLDD------VVEVRRLVITPTKAYCLPPTVELSNRVIRNYKEVADRFLRVTFMDEG :  561
CsRDR6c : LVQEWLLKNPKLLKSLTKLDD------VVEVRRLVITPTKAYCLPPTVELSNRVIRNYKEVADRFLRVTFMDEG :  468
                                             i                              r    de

               600         *       620         *       640         *       660
CsRDR1c : LDKMHSTDLSPRIAS-------SDEERRTAIYKRILSTLRNGIVIGNKKFEFLAFSSSQLRENSAWMFASRPML :  514
CsRDR1a : FDKLFSTDLSPRVAS-------SNEETRTGIYRRILSILRDGIVIGNKKFDFLAFSSSQLKGNSVWMFASSDRV :  470
CsRDR1b : WGKLFSTDLSPRASS-------SNEDRRTSIYRRILSTLRDGIVIGNKKFDFLAFSSSQLRDNSAWMFASRSGL :  468
CsRDR2  : WSKLSSNAISTSIQQGI-----FAKPYRTNIYHRILSILREGIVIGDKRFQFLAFSASQLRSNAVWMFASNENV :  517
CsRDR5a : MCEVSN--------------------VQTGQQLYLCAYVQVVYVHMSHKEKSFDGNSS---CIASFLMKPSTLS :  219
CsRDR5b : ESYILS--------------------KRTVHEAR-CLFMHVQMVSS----------------LAKYMARNLNDL :  147
CsRDR6a : -------------------------------------------------------------------------- :    -
CsRDR6b : MQTLNSNVLHNYVASIVKDITSYSAPQKTAVFQRIKSIVSGGFKLCGRRYSFLAFSSNQLRGQSAWFFAEDKDI :  635
CsRDR6c : MQTLNSNVLNNYVASIVKDITSYSAPQKTSVFQRVKSILSDGFKLCGRTYSFLAFSANQLRDRSAWFFAEDEDI :  542
               s                      t    r
```

```
             *       680         *       700         *       720         *       740
CsRDR1c : TAADIRKWMGDFHRIRNVARYAARLGQSFSSSRETLSVGRDEFEI-IPDVEVVRGGIEYVFSDGIGKISADFAR :  587
CsRDR1a : TAADIRKWMGDFRRIRNVAKYAARLGQSFSSSRETLSVSRHEIEP-IPDVEVVTGGNKYVFSDGIGKISADFAQ :  543
CsRDR1b : TAADIRNWMGDFRQIRNVAKYAARLGQSFGSSTETLSVSRHEMEI-IPDVEVVTRGIKYVFSDGIGKISSDFGR :  541
CsRDR2  : KTEDIRDWMGCFNKIRSVSKCAARMGQLFSSSTQTLEISAQEVET-IPDVEITSDGVGYCFSDGIGKISNSFAR :  590
CsRDR5a : SANFVGEYLWTLERFLNHANCEQKSLELRGSEAHTREPP------------------------LLMQIRLFYNG :  269
CsRDR5b : EVNEFASLLSILEVFLDHANCEEKSLELRGSEAHTREPP------------------------LLMQIRLVYNG :  197
CsRDR6a : --------MGKFK-NRNIAKCAARMGQCFSSTYATVEVPPNEVNAELPDIKRN----NYVFSDGIGMITPDLAM :   61
CsRDR6b : SVLNIKDWMGKFT-DTNVAKCAARMGQCFSSTYATIEVPHKEVNFKLRDVKRN----GYVFSDGIGKMTPDLAM :  704
CsRDR6c : SVLDIKGWMGKFT-DRNVAKCAARMGQCFSSTYATIEVPHKEVNFKLPDVERN----GYVFSDGIGKMTPDLAK :  611
                  6g f      a  aa4 g2 f S   T      e      d         y fsdg6g 6

                   *       760         *       780         *       800         *
CsRDR1c : RVAVKCGLKSSTPSAFQIRYGGYKGVVAIDP--TSSIKMSLRRSMFKYESENTKLDVLAWSKYQPCFLNRQLIS :  659
CsRDR1a : SVAKKCGLEL-TPSAFQIRYGGYKGVVAVDP--SSSMKMSLRMSMSKYESDHTKLDVLRWSGYMPSFLNRQFIT :  614
CsRDR1b : RVAKKCGLKSSTTSAFQIRYGGYKGVVAVDP--NSSMKLSLRMSMSKYESDNTKLDVLAWSKYQPCFLNRQLIT :  613
CsRDR2  : QVAQKCGLNH-TPSAFQIRYGGYKGVITVDR--NSFRKLSLRSSMLKFESKNRMLNVTSWSESMPCYLNREIVS :  661
CsRDR5a : RAVKGTLLVNRKLPEKTIQVR------------SSMIKVETDSRLSNVQTFNSLEIVGISNKPKKNYLSRNLIA :  331
CsRDR5b : RAVKGTLLVNRKLPEKTIQVM------------SSMIKVEADSRLSNVQTFNSLEIVGIS-------------- :  245
CsRDR6a : EVAEKLQLTVNPPCAYQIRYAGCKGVISCWPGKNDAIRLSLRQSMIKFESSHTVLEICSWTRFQPGFLNRQIIT :  135
CsRDR6b : EVAERLQLCVNPPCAYQIRYAGCKGVIACWPGKNEKIRLSLRESMNKFESKHAILEICSWTRFQPGFLNRQIIT :  778
CsRDR6c : EVAERLQLCVNPPSAYQIRYAGCKGVIACWPGKNEKIRLSLRKSMNKFESKHAILEICSWTRFQPGFLNRQIIT :  685
           va    L      a qIry g kgv           46slr s6 k 23    l 6  w    p  lnr

             820         *       840         *       860         *       880
CsRDR1c : LLSTLGVRDRIFEKKQKEEVDQLDTILTDPLRAQEALELMAPGENTNILKEMLMCGYKPDAEPFLSMMLQTFRS :  733
CsRDR1a : LLSTLGVEDHIFEKRQSEVIEELDNMLTDSLRAWEALELMCTGENTNILKEMLLCGYKPDAEPFLSMMLQTFRA :  688
CsRDR1b : LLSTLGVRDHIFEKKQREAIEELNTILTDPLMAQEALELMSPGENTNILKEMLLCGYKPDAEPFLSMMLRAFRA :  687
CsRDR2  : LLSTLGVEDTAFEAMQDQQLCLLGKMLTSKEAALDVLESMGGRDAKGILVKMLLQGYEPNMEPYLSMILRAHHD :  735
CsRDR5a : LLSYGGVPKKYFLDILTNALEDAQCVYSNKRTALKVAINHGEMDDNFTVAQMILSGT-PLDEPYIQYRLSVLAK :  404
CsRDR5b : -------------------------------------------------------------------------- :    -
CsRDR6a : LLSTLKVADETFWKMQQSMICKLNQMTVNTEVAFDVLTASCAEQ-GNTAAIMLSAGFKPQTELHLRGMLTCIRA :  208
CsRDR6b : LLSALNVPDEIFWTMQESMISKLDKMLKDADVAFDVLTTSCAEQ-GNVGAMMLSAGFNPQNEPHLRGMLTCIRA :  851
CsRDR6c : LLSALNVPDEIFWKMQESMISKLDKMLKDADVAFDVLTASCAEQ-GNVGAMMLSAGFNPQNEPHLRGMLTCIRA :  758
          lls l v d  f   q      l         a   l              m   g  p  ep     l

           *       900         *       920         *       940         *       960
CsRDR1c : AKLLELRTRTRIFVPKGRSMMGCLDETRTLEYGQVFVQFSGAGDR--------MHGDSGSSQRKFIVEGKVVVA :  799
CsRDR1a : AKLLGLRTKARIFVPKGRFMLGCLDETRTLEYGQVFVQISGVGHRAFYDDSLLMPSDSDSDQPNFIVQGKVFVA :  762
CsRDR1b : AKLLEIRTKTRIFVPRGRSMMGCLDETRTLEYGQVFVQISGVGHRPFYDNSLLMLGGCGSDQHNRIVEGKVVVA :  761
CsRDR2  : NQLSDLRTRCRIFVPQGRILIGCLDETGILNYGQVYVRITMTKAELQAGE---QSFFQKIDETTSVLIGKVVVT :  806
CsRDR5a : EVRKGLKG-GKLPISESFYLMGTADPTEELNSDEVCVIL-----------------------DNGQISGQVLVY :  454
CsRDR5b : -------------------------------------------------------------------------- :    -
CsRDR6a : AQLWGLREKARIFVSSGRWLMGCLDELGVLEQGQCFVQVSSPTLENCFAN--HGSRFSGTKKNLLVIKGFVVIT :  280
CsRDR6b : AQLWGLREKSRIFVLSGRWLMGCLDELAVLEQGQCFIQVSTPSLENCFVN--HGSRFSKPKKNVQVIKGIVVIA :  923
CsRDR6c : AQLWGLREKSRIFVHSGRWLMGCLDELAVLEQGQCFIQVSNPSLENCFVN--HGSRFSKPKKNVQVIKGLVVIA :  830
            l         f   gr   gclde   l  g                                   g v

                 *       980         *      1000         *      1020         *
CsRDR1c : KNPCLHPGDVRVLRAVNIPALHH----MVDCVVFPQKGLR---------------------------------- :  835
CsRDR1a : KNPCLHPGDVRVLNAVNVPALHH----MVDCVVFPQKGLR---------------------------------- :  798
CsRDR1b : KNPCLHPGDVRVLSAVNVPALHH----MVDCVVFPQKGMRLVDADKHMSMFVSINLLIMRLPVDKHRFGLKATT :  831
CsRDR2  : KNPCLHPGDIRVLEAVYEVALEEK--GWVNCVIFPQKGER---------------------------------- :  844
CsRDR5a : RNPGLHFGDIHVLKAVYVKALEDFVGNAKYAIFFSTKGRR---------------------------------- :  494
CsRDR5b : -------------------------------------------------------------------------- :    -
CsRDR6a : KNPCLHPGDIRVLEAVDAPGLDH----LSDCLVFPQKGDR---------------------------------- :  316
CsRDR6b : KNPCLHPGDIRILEAVDAPGLHH----LVDCVVFPQKGDR---------------------------------- :  959
CsRDR6c : KNPCLHPGDIRILEAVDAPGLHH----LVDCVVFPQKGDR---------------------------------- :  866
           npclhpgd r l av    l         c  fpqkg r

          1040         *      1060         *      1080         *      1100         *
CsRDR1c : -------------PHPNECSGSDLDGDIYFVSWDSDLIPP--RQIKPMDYTPAQSMLLDHDVTIE--------- :  885
CsRDR1a : -------------PHPNECSGSDLDGDIYFVCWDPDLIPP--QQVKPMDYTSAPSMLLDHDVTIESYALFLLLF :  857
CsRDR1b : AASCGTASTYIILPHPNECSGSDLDGDIYFVCWDPDLIPP--QQIQPMDYTPAPSMLSDHDVTIESKEGWRSYQ :  903
CsRDR2  : -------------PHPNECSGGDLDGDLYFISWNENLIPP--QTVTPMDYTGRRPRLMDHDVTLE--------- :  894
CsRDR5a : -------------SVGSEIANGDFDGDMYWVSRNPELLRHFKPSYPWSRIYSTPNACNTKKPSELSAEELEHEL :  555
CsRDR5b : -------------------------------------------------------------------------- :    -
CsRDR6a : -------------PHTNEASGSDLDGDLYFVTWDENLIPPSKRSWVPMDYTPAEVKESPRTVTQR--------- :  368
CsRDR6b : -------------PHTNEASGSDLDGDLYFATWDENLIPPSKSSYPPMDYEPGDVKKLPRPVNQK--------- : 1011
CsRDR6c : -------------PHTNEASGSDLDGDLYFATWDENLIPPSKRSYPPMDYEPGDVKKLPRPVNQK--------- :  918
                       ph ne sg dldgd y   w   l pp      pmdy           v

                1120         *      1140         *      1160         *      1180
CsRDR1c : -----------------------EVEEYFTNYILNDSLGIIANAHTVFADR--EPNKAMSDPCIRLAELFSIAV :  934
CsRDR1a : VISCSS----TFFFLLLVHRLIEEVQEYFANYIVNNSLGIISNAHTVFADK--EPSKAMSDPCIALAKLSSIAV :  925
CsRDR1b : SMPQHSLGVMDPSNRDLNDHDRHEVEEYFTNYIVNDSLGIIANAHTVFADT--EPLKAMSDPCIKLAQLFSIAV :  975
CsRDR2  : -----------------------EIHKFFVDYMINDTLGTISTAHLIHADR--EPSKALSSKCLQLATLHSMAV :  943
CsRDR5a : FQLFKR----------------TRFQQSYNMSVAADSWLAFMDRLLILGDSCSDEKDSMKETMHHLIDIYYEAL :  613
CsRDR5b : ----------------------------YNMSVAADSWLAFVDQLLILGDSSSVEKDRMKETMHHLIDIYCEAL :  291
CsRDR6a : -----------------------DIIDFFSKNMVNENLGAICNAHVVHADL--SEYGALDEKCITLAELAATAV :  417
CsRDR6b : -----------------------DIIDFFTRNIVNENLGAICNAHVVHADC--SEDGAQDEKCLTLAEKAATAV : 1060
CsRDR6c : -----------------------DIIDFFTRNIVNENLGTICNAHVVHADR--SEYGALDEKCLTLAEKAATAV :  967
                                            5   6 n  lg i  ah 6 aD       a    c  La     A6

               *      1200         *      1220         *      1240         *      12
CsRDR1c : DFPKTGVPAEIPSDLRVKEYPDFMEKPDRTTYESERVIGKLFRAVKDIAPHLNSIESFTREVARKS-YDPDMEV : 1007
CsRDR1a : DFAKTGVPAEIPPDLRVKEYPDFMEKSDKSTYESKRVLGKLFRAVKDISPQSSSIKSFTKEVAENS-YDPDMEV :  998
CsRDR1b : DFPKTGVPAEIPTELRVKIYPDFMEKPDKTTYESESVIGKLFRAVKDISPQSSSFKSFTRDVARNS-YDPDMEV : 1048
CsRDR2  : DFAKTGAPAEMPRFLKPKEFPDFMQRWDKPNYISKGVLGKLYRATVNSPVHESAVLVWSEKIAQDA-YDHDLIV : 1016
CsRDR5a : DAPKSGKKKCDTDLLLPLPTGILDPLDFEVVIPDQLKAEKFPHYMGKPNKYHSTSVLGLIYEMVESFQSQDLSA :  687
CsRDR5b : DAPKSGKK---------------------------------THFNALP---------------------FVLSC :  311
CsRDR6a : DFPKTGQLVTMPSQLKPKMYPDFMGKEPFQSYKSKKILGRLYRQIKDAYDNDVEAPSELTFVPGDIPYDTDLEI :  491
CsRDR6b : DFPKTGKIVTMPSELKPKMYPDFMGKEAYRSYKSKKILGRLYRKITDAYDEDVEAYPEQTFVPESIPYDGDLEI : 1134
CsRDR6c : DFPKTGKIVTMPPKLKPKMYPDFMGKEAFRSYKSEKILGRLYRKITDAYDEDVEAYPEQTFVPESIPYDGDLEI : 1041
          DfpK3G     p  l  k  pdfm       y s    g l r                        yd d6

          60         *      1280         *      1300         *      1320         *
CsRDR1c : DGFEDYIDDAYDYKNEYDFKLGNLMDYYGIKTEAEILGGSIMKMSKTFDKRRD-----SEAVGLAVRSLRKEAR : 1076
CsRDR1a : DGFEDYIDDAFDCKNVYDDKLGNLMDCYGIQTEGEILSGSIMTMSKFFDQKKA-----EKDVGFAVRSLRKEAR : 1067
CsRDR1b : DGFEDYIDDAFDYKSEYDYKLGNLMDYYGIKTEAEILSGSIMKMSKSFDRRRD-----AEDVGLAVRSLRKEAR : 1117
CsRDR2  : NGFEDFIEVAQSHKEMYLDKMSTLMNFYEAESEDEILTGNLRTKSAYLLRDNRRYGEMKDRILVSVKSLYKEAK : 1090
CsRDR5a : KDVEVWKLPCFDVEIPTSCMK--------LWGERYSQYRCEMTEAMNLDDESK-------NDAANVVTKRYKQL :  746
CsRDR5b : MWISTFKAPAF------------------------------MVMPRNLGYGPD-------NTSAALKS------ :  342
CsRDR6a : PGSIDFIDDAWSHKCSYDGQLNGLLGQYKVNREEEVVTGHIWSMPKYNSRQRG---ELQEKLKHAYSALRKEFR :  562
CsRDR6b : PGSMDFIADAWEHKCSYDGQLNGLLGQYKVVREEEIVTGHIWSMPKYHSRKRG---DLQEKLKHAYNSLRKELR : 1205
CsRDR6c : PGSVDFIADAWEHKCSYDGQLNGLLGQYKVVREEEIVTGHIWSMPKYHSRKRG---DLQEKLKHAYNSLRKEFR : 1112
           g  d5i  a   k  y      l   y    e e   g    m                    a   lrke

              1340         *      1360         *      1380         *      1400
CsRDR1c : TWFNRKEAASGVGTDDVY-----AKASAWYHVTYHPRYWGCYNEGMN-----REHFLSFPWCVYDKLIHIKRDK : 1140
CsRDR1a : SWFNTSGSESDAETDDVY-----AKASAWYYVTYHPSYWGRSNEGTD-----REHFLSFPWCVYDKLIHIKKEK : 1131
CsRDR1b : TWFNTSRATSDAESDDVY-----AKASAWYHVTYHPSYWGQYNGGMN-----RDHFLSFPWCVYDKLIHIKKEK : 1181
CsRDR2  : GWFESS-----CKADQQQ-----KMASAWYHVTYHPSYCKES-----------IHCLGFPWILGDILLNIKSVQ : 1143
CsRDR5a : LYGAAEFEESPRNTEDIYN-----EALAIYNITYDHAMGYRNVKNCG----------FAWRVAGSALFKLHAIK :  805
CsRDR5b : -------------------------------------------------------------------------- :    -
CsRDR6a : QNFEKIS-DVSQLSDDEKNTLYEGKASAWYQVTYHPSWVKKSLEIQESNGTDQMLMLSFCWIAADYLARIKIRC :  635
CsRDR6b : QAFEKMGSDFDQLSDDAKNTMYERKASAWYQVAYHPSWVKKSIELHDSDGSGKMVMLSFSWIAADYLARIKIRR : 1279
CsRDR6c : QAFEKMGLDFNQLSDDAKNTMYERKASAWYQVAYHPSWVKKSIELQESDGSGKMVMLSFSWIAADYLARIKIRR : 1186
            f           dd         asawy   yhp                    l f w   d l   k

             *      1420         *
CsRDR1c : SSI---RRALHLSSLERQFSYGLSLS- : 1163
CsRDR1a : S-----RRLLHMSSVEKQLSSGLSLG- : 1152
CsRDR1b : S-----RMALQLSSLQRRLQYGLSLG- : 1202
CsRDR2  : S---------------RKLSA------ : 1149
CsRDR5a : QSEKMEKPIVCLPSVLREVLN------ :  826
CsRDR5b : --------------------------- :    -
CsRDR6a : RGIENFDSTKPINSLVKYLADRI---- :  658
CsRDR6b : REIGNLDTTKPINSLAKYLADRDAIGN : 1306
CsRDR6c : REMGNLDTTKPINSLAKYFADRDAIGY : 1213
                       s
```

Supplementary Figure S2-a . The NJ phylogenetic tree of AGO family genes in 62 representative plant species.

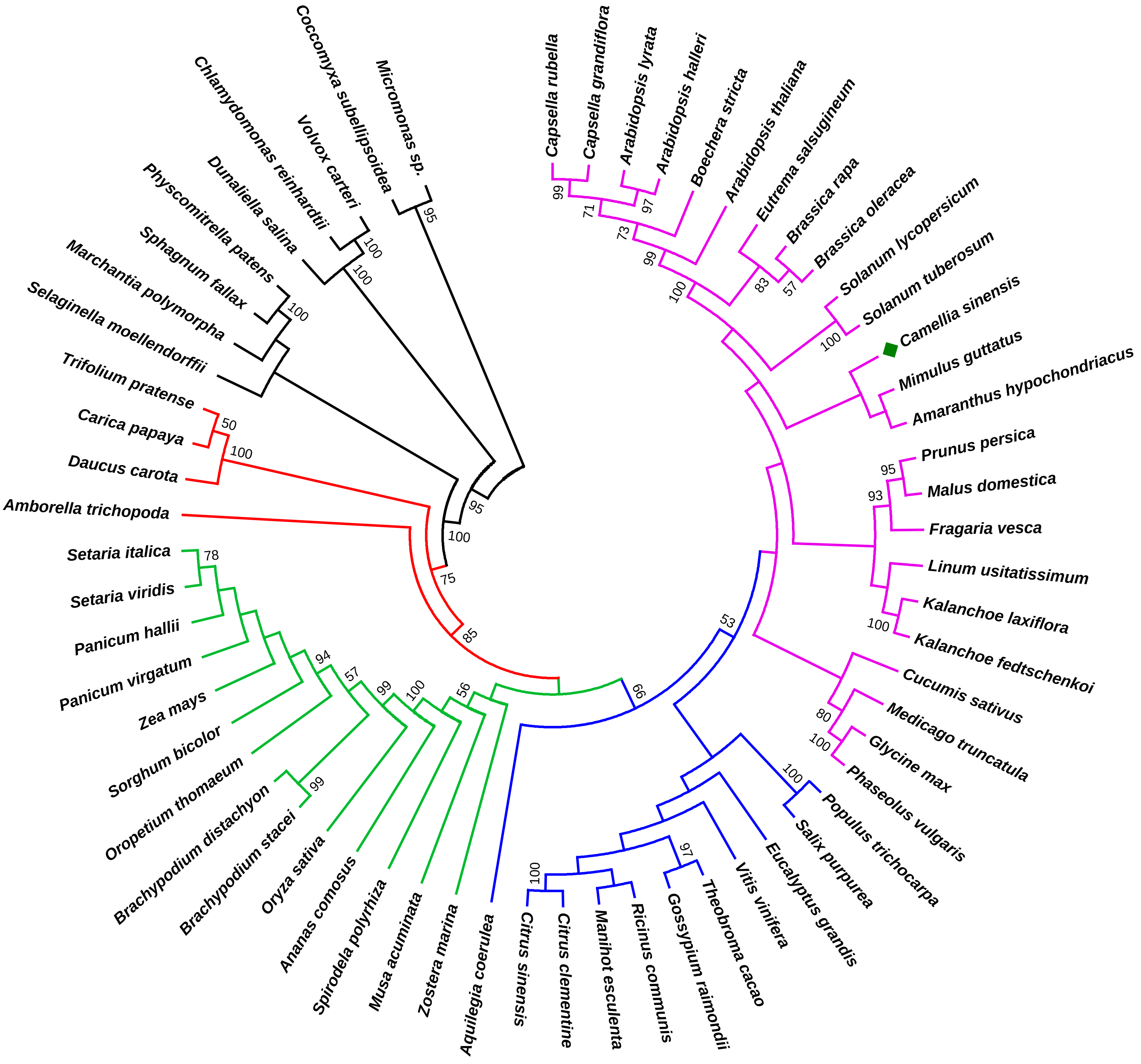

Supplementary Figure S2-b. The NJ phylogenetic tree of DCL family genes in 62 representative plant species

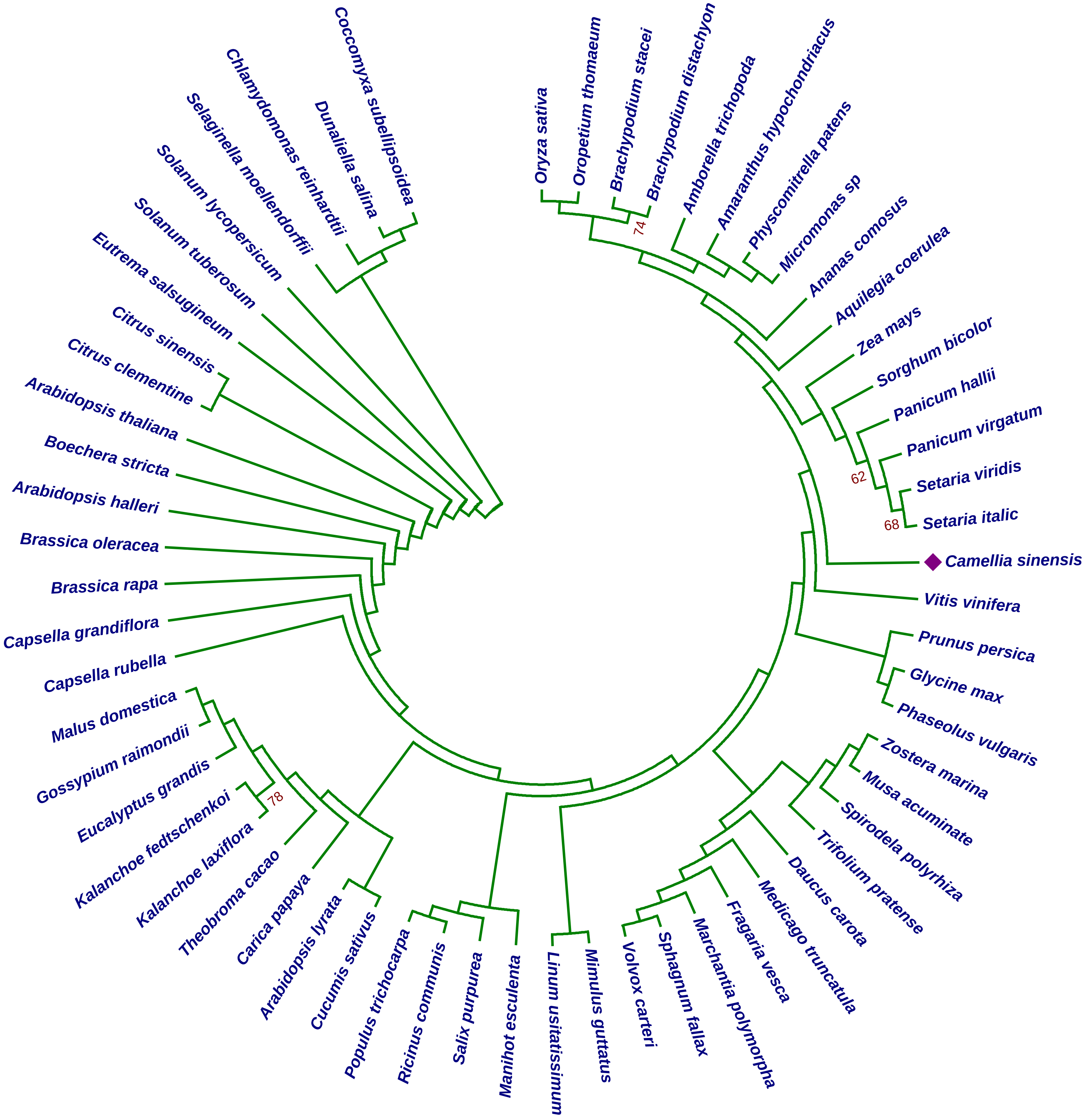

Supplementary Figure S2-c: The NJ phylogenetic tree of RDR family genes in 59 representative plant species

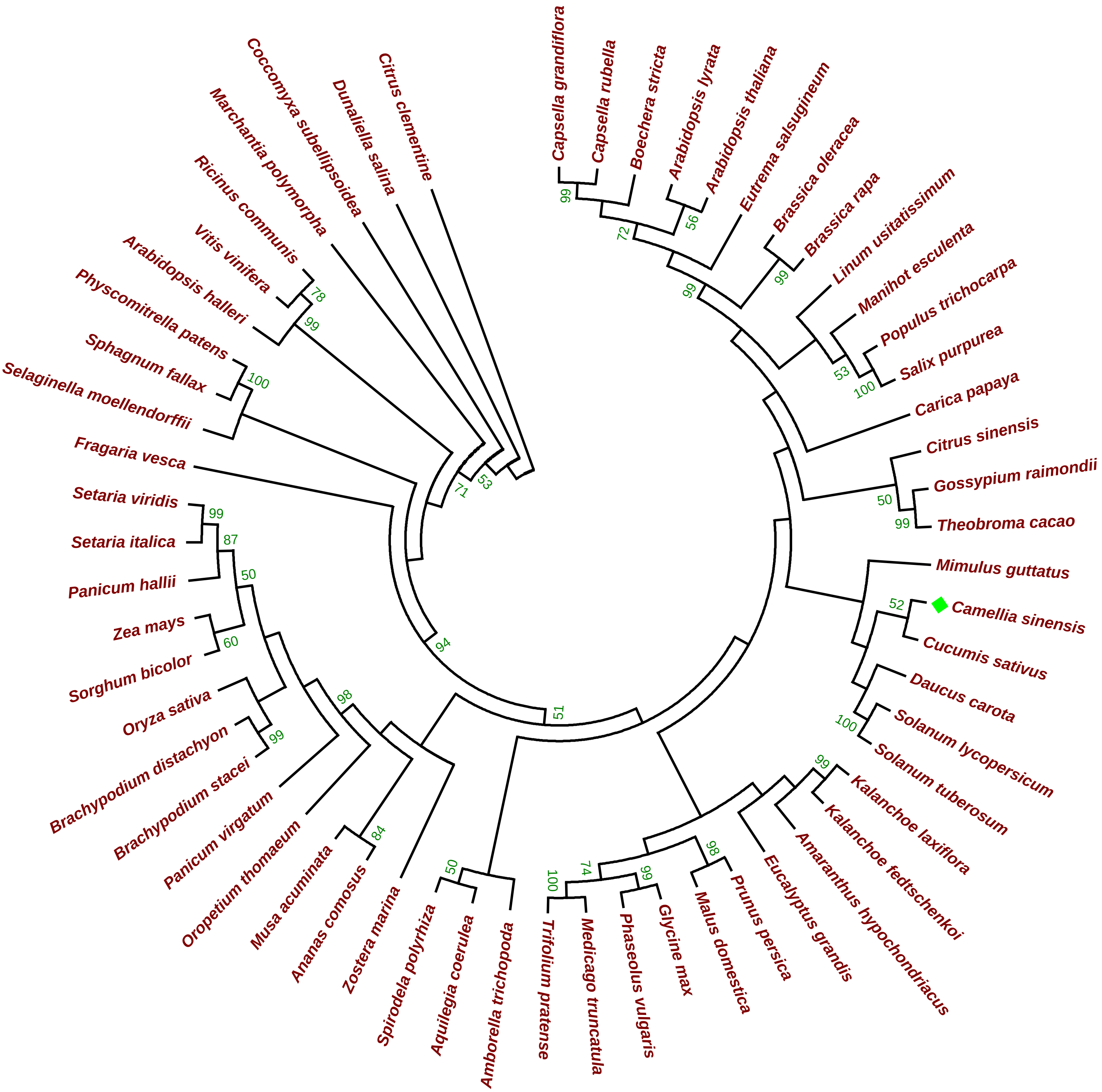

Supplementary Figure S3-a. Schematic diagram showing the logos of conserved motifs identified in CsAGO encoded proteins.

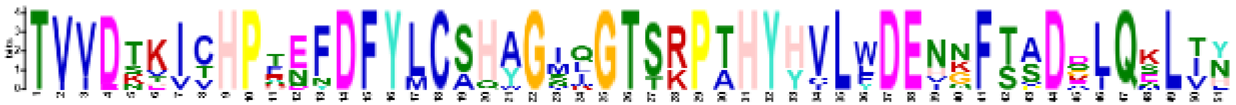

Motif 1

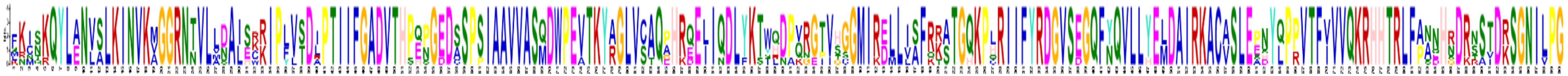

Motif 2

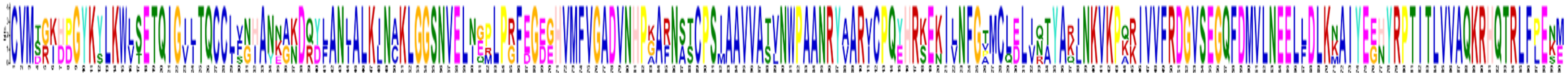

Motif 3

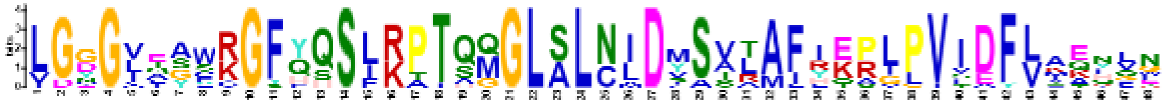

Motif 4

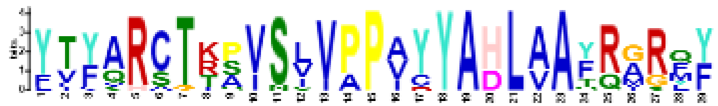

Motif 5

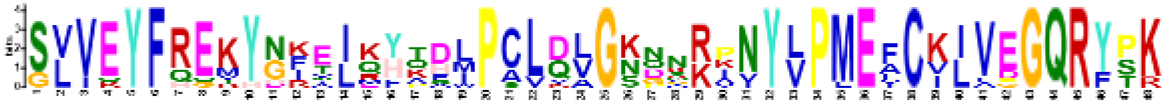

Motif 6

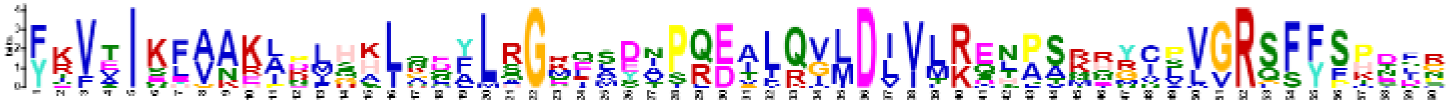

Motif 7

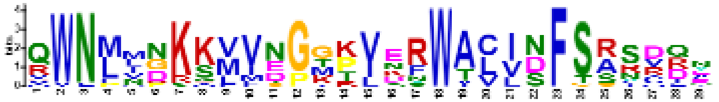

Motif 8

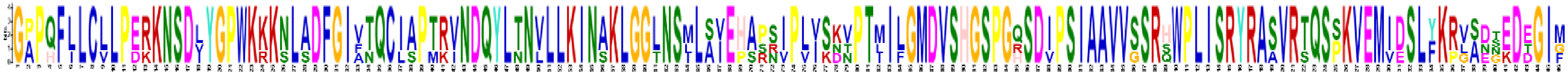

Motif 9

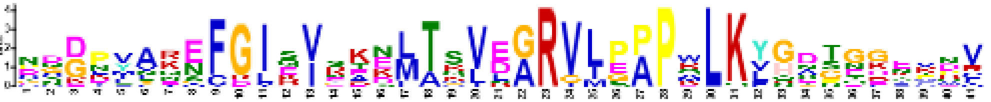

Motif 10

Supplementary Figure S3-b. Schematic diagram showing the logos of conserved motifs identified in CsDCL encoded proteins.

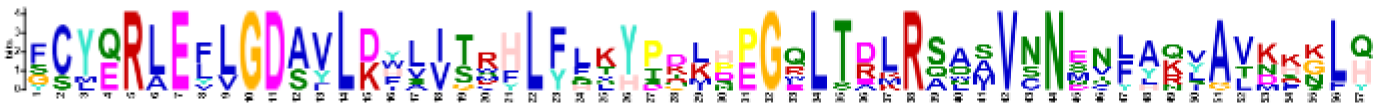

Motif 1

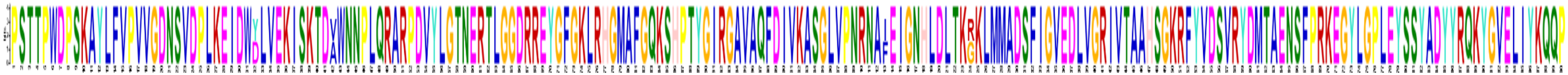

Motif 2

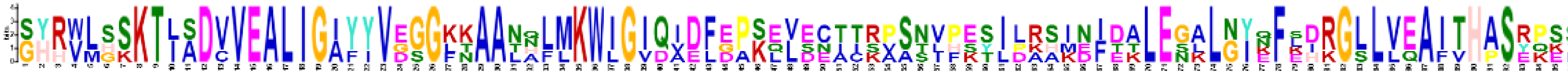

Motif 3

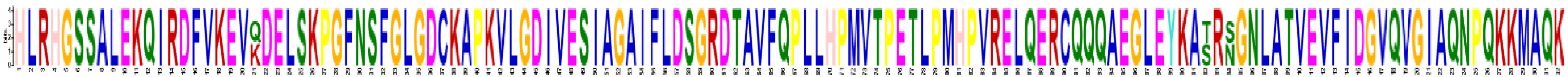

Motif 4

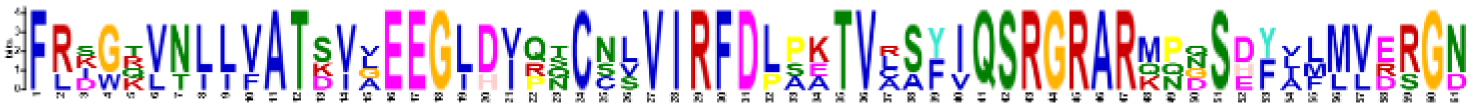

Motif 5

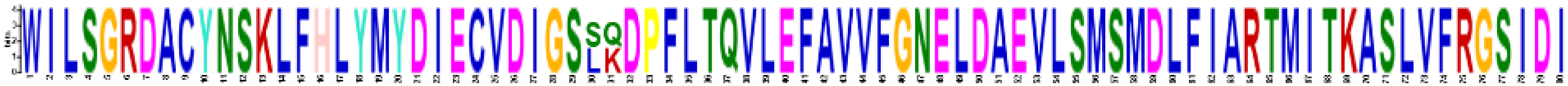

Motif 6

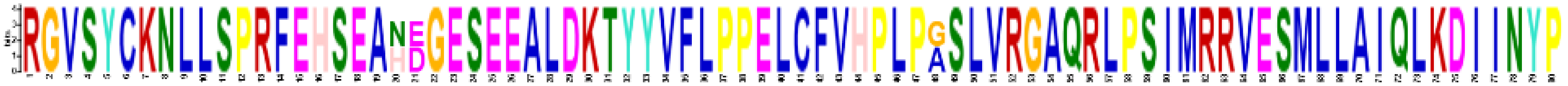

Motif 7

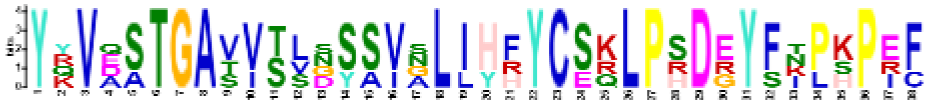

Motif 8

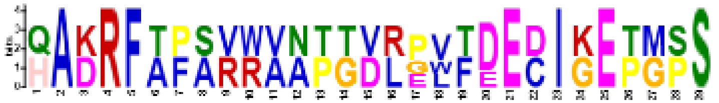

Motif 9

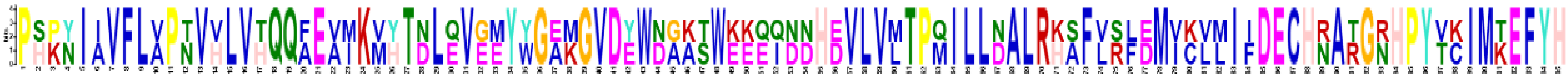

Motif 10

Supplementary Figure S3-c. Schematic diagram showing the logos of conserved motifs identified in CsRDR encoded proteins.

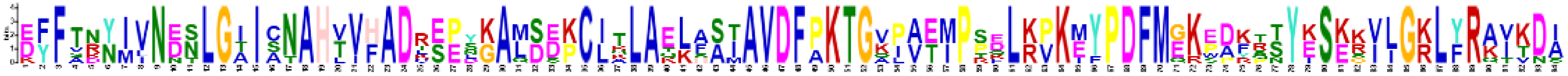

Motif 1

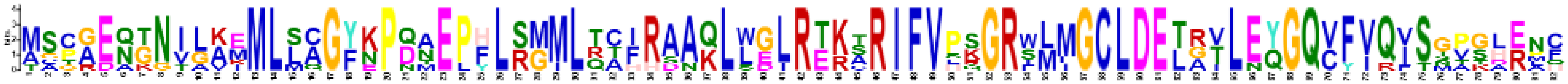

Motif 2

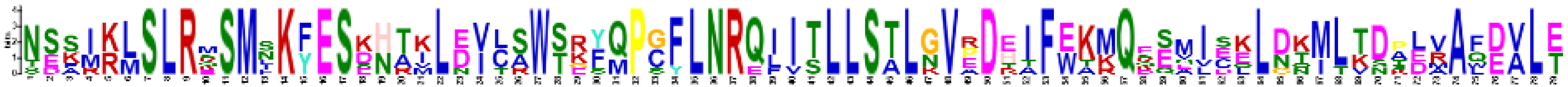

Motif 3

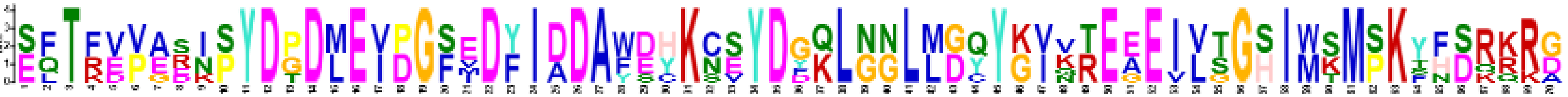

Motif 4

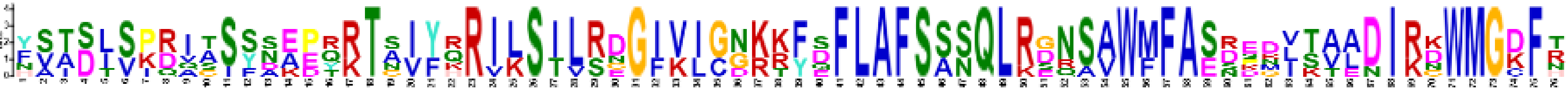

Motif 5

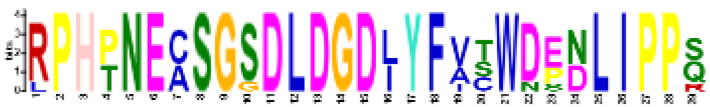

Motif 6

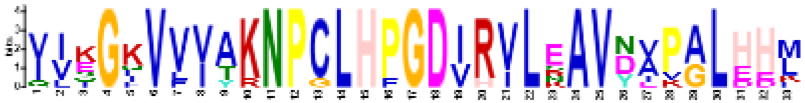

Motif 7

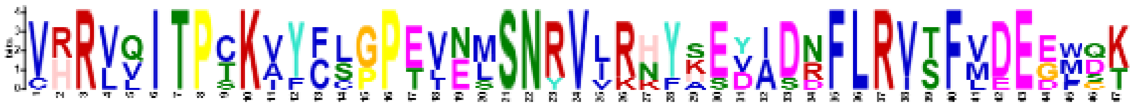

Motif 8

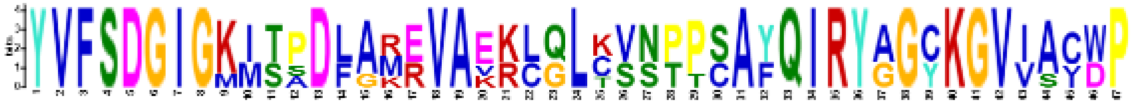

Motif 9

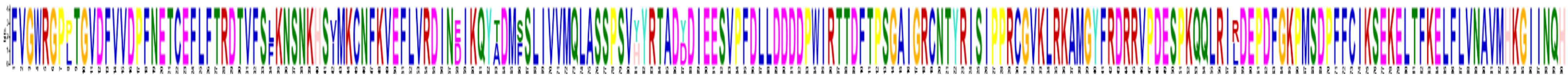

Motif 10

Supplementary Table S1-a. List of orthologous genes of AGOs from all plant lineages used to construct phylogenetic tree

Sl. No.	Plant Species Name	Protein ID	Score#	E value	Description *
1.	*Marchantia polymorpha*	Mapoly0001s0149.1	1496.9	0.0	Argonaute linker 1 domain
2.	*Physcomitrella patens*	Pp3c1_35770V3.6	1450.3	0.0	Argonaute linker 1 domain
3.	*Sphagnum fallax*	Sphfalx0001s0279.1	1458.4	0.0	Argonaute linker 1 domain
4.	*Selaginella moellendorffii*	110151	1459.9	0.0	Argonaute linker 1 domain
5.	*Ananas comosus*	Aco011930.1	1559.7	0.0	Argonaute linker 1 domain
6.	*Amborella trichopoda*	evm_27.model.AmTr_v1.0_scaffold00044.24	1547.7	0.0	Argonaute linker 1 domain
7.	*Musa acuminata*	GSMUA_AchrUn_randomT09260_001	1603.2	0.0	Argonaute linker 1 domain
8.	*Spirodela polyrhiza*	Spipo17G0036500	1588.2	0.0	Argonaute linker 1 domain
9.	*Zostera marina*	Zosma25g01490.1	1493.0	0.0	Argonaute linker 1 domain
10.	*Brachypodium distachyon*	Bradi5g18540.1	1557.0	0.0	Argonaute linker 1 domain
11.	*Brachypodium stacei*	Brast09G173700.1	1558.9	0.0	Argonaute linker 1 domain
12.	*Oryza sativa*	LOC_Os04g47870.2	1565.4	0.0	Argonaute linker 1 domain
13.	*Oropetium thomaeum*	Oropetium_20150105_01399A	1533.1	0.0	Argonaute linker 1 domain
14.	*Panicum hallii*	Pahal.G01956.1	1564.3	0.0	Argonaute linker 1 domain
15.	*Panicum virgatum*	Pavir.Ga01012.1	1557.0	0.0	Argonaute linker 1 domain
16.	*Setaria italica*	Seita.7G201100.1	1559.3	0.0	Argonaute linker 1 domain
17.	*Setaria viridis*	Sevir.7G213000.2	1562.4	0.0	Argonaute linker 1 domain
18.	*Sorghum bicolor*	Sobic.006G181000.1	1534.6	0.0	Argonaute linker 1 domain
19.	*Zea mays*	GRMZM2G039455_T01	1542.3	0.0	Argonaute linker 1 domain
20.	*Aquilegia coerulea*	Aqcoe3G009200.1	1623.6	0.0	Argonaute linker 1 domain
21.	*Amaranthus hypochondriacus*	AHYPO_012431-RA	1648.6	0.0	Argonaute linker 1 domain
22.	*Daucas carota*	DCAR_027248	1332.8	0.0	Argonaute linker 1 domain
23.	*Mimulus guttatus*	Migut.O00245.1	1672.5	0.0	Argonaute linker 1 domain
24.	*Solanum lycopersicum*	Solyc06g072300.2.1	1709.9	0.0	ARGONAUTE 1
25.	*Solanum tuberosum*	PGSC0003DMT400069313	1708.4	0.0	ARGONAUTE 1
26.	*Kalanchoe fedtschenkoi*	Kaladp0053s0463.1	1629.0	0.0	Argonaute linker 1 domain
27.	*Kalanchoe laxiflora*	Kalax.0373s0030.1	1632.1	0.0	Argonaute linker 1 domain
28.	*Eucalyptus grandis*	Eucgr.K02304.1	1674.1	0.0	Argonaute linker 1 domain
29.	*Vitis vinifera*	GSVIVT01029383001	1689.9	0.0	Argonaute linker 1 domain
30.	*Linum usitatissimum*	Lus10031331	1655.6	0.0	Argonaute linker 1 domain
31.	*Manihot esculenta*	Manes.02G219700.1	1681.8	0.0	Argonaute linker 1 domain
32.	*Populus trichocarpa*	Potri.012G037100.1	1681.0	0.0	Argonaute linker 1 domain
33.	*Ricinus communis*	29677.m000188	1707.6	0.0	Protein with all the AGO specific domains
34.	*Salix purpurea*	SapurV1A.0202s0350.1s	1676.0	0.0	Argonaute linker 1 domain

35.	*Citrus sinensis*	orange1.1g001466m	1679.8	0.0	Argonaute linker 1 domain
36.	*Citrus clementina*	Ciclev10018625m	1679.8	0.0	Argonaute linker 1 domain
37.	*Carica papaya*	evm.model.supercontig_44.130	1434.5	0.0	Argonaute linker 1 domain
38.	*Gossypium raimondii*	Gorai.008G294100.1	1663.7	0.0	Argonaute linker 1 domain
39.	*Theobroma cacao*	Thecc1EG013468t2	1677.1	0.0	Argonaute linker 1 domain
40.	*Arabidopsis halleri*	Araha.2117s0011.2	1574.7	0.0	Argonaute linker 1 domain
41.	*Arabidopsis lyrata*	AL1G55040.t1	1575.5	0.0	Argonaute linker 1 domain
42.	*Arabidopsis thaliana*	AT1G48410.2	1562.0	0.0	Argonaute linker 1 domain
43.	*Boechera stricta*	Bostr.25219s0559.1	1578.5	0.0	Argonaute linker 1 domain
44.	*Brassica oleracea capitata*	Bol043177	1545.8	0.0	Argonaute linker 1 domain
45.	*Brassica rapa*	Brara.H00354.1	1552.3	0.0	Argonaute linker 1 domain
46.	*Capsella grandiflora*	Cagra.1131s0020.1	1578.5	0.0	Argonaute linker 1 domain
47.	*Capsella rubella*	Carubv10008158m	1581.2	0.0	Argonaute linker 1 domain
48.	*Eutrema salsugineum*	Thhalv10011197m	1541.9	0.0	Argonaute linker 1 domain
49.	*Cucumis sativus*	Cucsa.112480.1	1638.2	0.0	Argonaute linker 1 domain
50.	*Fragaria vesca*	mrna09290.1-v1.0-hybrid	1578.5	0.0	Argonaute linker 1 domain
51.	*Glycine max*	Glyma.16G217300.1	1662.1	0.0	Argonaute linker 1 domain
52.	*Malus domestica*	MDP0000069525	1642.9	0.0	Argonaute linker 2 domain
53.	*Medicago trunculata*	Medtr6g477980.2	1628.6	0.0	Argonaute linker 1 domain
54.	*Phaseolus vulgaris*	Phvul.004G142900.1	1642.5	0.0	Argonaute linker 1 domain
55.	*Prunus persica*	Prupe.5G241600.2	1689.5	0.0	Argonaute linker 1 domain
56.	*Trifolium pratense*	Tp57577_TGAC_v2_mRNA26982	1261.5	0.0	Argonaute linker 1 domain
57.	*Chlamydomonas reinhardtii*	Cre16.g689647.t1.1	434.9	2.5E-131	Argonaute linker 1 domain
58.	*Dunaliella salina*	Dusal.0754s00004.1	449.9	3.6E-138	Argonaute linker 1 domain
59.	*Volvox carteri*	Vocar.0052s0031.1	412.9	2.8E-123	Argonaute linker 1 domain
60.	*Coccomyxa subellipsoidea*	56024	430.6	3.3E-130	Argonaute linker 1 domain
61.	*Micromonas sp. RCC299*	113410	334.3	4.3E-96	Argonaute linker 1 domain

#Smith-Watermann alignment score (based on BLOSUM45, with gap opening and extension penalties of -12 and -2 respectively)

*All protein sequences show presence of typical AGO specific domains viz. PAZ, PIWI, Argo-N, Argo-L1, Argo-Mid and Argo-L2. However, *Dunaliella salina* and *Volvox carteri* exhibited absence of miD domain, whereas PAZ domain was absent in *Micromonas* sp.

Supplementary Table S1-b. List of orthologous genes of DCLs from all plant lineages used to construct phylogenetic tree

Sl. No.	Plant Species Name	Protein ID	Score#	E value	Description *
1.	*Marchantia polymorpha*	Mapoly0003s0222.1	2016.1	0.0	ENDORIBONUCLEASE DICER HOMOLOG 1
2.	*Physcomitrella patens*	Pp3c2_15900V3.1	1979.5	0.0	ENDORIBONUCLEASE DICER HOMOLOG 1
3.	*Sphagnum fallax*	Sphfalx0020s0155.1	1958.3	0.0	ENDORIBONUCLEASE DICER HOMOLOG 1
4.	*Selaginella moellendorffii*	86110	1793.5	0.0	ENDORIBONUCLEASE DICER HOMOLOG 1
5.	*Ananas comosus*	Aco016650.1	2366.3	0.0	ENDORIBONUCLEASE DICER HOMOLOG 1
6.	*Amborella trichopoda*	evm_27.model.AmTr_v1.0_scaffold00018.3	2265.7	0.0	ENDORIBONUCLEASE DICER HOMOLOG 1
7.	*Musa acuminata*	GSMUA_Achr8T12350_001	1069.3	0.0	ENDORIBONUCLEASE DICER HOMOLOG 1
8.	*Spirodela polyrhiza*	Spipo1G0078500	2244.5	0.0	ENDORIBONUCLEASE DICER HOMOLOG 1
9.	*Zostera marina*	Zosma109g00390.1	2227.6	0.0	ENDORIBONUCLEASE DICER HOMOLOG 1
10.	*Brachypodium distachyon*	Bradi1g77087.1	2248.0	0.0	ENDORIBONUCLEASE DICER HOMOLOG 1
11.	*Brachypodium stacei*	Brast02G020300.1	2255.7	0.0	ENDORIBONUCLEASE DICER HOMOLOG 1
12.	*Oryza sativa*	LOC_Os0302970.1	2290.8	0.0	ENDORIBONUCLEASE DICER HOMOLOG 1
13.	*Oropetium thomaeum*	Oropetium_20150105_22551A	2213.0	0.0	ENDORIBONUCLEASE DICER HOMOLOG 1
14.	*Panicum hallii*	Pahal.I00170.1	2259.6	0.0	ENDORIBONUCLEASE DICER HOMOLOG 1
15.	*Panicum virgatum*	Pavir.Ib00062.1	2255.3	0.0	ENDORIBONUCLEASE DICER HOMOLOG 1
16.	*Setaria italica*	Seita.9G562200.1	2248.4	0.0	ENDORIBONUCLEASE DICER HOMOLOG 1
17.	*Setaria viridis*	Sevir.9G565900.1	2252.3	0.0	ENDORIBONUCLEASE DICER HOMOLOG 1
18.	*Sorghum bicolor*	Sobic.001527800.2	2271.1	0.0	ENDORIBONUCLEASE DICER HOMOLOG 1
19.	*Zea mays*	GRMZM2G040762_T01	1961.0	0.0	ENDORIBONUCLEASE DICER HOMOLOG 1
20.	*Aquilegia coerulea*	Aqcoe5G472300.2	2374.4	0.0	ENDORIBONUCLEASE DICER HOMOLOG 1
21.	*Amaranthus hypochondriacus*	AHYPO_01582-RA	2272.3	0.0	ENDORIBONUCLEASE DICER HOMOLOG 1
22.	*Daucas carota*	DCAR_022119	2273.4	0.0	ENDORIBONUCLEASE DICER HOMOLOG 1
23.	*Mimulus guttatus*	Migut.I00117.1	2330.4	0.0	ENDORIBONUCLEASE DICER HOMOLOG 1
24.	*Solanum lycopersicum*	Solyc10g005130.2.1	2238.8	0.0	ENDORIBONUCLEASE DICER HOMOLOG 1
25.	*Solanum tuberosum*	PGSC0003DMT400029301	2269.2	0.0	ENDORIBONUCLEASE DICER HOMOLOG 1
26.	*Kalanchoe fedtschenkoi*	Kaladp0072s0008.1	1321.2	0.0	ENDORIBONUCLEASE DICER HOMOLOG 1
27.	*Kalanchoe laxiflora*	Kalax.0054s0136.1	2265.7	0.0	ENDORIBONUCLEASE DICER HOMOLOG 1
28.	*Eucalyptus grandis*	Eucgr.D02653.1	2424.1	0.0	ENDORIBONUCLEASE DICER HOMOLOG 1
29.	*Vitis vinifera*	GSVIVT01027462001	1863.2	0.0	ENDORIBONUCLEASE DICER HOMOLOG 1

30.	*Linum usitatissimum*	Lus10005142bt	1635.2	0.0	ENDORIBONUCLEASE DICER HOMOLOG 1
31.	*Manihot esculenta*	Manes.05G015200.1	2449.5	0.0	ENDORIBONUCLEASE DICER HOMOLOG 1
32.	*Populus trichocarpa*	Potri.002G181400.1	2422.1	0.0	ENDORIBONUCLEASE DICER HOMOLOG 1
33.	*Ricinus communis*	30076.m004468	2470.7	0.0	ENDORIBONUCLEASE DICER HOMOLOG 1
34.	*Salix purpurea*	SapurV1A.0003s0350.1	2400.6	0.0	ENDORIBONUCLEASE DICER HOMOLOG 1
35.	*Citrus sinensis*	orange1.1g000174m	2351.6	0.0	ENDORIBONUCLEASE DICER HOMOLOG 1
36.	*Citrus clementina*	Ciclev10018447m	2428.7	0.0	ENDORIBONUCLEASE DICER HOMOLOG 1
37.	*Carica papaya*	evm.model.supercontig_57.74	2153.6	0.0	ENDORIBONUCLEASE DICER HOMOLOG 1
38.	*Gossypium raimondii*	Gorai.001150400.1	2418.3	0.0	ENDORIBONUCLEASE DICER HOMOLOG 1
39.	*Theobroma cacao*	Thecc1EG005016t2	2441.8	0.0	ENDORIBONUCLEASE DICER HOMOLOG 1
40.	*Arabidopsis halleri*	Araha.2490s0029.1	2239.9	0.0	ENDORIBONUCLEASE DICER HOMOLOG 1
41.	*Arabidopsis lyrata*	AL1G11480.t1	2238.8	0.0	ENDORIBONUCLEASE DICER HOMOLOG 1
42.	*Arabidopsis thaliana*	AT1G01040.2	2216.4	0.0	ENDORIBONUCLEASE DICER HOMOLOG 1
43.	*Boechera stricta*	Bostr.5325s0024.1	2231.5	0.0	ENDORIBONUCLEASE DICER HOMOLOG 1
44.	*Brassica oleracea capitata*	Bol040724	2245.3	0.0	ENDORIBONUCLEASE DICER HOMOLOG 1
45.	*Brassica rapa*	Brara.J00078.1	2253.8	0.0	ENDORIBONUCLEASE DICER HOMOLOG 1
46.	*Capsella grandiflora*	Cagra.1968s0049.1	2273.8	0.0	ENDORIBONUCLEASE DICER HOMOLOG 1
47.	*Capsella rubella*	Carubv10008073m	2273.8	0.0	ENDORIBONUCLEASE DICER HOMOLOG 1
48.	*Eutrema salsugineum*	Thhalv10006531m	2224.1	0.0	ENDORIBONUCLEASE DICER HOMOLOG 1
49.	*Curcumis sativus*	Cucsa.260100.1	2430.6	0.0	ENDORIBONUCLEASE DICER HOMOLOG 1
50.	*Fragaria vesca*	mrna18596.1-v1.0-hybrid	2333.5	0.0	ENDORIBONUCLEASE DICER HOMOLOG 1
51.	*Glycine max*	Glyma.19G261200.2	2417.1	0.0	ENDORIBONUCLEASE DICER HOMOLOG 1
52.	*Malus domestica*	MDP0000619907	199.5	1.7E-52	DICER-LIKE PROTEIN 4
53.	*Medicago trunculata*	Medtr7g118350.1	2398.6	0.0	ENDORIBONUCLEASE DICER HOMOLOG 1
54.	*Phaseolus vulgaris*	Phvul.009G260000.1	2409.0	0.0	ENDORIBONUCLEASE DICER HOMOLOG 1
55.	*Prunus persica*	Prupe.2G200900.1	2419.0	0.0	ENDORIBONUCLEASE DICER HOMOLOG 1
56.	*Trifolium pratense*	Tp57577_TGAC_v2_mRNA4956	2400.9	0.0	ENDORIBONUCLEASE DICER HOMOLOG 1
57.	*Chlamydomonas reinhardtii*	Cre02.g141000.t1.1	97.1	3.5E-19	ENDORIBONUCLEASE DICER HOMOLOG 1
58.	*Dunaliella salina*	Dusal.0147s00002.1	74.7	2E-12	ENDORIBONUCLEASE DICER HOMOLOG 1
59.	*Volvox carteri*	Vocar.0003s0040.1	73.9	1E-13	ENDORIBONUCLEASE DICER HOMOLOG 1
60.	*Coccomyxa subellipsoidea*	33474	50.1	2E-6	Ribonuclease III
61.	*Micromonas sp. RCC299*	98380	52.4	1.4E-6	DEAD-BOX ATP-DEPENDENT RNA HELICASE 40

#Smith-Watermann alignment score (based on BLOSUM45, with gap opening and extension penalties of -12 and -2 respectively)

Supplementary Table S1-c. List of orthologous genes of RDRs from all plant lineages used to construct phylogenetic tree

Sl. No.	Plant Species Name	Protein ID	Score#	E value	Description *
1.	*Marchantia polymorpha*	Mapoly0151s0009.1	592.0	0.0	RNA-DEPENDENT RNA POLYMERASE
2.	*Physcomitrella patens*	Pp3c24_15800V3.6	844.3	0.0	RNA-DEPENDENT RNA POLYMERASE 1
3.	*Sphagnum fallax*	Sphfalx0166s0014.1	873.6	0.0	RNA-DEPENDENT RNA POLYMERASE 1
4.	*Selaginella moellendorffii*	150191	761.1	0.0	RNA-DEPENDENT RNA POLYMERASE 1
5.	*Ananas comosus*	Aco002958.1	1293.9	0.0	RNA-DEPENDENT RNA POLYMERASE 1
6.	*Amborella trichopoda*	evm_27.model.AmTr_v1.0_scaffold00176.15	1251.9	0.0	RNA-DEPENDENT RNA POLYMERASE 1
7.	*Musa acuminata*	GSMUA_Achr10T29960_001	1115.5	0.0	RNA-DEPENDENT RNA POLYMERASE 1
8.	*Spirodela polyrhiza*	Spipo15G0038000	1087.4	0.0	RNA-DEPENDENT RNA POLYMERASE 1
9.	*Zostera marina*	Zosma121g00800.1	1154.0	0.0	RNA-DEPENDENT RNA POLYMERASE 1
10.	*Brachypodium distachyon*	Bradi3g59910.1 (	1122.8	0.0	RNA-DEPENDENT RNA POLYMERASE 1
11.	*Brachypodium stacei*	Brast04G010200.1	1189.1	0.0	RNA-DEPENDENT RNA POLYMERASE 1
12.	*Oryza sativa*	LOC_Os02g50330.1	922.5	0.0	RNA-DEPENDENT RNA POLYMERASE 1
13.	*Oropetium thomaeum*	Oropetium_20150105_04127A	1180.2	0.0	RNA-DEPENDENT RNA POLYMERASE 1
14.	*Panicum hallii*	Pahal.A03315.1	1196.4	0.0	RNA-DEPENDENT RNA POLYMERASE 1
15.	*Panicum virgatum*	Pavir.Aa00750.1	1132.5	0.0	RNA-DEPENDENT RNA POLYMERASE 1
16.	*Setaria italica*	Seita.1G312200.1	1209.1	0.0	RNA-DEPENDENT RNA POLYMERASE 1
17.	*Setaria viridis*	Sevir.1G318100.1	1205.7	0.0	RNA-DEPENDENT RNA POLYMERASE 1
18.	*Sorghum bicolor*	Sobic.004G246400.1	1182.9	0.0	RNA-DEPENDENT RNA POLYMERASE 1
19.	*Zea mays*	GRMZM2G481730_T01	1048.9	0.0	RNA-DEPENDENT RNA POLYMERASE 1
20.	*Aquilegia coerulea*	Aqcoe6G285700.1	1330.5	0.0	RNA-DEPENDENT RNA POLYMERASE 1
21.	*Amaranthus hypochondriacus*	AHYPO_003322-RA	1256.5	0.0	RNA-DEPENDENT RNA POLYMERASE 1
22.	*Daucas carota*	DCAR_030283	1358.2	0.0	RNA-DEPENDENT RNA POLYMERASE 1
23.	*Mimulus guttatus*	Migut.L01176.1	1432.2	0.0	RNA-DEPENDENT RNA POLYMERASE 1
24.	*Solanum lycopersicum*	Solyc05g007510.2.1	1397.5	0.0	RNA-DEPENDENT RNA POLYMERASE 1
25.	*Solanum tuberosum*	PGSC0003DMT400080319d	1384.4	0.0	RNA-DEPENDENT RNA POLYMERASE 1
26.	*Kalanchoe fedtschenkoi*	Kaladp0036s0212.1	1299.3	0.0	RNA-DEPENDENT RNA POLYMERASE 1
27.	*Kalanchoe laxiflora*	Kalax.0097s0091.1	1310.8	0.0	RNA-DEPENDENT RNA POLYMERASE 1
28.	*Eucalyptus grandis*	Eucgr.G02093.1	1325.8	0.0	RNA-DEPENDENT RNA POLYMERASE 1
29.	*Vitis vinifera*	GSVIVT01007792001	807.0	0.0	RNA-DEPENDENT RNA POLYMERASE 2

30.	*Linum usitatissimum*	Lus10019164	1356.7	0.0	RNA-DEPENDENT RNA POLYMERASE 1
31.	*Manihot esculenta*	Manes.15G135600.1	1445.3	0.0	RNA-DEPENDENT RNA POLYMERASE 1
32.	*Populus trichocarpa*	Potri.008G36100.1	1441.8	0.0	RNA-DEPENDENT RNA POLYMERASE 1
33.	*Ricinus communis*	30147.m013981	780.4	0.0	RNA-DEPENDENT RNA POLYMERASE 2
34.	*Salix purpurea*	Solanum SapurV1A.0015s0570.1	1387.1	0.0	RNA-DEPENDENT RNA POLYMERASE 1
35.	*Citrus sinensis*	orange1.1g002586m	1240.3	0.0	RNA-DEPENDENT RNA POLYMERASE 1
36.	*Citrus clementina*	Ciclev10007331m	95.9	6E-18	RNA-DEPENDENT RNA POLYMERASE
37.	*Carica papaya*	evm.model.supercontig _2.177	955.3	0.0	RNA-DEPENDENT RNA POLYMERASE 1
38.	*Gossypium raimondii*	Gorai.013G029000.1	1397.1	0.0	RNA-DEPENDENT RNA POLYMERASE 1
39.	*Theobroma cacao*	Thecc1EG011292t1	1387.9	0.0	RNA-DEPENDENT RNA POLYMERASE 1
40.	*Arabidopsis halleri*	Araha.9628s0001.1	645.2	0.0	RNA-DEPENDENT RNA POLYMERASE 2
41.	*Arabidopsis lyrata*	AL1G26820.t1	1332.8	0.0	RNA-DEPENDENT RNA POLYMERASE 1
42.	*Arabidopsis thaliana*	AT1G14790.1	1322.8	0.0	RNA-DEPENDENT RNA POLYMERASE 1
43.	*Boechera stricta*	Bostr.0124s0180.1	1250.7	0.0	RNA-DEPENDENT RNA POLYMERASE 1
44.	*Brassica oleracea capitata*	Bol038076	1315.1	0.0	RNA-DEPENDENT RNA POLYMERASE 1
45.	*Brassica rapa*	Brara.F01003.1	1314.7	0.0	RNA-DEPENDENT RNA POLYMERASE 1
46.	*Capsella grandiflora*	Cagra.3501s0013.1	1340.1	0.0	RNA-DEPENDENT RNA POLYMERASE 1
47.	*Capsella rubella*	Carubv10008144m	1337.0	0.0	RNA-DEPENDENT RNA POLYMERASE 1
48.	*Eutrema salsugineum*	Thhalv10006629m	1307.4	0.0	RNA-DEPENDENT RNA POLYMERASE 1
49.	*Cucumis sativus*	Cucsa.143700.1	1340.1	0.0	RNA-DEPENDENT RNA POLYMERASE 1
50.	*Fragaria vesca*	mrna06498.1-v1.0-hybrid	1152.1	0.0	RNA-DEPENDENT RNA POLYMERASE 1
51.	*Glycine max*	Glyma.02G086100.1	1341.3	0.0	RNA-DEPENDENT RNA POLYMERASE 1
52.	*Malus domestica*	MDP0000475729	1335.1	0.0	RNA-DEPENDENT RNA POLYMERASE 1
53.	*Medicago truncatula*	Medtr1g032630.1	1335.1	0.0	RNA-DEPENDENT RNA POLYMERASE 1
54.	*Phaseolus vulgaris*	Phvul.003G016600.2	1295.8	0.0	RNA-DEPENDENT RNA POLYMERASE 1
55.	*Prunus persica*	Prupe.1G334600.2	1357.8	0.0	RNA-DEPENDENT RNA POLYMERASE
56.	*Trifolium pratense*	Tp57577_TGAC_v2_m RNA38119	1272.3	0.0	RNA-DEPENDENT RNA POLYMERASE 1
57.	*Dunaliella salina*	Dusal.0696s00003.1	196.4	1E-51	RNA-DEPENDENT RNA POLYMERASE
58.	*Coccomyxa subellipsoidea*	20637	486.9	1.5E-155	RNA-DEPENDENT RNA POLYMERASE

#Smith-Watermann alignment score (based on BLOSUM45, with gap opening and extension penalties of -12 and -2 respectively)

Supplementary Table S2-a. Annotation of conserved MEME motifs identified in CsAGO proteins.

Motif No.	Width	Sequence	e-value	Pfam Annotation
1	51	TVVDTKICHPTEFDFYLCSHAGIQGTSRPTHYHVLWDENNFTA DDLQKLIY	2.2e-545	Piwi domain
2	200	FKISKQYLANVSLKINVKMGGRNTVLLDAJSKRIPLVSDIPTIIFG ADVTHPZPGEDSSPSIAAVVASQDWPEVTKYAGLVCAQP HRZEJIQDLYKTWQDPQRGTVHGGMIRELLISFRKATGQKPLRII FYRDGVSEGQFYQVLLYELDAIRKACASLEPNYQPPVTFVVVQ KRHHTRLFAANHRDRNSTDRSGNILPG	1.2e-787	Piwi domain
3	200	CVMSGKHPGYKYLKWLSETQIGIJTQCCLFNHANKAKDQYLAN JALKINAKLGGSNVELNEPLPRFEGEGHVMFVGADVNHPGARN STCPSIAAVVATVNWPAANRYAARVCPQYHRKEKILNFGTMCL ELIQTYAKINKVKPKRIVVFRDGVSEGQFDMVLNEELLDLKKAI YEEHYRPTITLVVAQKRHQTRLFPENM	4.1e-518	Piwi domain
4	48	LGGGVEAWRGFYQSLRPTQQGLSLNJDMSVTAFIEPLPVIDFLA ZNLN	1.4e-356	Argo-L1 domain
5	29	YTYARCTKPVSLVPPAYYAHLAAYRARQY	1.8e-242	Unknown
6	48	SVVEYFREKYNKEJKYTDLPCLDLGKNNRPNYLPMEFCKJVEG QRYPK	2.4e-306	PAZ domain
7	60	FKVTIKFAAKLDLHKLKEYLRGKQSDIPQEALQVLDJVLRENPS KRYCPVGRSFFSPDFR	1.2e-342	Argo-N domain
8	29	QWNLLNKKVVNGGKVERWACINFSRSDQN	3.8e-225	Unknown
9	146	GPPQFLLCLLPERKNSDJYGPWKKKNLADFGIVTQCIAPTRVND QYLTNVLLKINAKLGGLNSMLAIEHAPSIPLISKVPTMILGMDVS HGSPGQSDIPSIAAVVSSRHWPLISRYRASVRTQSPKVEMIDSLY KRVADSEDEGIM	1.2e-207	Piwi domain
10	41	NDDPVAKEFGIEVSKELTSVEGRVLPPPRLKYGBTGGEVNV	1.2e-200	Argo-L2 domain

Supplementary Table S2-b. Annotation of conserved MEME motifs identified in CsDCL proteins.

Motif No.	Width	Sequence	e-value	Pfam Annotation
1	57	FCYZRLEFLGDAVLDYLITRHLFLKYPDLHPGQLTDLRSAAVNN ENFAKVAVKKGLH	3.9e-157	RNaseIII domain
2	200	PSTTPWDPSKAYLFVPVVGDNSVDPLKEIDWDLVEKISKTDVW NNPLQRARPDVYLGTNERTLGGDRREYGFGKLRHGMAFGQKS HPTYGIRGAVAQFDIVKASGLVPNRNAFEIGNHLDLTKGKLMM ADSFIGVEDLVGRIVTAAHSGKRFYVDSVRYDMTAENSFPRKE GYLGPLEYSSYADYYRQKYGVELIYKQQP	1.0e-089	PAZ domain
3	98	GHRWLSSKTJADVVEALIGAYYVEGGKKAANQLMKWIGIQIDF EPKELECTTRPSNVPESILRSINFDALEGALGYEFKDRGLLVEAIT HASRPSSGV	4.2e-062	Unknown
4	132	HLRHGSSALEKQIRDFVKEVKDELSKPGFNSFGLGDCKAPKVL GDIVESIAGAIFLDSGRDTAVFQPLLHPMVTPETLPMHPVRELQE RCQQQAEGLEYKASRNGNLATVEVFIDGVQVGIAQNPQKKMA QK	2.7e-053	Unknown
5	61	FRKGKVNLLVATSVAEEGLDIQSCNLVIRFDLPKTVASYIQSRG RARMPDSDYILMVERGN	2.9e-051	Helicase C-terminal domain
6	80	WILSGRDACYNSKLFHLYMYDIECVDIGSLKDPFLTQVLEFAVV FGNELDAEVLSMSMDLFIARTMITKASLVFRGSIDI	2.9e-031	Unknown
7	80	RGVSYCKNLLSPRFEHSEANEGESEEALDKTYYVFLPPELCFVH PLPGSLVRGAQRLPSIMRRVESMLLAIQLKDIINYP	5.0e-027	PAZ domain
8	38	YKVESTGAVVSLNSSVGLIHFYCSKLPSDEYFTPKPEF	5.2e-025	Unknown
9	29	QADRFAPAVWVAPGDRPVTDEDIGEPGPS	7.3e-020	Unknown
10	105	PHKNIIVFLVPNVVLVHQQFEVIKMHTDLZVEEYWGEKGVDEW DGKSWEEZQDDHEVLVLTPQILLDALRKAFLRFEMICLLIFDEC HRARGRHPYVCIMKEFYH	1.8e-019	DEAD box helicase

Supplementary Table S2-c. Annotation of conserved MEME motifs identified in CsRDR proteins.

Motif No.	Width	Sequence	e-value	Pfam Annotation
1	94	EFFTNYIVNDSLGIICNAHVVHADREPYKALSEKCJTLAELASIA VDFPKTGVPAEMPSELKPKEYPDFMEKEDKTSYESKKVLGKLY RAIKDA	3.0e-258	RdRP domain
2	82	MSCGENGNILAEMLLAGYKPDAEPFLSMMLTCFRAAQLWGLR TKSRIFVPSGRWLMGCLDETRVLEYGQVFVQISGPGLENC	2.7e-226	RdRP domain
3	79	NSSIKLSLRMSMSKFESKHTKLDVLSWSRYQPGFLNRQIITLLST LGVPDEIFEKMQEEMIEKLDKMLTDPEVAFDVLE	7.1e-193	RdRP domain
4	70	EFTFEPASIPYDPDLEIPGFEDYIDDAWDHKCSYDGKLGGLLGQ YGIKTEEEILTGHIWSMPKYFDRKRG	3.9e-145	Unknown
5	76	YSTDJSPRIASYNEPRRTAIYRRILSILRDGIVIGNKKFDFLAFSSS QLRDNSAWMFASDEDITAADIRDWMGDFR	2.2e-130	RdRP domain
6	29	RPHPNECSGSDLDGDJYFVTWDEBLIPPQ	7.3e-110	RdRP domain
7	33	VIKGKVVVAKNPCLHPGDIRVLEAVDVPALHHL	2.0e-109	RdRP domain
8	47	VHRVQITPCKVYFLGPEVEMSNRVJRHYKEYIDNFLRVSFVDEE WDK	2.3e-096	RdRP domain
9	47	YVFSDGIGKITPDLAREVAEKLQLKVNPPSAYQIRYAGCKGVIA CWP	9.3e-092	RdRP domain
10	200	FVGWRGPPTGVDFVVDPFNETCEFLFTRDTVFSFKNSNKHSIMK CNFKVEFLVRDINEIKQYADMFSLIVVMQLASSPSVHYRTADD DIEESVPFDLLDDDDPWIRTTDFTPSGAIGRCNTYRISIPPRCGVK LRKAMGYFRDRRVPDESPKQQLRIRDEPDFGKPMSDPFFCIKSE KELTFKELFLVNAVMHKGIINQH	1.9e-091	Unknown

Supplementary Table S3: Putative miRNA target sites present in the identified genes

Target Gene	miRNA Accession	Expect value	Target Accessibility (UPE)	Alignment	Inhibition
CsAGO2/3a	ath-miR5658	2.0	8.597	miRNA 21 AAAGUAGUAGUAGUAGUAGUA 1<br>::::::.::.::.::.::::<br>Target 3074 CUUCAUCGUCGUCGUCGUCAU 3094	Cleavage
	mtr-miR2673a	2.0	21.717	miRNA 22 CACCUUCUCCUUCUCCUUCUCC 1<br>:::: .:::::::::::::.::<br>Target 86 GUGGUGGAGGAAGAGGAAGGGG 107	Cleavage
	mtr-miR2673b	2.0	21.717	miRNA 22 CACCUUCUCCUUCUCCUUCUCC 1<br>:::: .:::::::::::::.::<br>Target 86 GUGGUGGAGGAAGAGGAAGGGG 107	Cleavage
CsAGO2/3c	mtr-miR2673a	1.5	23.783	miRNA 22 CACCUUCUCCUUCUCCUUCUCC 1<br>: :::::.:::::.:::::.::<br>Target 74 GCGGAAGGGGAAGGGGAAGGGG 95	Cleavage
	mtr-miR2673b	1.5	23.783	miRNA 22 CACCUUCUCCUUCUCCUUCUCC 1<br>: :::::.:::::.:::::.::<br>Target 74 GCGGAAGGGGAAGGGGAAGGGG 95	Cleavage
CsAGO4a	ddi-miR1176	2.0	15.828	miRNA 21 CGAAAGGAACUAUUUUUAACC 1<br>:::::::::.:.::::::<br>Target 2389 AAGUUCCUUGAUGAGAAUUGG 2409	Cleavage
CsAGO5c	bdi-miR169c-3p	2.0	27.278	miRNA 20 CAUCGGUUCCUGUUGGACGG 1<br>: ::.::::.:::::::::<br>Target 1629 UUUGCUAAGGGCAACCUGCC 1648	Cleavage
CsAGO10b	ppt-miR536c	2.0	18.235	miRNA 22 UUUCGUGUUUCGAACCGUGCUA 1<br>:.::::: :::::::::::<br>Target 2256 CUUGUACAAAACUUGGCACGAU 2277	Cleavage
CsRDR1c	ghr-miR7508	2.0	20.043	miRNA 21 GAGAGGGCUGAAGAAAAGAAC 1<br>.:::.: ::.:::::::::::<br>Target 1313 UUCUUCGGAUUUCUUUUCUUG 1333	Cleavage
CsRDR6b	bol-miR9409	2.0	12.699	miRNA 22 CUUUUACGUCAGUACUUGUUUU 1<br>: .:::::::::::: :::::.<br>Target 1321 GUGAAUGCAGUCAUGCACAAAG 1342	Cleavage
CsRDR6c	bol-miR9409	2.0	12.853	miRNA 22 CUUUUACGUCAGUACUUGUUUU 1<br>: .:::::::::::: :::::.<br>Target 1042 GUGAAUGCAGUCAUGCACAAAG 1063	Cleavage

Supplementary Table S4: List of primers used for qRT-PCR analysis

Sl No	Name	Primer sequence	Annealing temperature (°C)	Product size (bp)
1	CsAGO1-F	GGATTTGCACCAATATGATGTTTCAATTAC	60	219
	CsAGO1-R	CCTGTTCCATCATCTTCATCAATGAGAG		
2	CsAGO2/3a-F	GAAGCTAAATGCTTACTTACGAGGAATGCTC	61	161
	CsAGO2/3a-R	CATAGCCAAGGTCTTCGTTTGGTCTG		
3	CsAGO2/3d-F	GTTGTTGAAGGAAATGTCGCTGATTC	60	249
	CsAGO2/3d-R	CTCCTGACGAGATTCCAATGACATTTC		
4	CsAGO5a-F	GTGAATGAAGAATTTGGTATTCAAGTGAGG	60	254
	CsAGO5a-R	CTACGACACATAGAAATCAATTCACGGC		
5	CsAGO6-F	GAGGAATTTCACTAATGTTGGAGGAGGTG	61.3	245
	CsAGO6-R	CCATGTTGCTGTGCCTTGTCTTAACC		
6	CsAGO7-F	CAGAATAACCCTCCTCTGGCTACCTCG	61.3	231
	CsAGO7-R	GAATTGGACTAGGAAATGATTGGCAAGG		
7	CsAGO10a-F	CAACATTAAACTTGTGGATGAAGAGGATGG	61.9	231
	CsAGO10a-R	GATCTTCCAACAGGGCAGTACCTCTTTG		
8	CsDCL1b-F	GGAAATCATTTGGATTTGACTAAACGC	59	210
	CsDCL1b-R	GTAATCAGCATAAGAGCTGTACTCCAATGG		
9	CsDCL1a-F	GCATGTACCAATGCCCTCAGAAATTG	60.5	221
	CsDCL1a-R	CACCATCACTTTCTGTTCTTTCAGAGACC		
10	CsDCL2-F	GAGGTAGACATCCCTATGTTTGCATTATG	58	225
	CsDCL2-R	GAATGTACTTGGCTAACACAGATTCGC		
11	CsDCL3-F	CAAATCTTCAAGTGGAAGAATACTATGGAGC	60	200
	CsDCL3-R	GTTACCAGTAGCACGATGGCATTCATC		
12	CsDCL4-F	GAGCACCAAGAAGATGCTCTGGAGAC	60	253
	CsDCL4-R	CTCCACACAGGATAAATCAGCTTCAATTC		
13	CsRDR1a-F	GATTTCACTCCATTATTCTGCCTTGGTC	60	178
	CsRDR1a-R	CCACAATGGGTACTAGATCCAAATTGC		
14	CsRDR1b-F	GAGAACATCTGGCAGATTGAGCTTCG	61	211
	CsRDR1b-R	GCTCCAAACATAAAGAAGAAGATTGGCC		
15	CsRDR5a-F	GACGATAAAACTTCAAGTCGATCTTGC	57	166
	CsRDR5a-R	GGGAGCCTTCAATGCAATATCTACTG		
16	CsRDR5b-F	CTCTTGCTCCTTGCGATGAGATAGAATC	60	227
	CsRDR5b-R	CGACCCTCTCAATTCCAGTGATTTCTC		
17	CsRDR6a-F	GTGGCTTTCGATGTTCTTACTGCTTC	58	250
	CsRDR6a-R	GGACTTGACACTTGAACAAAGCATTG		
18	CsRDR6b-F	GCAGATGTGGCGTTTGATGTTCTTAC	59	247
	CsRDR6b-R	CTTGGATAAAGCATTGACCTTGTTCG		
19	CsRDR6c-F	CATCATGTGCTGAGCAAGGAAATGTAG	59.6	232
	CsRDR6c-R	GAAGGATTCGATACTTGGATAAAGCATTG		
20	18S rRNA-F*	TCTGCCCGTTGCTCTGATG	60	134
	18S rRNA-R	TCACCCGTCACCACCATAG		
F= Forward and R=Reverse; *AF207029.1 (Accession No.)				
